# Unifying Splitting

**DOI:** 10.1007/s10817-023-09660-8

**Published:** 2023-04-28

**Authors:** Gabriel Ebner, Jasmin Blanchette, Sophie Tourret

**Affiliations:** 1 Vrije Universiteit Amsterdam, Amsterdam, The Netherlands; 2 Université de Lorraine, CNRS, Inria, LORIA, Nancy, France; 3 Max-Planck-Institut für Informatik, Saarland Informatics Campus, Saarbrücken, Germany

**Keywords:** Automated theorem proving, Completeness, Splitting, AVATAR

## Abstract

AVATAR is an elegant and effective way to split clauses in a saturation prover using a SAT solver. But is it refutationally complete? And how does it relate to other splitting architectures? To answer these questions, we present a unifying framework that extends a saturation calculus (e.g., superposition) with splitting and that embeds the result in a prover guided by a SAT solver. The framework also allows us to study *locking*, a subsumption-like mechanism based on the current propositional model. Various architectures are instances of the framework, including AVATAR, labeled splitting, and SMT with quantifiers.

## Introduction

One of the great strengths of saturation calculi such as resolution [26] and superposition [1] is that they avoid case analyses. Derived clauses hold unconditionally, and the prover can stop as soon as it derives the empty clause, without having to backtrack. The drawback is that these calculi often generate long, unwieldy clauses that slow down the prover. A remedy is to partition the search space by splitting a multiple-literal clause $C_1 \vee \cdots \vee C_n$ into subclauses $C_i$ that share no variables. Splitting approaches include splitting with backtracking [31, 32], splitting without backtracking [25], labeled splitting [15], and AVATAR [28].

The AVATAR architecture, which is based on a satisfiability (SAT) solver, is of particular interest because it is so successful. Voronkov reported that an AVATAR-enabled Vampire could solve 421 TPTP problems that had never been solved before by any system [28, Sect. 9], a mind-boggling number. Intuitively, AVATAR works well in combination with the superposition calculus because it combines superposition’s strong equality reasoning with the SAT solver’s strong clausal reasoning. It is also appealing theoretically, because it gracefully generalizes traditional saturation provers and yet degenerates to a SAT solver if the problem is propositional.

To illustrate the approach, we follow the key steps of an AVATAR-enabled resolution prover on the initial clause set containing $\lnot \textsf{p}(\textsf{a}),$ $\lnot \textsf{q}(z, z),$ and $\textsf{p}(x)\vee \textsf{q}(y, \textsf{b}).$ The disjunction can be split into $\textsf{p}(x)\mathbin {\leftarrow }\{[\textsf{p}(x)]\}$ and $\textsf{q}(y, \textsf{b})\mathbin {\leftarrow }\{[\textsf{q}(y, \textsf{b})]\},$ where $C \mathbin {\leftarrow }\{[C]\}$ indicates that the clause *C* is enabled only in models in which the associated propositional variable [*C*] is true. A SAT solver is then run to choose a model $\mathcal {J}$ of $[\textsf{p}(x)] \vee [\textsf{q}(y, \textsf{b})]$. Suppose $\mathcal {J}$ makes $[\textsf{p}(x)]$ true and $[\textsf{q}(y, \textsf{b})]$ false. Then resolving $\textsf{p}(x)\mathbin {\leftarrow }\{[\textsf{p}(x)]\}$ with $\lnot \textsf{p}(\textsf{a})$ produces $\bot \mathbin {\leftarrow }\{[\textsf{p}(x)]\}$, meaning that $[\textsf{p}(x)]$ must be false. Next, the SAT solver makes $[\textsf{p}(x)]$ false and $[\textsf{q}(y, \textsf{b})]$ true. Resolving $\textsf{q}(y, \textsf{b})\mathbin {\leftarrow }\{[\textsf{q}(y, \textsf{b})]\}$ with $\lnot \textsf{q}(z, z)$ yields $\bot \mathbin {\leftarrow }\{[\textsf{q}(y, \textsf{b})]\}$, meaning that $[\textsf{q}(y, \textsf{b})]$ must be false. Since both disjuncts of $[\textsf{p}(x)] \vee [\textsf{q}(y, \textsf{b})]$ are false, the SAT solver reports “unsatisfiable,” concluding the refutation.

What about refutational completeness? Far from being a purely theoretical concern, establishing completeness—or finding counterexamples—could yield insights into splitting and perhaps lead to an even stronger AVATAR. Before we can answer this open question, we must mathematize splitting. Our starting point is the *saturation framework* by Waldmann, Tourret, Robillard, and Blanchette [29], based on the work of Bachmair and Ganzinger [2]. It covers a wide array of techniques, but “the main missing piece of the framework is a generic treatment of clause splitting” [29, p. 332]. We provide that missing piece, in the form of a *splitting framework*, and use it to show the completeness of an AVATAR-like architecture. The framework is currently a pen-and-paper creature; a formalization using Isabelle/HOL [21] is underway.

Our framework has five layers, linked by refinement. The first layer consists of a *base calculus*, such as resolution or superposition. It must be presentable as an inference system and a redundancy criterion, as required by the saturation framework, and it must be refutationally complete.

From a base calculus, our framework can be used to derive the second layer, which we call the *splitting calculus* (Sect. 3). This extends the base calculus with splitting and inherits the base’s completeness. It works on A-clauses or A-formulas of the form $C \mathbin {\leftarrow }A$, where *C* is a base clause or formula and *A* is a set of propositional literals, called assertions (Sect. 2).

Using the saturation framework, we can prove the dynamic completeness of an abstract prover, formulated as a transition system, that implements the splitting calculus. However, this ignores a major component of AVATAR: the SAT solver. AVATAR considers only inferences involving A-formulas whose assertions are true in the current propositional model. The role of the third layer is to reflect this behavior. A *model-guided prover* operates on states of the form $(\mathcal {J}{,}\> {{\mathscr {N}}}),$ where $\mathcal {J}$ is a propositional model and ${{\mathscr {N}}}$ is a set of A-formulas (Sect. 4). This layer is also dynamically complete.

The fourth layer introduces AVATAR’s *locking* mechanism (Sect. 5). With locking, an A-formula $D \mathbin {\leftarrow }B$ can be temporarily disabled by another A-formula $C \mathbin {\leftarrow }A$ if *C* subsumes *D*,  even if $A \not \subseteq B.$ Here we make a first discovery: AVATAR-style locking compromises completeness and must be curtailed.

Finally, the fifth layer is an *AVATAR-based prover* (Sect. 6). This refines the locking model-guided prover of the fourth layer with the given clause procedure, which saturates an A-formula set by distinguishing between active and passive A-formulas. Here we make another discovery: Selecting A-formulas fairly is not enough to guarantee completeness. We need a stronger criterion.

There are also implications for other architectures. In a hypothetical tête-à-tête with the designers of labeled splitting, they might gently point out that by pioneering the use of a propositional model, including locking, they almost invented AVATAR themselves. Likewise, developers of satisfiability modulo theories (SMT) solvers might be tempted to claim that Voronkov merely reinvented SMT. To investigate such questions, we apply our framework to splitting without backtracking, labeled splitting, and SMT with quantifiers (Sect. 7). This gives us a solid basis for comparison as well as some new theoretical results.

A shorter version of this article was presented at CADE-28 [14]. This article extends the conference paper with more explanations, examples, counterexamples, and proofs. We strengthened the definition of consequence relation to require compactness, which allowed us to simplify property (D4). The property (D4) from the conference paper is proved as Lemma 5. The definition of strongly finitary was also changed to include a stronger condition on the introduced assertions, which is needed for the proof of Lemma 72.

## Preliminaries

Our framework is parameterized by abstract notions of formulas, consequence relations, inferences, and redundancy. We largely follow the conventions of Waldmann et al. [29]. A-formulas generalize Voronkov’s A-clauses [28].

### Formulas

A set ${\textbf{F}}$ of *formulas*, ranged over by $C, D \in {\textbf{F}},$ is a set that contains a distinguished element $\bot$ denoting falsehood. A *consequence relation* $\models$ over ${\textbf{F}}$ is a relation ${\models } \subseteq {({\mathscr {P}}({\textbf{F}}))}^2$ with the following properties for all sets $M, M', N, N' \subseteq {\textbf{F}}$ and formulas $C, D \in {\textbf{F}}$:

1. $$\{\bot \} \models \emptyset $$;
2. $$\{C\} \models \{C\}$$;
3. if $M' \subseteq M$ and $N' \subseteq N,$ then $M' \models N'$ implies $M \models N$;
4. if $M \models N \cup \{C\}$ and $M' \cup \{C\} \models N'$, then $M \cup M' \models N \cup N'$;
5. if $M \models N,$ then there exist finite sets $M' \subseteq M$ and $N' \subseteq N$ such that .

The intended interpretation of $M \models N$ is conjunctive on the left but disjunctive on the right: “.” The disjunctive interpretation of *N* will be useful to define splittability abstractly in Sect. 3.1. Property (D4) is called the cut rule, and (D5) is called compactness.

For their saturation framework, Waldmann et al. instead consider a fully conjunctive version of the consequence relation, with different properties. The incompatibility can easily be repaired: Given a consequence relation $\models ,$ we can obtain a consequence relation ${\models }'$ in their sense by defining $M \mathrel {{\models }'} N$ if and only if $M \models \{C\}$ for every $C \in N.$ The two versions differ only when the right-hand side is not a singleton. The conjunctive version ${\models }'$ can then be used when interacting with the saturation framework.

The $\models$ notation can be extended to allow negation on either side. Let ${\textbf{F}}_{\!{{\sim }}}$ be defined as  such that ${{\sim }{{\sim }C}} = C.$ Given $M, N \subseteq {\textbf{F}}_{\!{{\sim }}},$ we set $M \models N$ if and only ifWe write $M \models \!\!\!|N$ for the conjunction of $M \models N$ and $N \models M.$

#### Lemma 1

Let $$C\in {\textbf{F}}_{\!{{\sim }}}$$. Then $$\{C\}\cup \{{{\sim }C}\}\models \{\bot \}$$.

#### Proof

This holds by (D2) and (D3) due to the definition of $\models$ on ${\textbf{F}}_{\!{{\sim }}}$. $\square$

Following the saturation framework [29, p. 318], we distinguish between the consequence relation ${\models }$ used for stating refutational completeness and the consequence relation ${|\!\!\!\approx }$ used for stating soundness. For example, ${\models }$ could be entailment for first-order logic with equality, whereas ${|\!\!\!\approx }$ could also draw on linear arithmetic, or interpret Skolem symbols so as to make skolemization sound. Normally ${\models } \subseteq {|\!\!\!\approx }$, but this is not required.

#### Example 1

In clausal first-order logic with equality, as implemented in superposition provers, the formulas in ${\textbf{F}}$ consist of clauses over a signature ${\Sigma }.$ Each clause *C* is a finite multiset of literals $L_1, \dotsc , L_n$ written $C = L_1 \vee \cdots \vee L_n.$ The clause’s variables are implicitly quantified universally. Each literal *L* is either an atom or its negation ($\lnot$), and each atom is an unoriented equation $s \approx t$. We define the consequence relation $\models$ by letting $M \models N$ if and only if every first-order ${\Sigma }$-interpretation that satisfies all clauses in *M* also satisfies at least one clause in *N*.

### Calculi and Derivations

A refutational calculus combines a set of inferences, which are a priori mandatory, and a redundancy criterion, which identifies inferences that a posteriori need not be performed as well as formulas that can be deleted.

Let ${\textbf{F}}$ be a set of formulas equipped with $\bot .$ An ${\textbf{F}}$-*inference* $\iota$ is a tuple $(C_n,\dotsc ,C_1,D) \in {\textbf{F}}^{n+1}.$ The formulas $C_n,\dotsc ,C_1$ are the *premises*, and *D* is the *conclusion*. Define $\textit{prems}(\iota ) = \{C_n,\dotsc ,C_1\}$ and $\textit{concl}(\iota ) = \{D\}.$ An inference $\iota$ is *sound* w.r.t. $|\!\!\!\approx$ if $\textit{prems}(\iota ) |\!\!\!\approx \textit{concl}(\iota ).$ An *inference system* $\textit{Inf}{}$ is a set of ${\textbf{F}}$-inferences.

Given $N \subseteq {\textbf{F}},$ we let $\textit{Inf}{}(N)$ denote the set of all inferences in $\textit{Inf}{}$ whose premises are included in *N*,  and $\textit{Inf}{}(N, M) = \textit{Inf}{}(N {\cup }M) \setminus \textit{Inf}{}(N \setminus M)$ for the set of all inferences in $\textit{Inf}{}$ such that one or more premises are in *M* and the remaining premises are in *N*.

A *redundancy criterion* for an inference system $\textit{Inf}{}$ and a consequence relation ${\models }$ is a pair $\textit{Red}= (\textit{Red}_\text {I},\textit{Red}_\text {F}),$ where $\textit{Red}_\text {I}: {\mathscr {P}}({\textbf{F}}) \rightarrow {\mathscr {P}}(\textit{Inf}{})$ and $\textit{Red}_\text {F}:{\mathscr {P}}({\textbf{F}}) \rightarrow {\mathscr {P}}({\textbf{F}})$ enjoy the following properties for all sets $M, N \subseteq {\textbf{F}}$:

1. if $$N \models \{\bot \},$$ then $$N {\setminus } \textit{Red}_\text {F}(N) \models \{\bot \}$$;
2. if $M \subseteq N,$ then $\textit{Red}_\text {F}(M) \subseteq \textit{Red}_\text {F}(N)$ and $\textit{Red}_\text {I}(M) \subseteq \textit{Red}_\text {I}(N)$;
3. if $M \subseteq \textit{Red}_\text {F}(N),$ then $\textit{Red}_\text {F}(N) \subseteq \textit{Red}_\text {F}(N {\setminus } M)$ and $\textit{Red}_\text {I}(N) \subseteq \textit{Red}_\text {I}(N {\setminus } M)$;
4. if $\iota \in \textit{Inf}{}$ and $\textit{concl}(\iota ) \in N,$ then $\iota \in \textit{Red}_\text {I}(N).$

Inferences in $\textit{Red}_\text {I}(N)$ and formulas in $\textit{Red}_\text {F}(N)$ are said to be *redundant* w.r.t. *N*. $\textit{Red}_\text {I}$ indicates which inferences need not be performed, whereas $\textit{Red}_\text {F}$ justifies the deletion of formulas deemed useless. The above properties make the passage from static to dynamic completeness possible: (R1) ensures that deleting a redundant formula preserves a set’s inconsistency, so as not to lose refutations; (R2) and (R3) ensure that arbitrary formulas can be added and redundant formulas can be deleted by the prover; and (R4) ensures that adding an inference’s conclusion to the formula set makes the inference redundant.

A pair $(\textit{Inf}{}, \textit{Red})$ forms a *calculus*. A set $N \subseteq {\textbf{F}}$ is *saturated* w.r.t. $\textit{Inf}{}$ and $\textit{Red}_\text {I}$ if $\textit{Inf}{}(N) \subseteq \textit{Red}_\text {I}(N).$ The calculus $(\textit{Inf}{}, \textit{Red})$ is *statically* (*refutationally*) *complete* (w.r.t. $\models$) if for every set $N \subseteq {\textbf{F}}$ that is saturated w.r.t. $\textit{Inf}{}$ and $\textit{Red}_\text {I}$ and such that $N \models \{\bot \},$ we have $\bot \in N.$

#### Lemma 2

Assume that the calculus $(\textit{Inf}{},\textit{Red})$ is statically complete. Then $\bot \notin \textit{Red}_\text {F}(N)$ for every $N \subseteq {\textbf{F}}.$

#### Proof

By (R2), it suffices to show $\bot \notin \textit{Red}_\text {F}({\textbf{F}}).$ Clearly, by (D2) and (D3), ${\textbf{F}}\models \{\bot \}.$ Thus, by (R1), ${\textbf{F}}{\setminus } \textit{Red}_\text {F}({\textbf{F}}) \models \{\bot \}.$ Moreover, by (R3) and (R4), ${\textbf{F}}{\setminus } \textit{Red}_\text {F}({\textbf{F}})$ is saturated. Hence, since $(\textit{Inf}{}, \textit{Red})$ is statically complete, $\bot \in {\textbf{F}}\setminus \textit{Red}_\text {F}({\textbf{F}}).$ Therefore, $\bot \notin \textit{Red}_\text {F}({\textbf{F}}).$ $\square$

#### Remark 3

Given a redundancy criterion $(\textit{Red}_\text {I}, \textit{Red}_\text {F}),$ where $\bot \notin \textit{Red}_\text {F}({\textbf{F}}),$ we can make it stricter as follows. Define $\textit{Red}_\text {I}'$ such that $\iota \in \textit{Red}_\text {I}'(N)$ if and only if either $\iota \in \textit{Red}_\text {I}(N)$ or $\bot \in N.$ Define $\textit{Red}_\text {F}'$ such that $C \in \textit{Red}_\text {F}'(N)$ if and only if either $C \in \textit{Red}_\text {F}(N)$ or else both $\bot \in N$ and $C \not = \bot .$ Obviously, $\textit{Red}' = (\textit{Red}_\text {I}', \textit{Red}_\text {F}')$ is a redundancy criterion. Moreover, if *N* is saturated w.r.t. $\textit{Inf}{}$ and $\textit{Red}_\text {I},$ then *N* is saturated w.r.t. $\textit{Inf}{}$ and $\textit{Red}_\text {I}'$, and if the calculus $(\textit{Inf}{}, \textit{Red})$ is statically complete, then $(\textit{Inf}{}, \textit{Red}')$ is also statically complete. (In the last case, the condition $\bot \notin \textit{Red}_\text {F}({\textbf{F}})$ holds by Lemma 2.)

A *sequence* $(x_i)_i$ over a set *X* is a function from ${\mathbb {N}}$ to *X* that maps each $i \in {\mathbb {N}}$ to $x_i \in X.$ Let $(X_i)_i$ be a sequence of sets. Its *limit inferior* is $X_\infty = \textstyle \liminf _{j\rightarrow \infty } X_j = \bigcup _i \bigcap _{j \ge i} X_j,$ and its *limit superior* is $X^\infty = \textstyle \limsup _{j\rightarrow \infty } X_j = \bigcap _i \bigcup _{j \ge i} X_j.$ The elements of $X_\infty$ are called *persistent*. A sequence $(N_i)_i$ of sets of ${\textbf{F}}$-formulas is *weakly fair* w.r.t. $\textit{Inf}{}$ and $\textit{Red}_\text {I}$ if $\textit{Inf}{}(N_\infty ) \subseteq \bigcup _i \textit{Red}_\text {I}(N_i)$ and *strongly fair* if ${\limsup _{i\rightarrow \infty } \textit{Inf}{}(N_i)} \subseteq \bigcup _i \textit{Red}_\text {I}(N_i).$ Weak fairness requires that all inferences possible from some index *i* and ever after eventually be performed or become redundant for another reason. Strong fairness requires the same from all inferences that are possible infinitely often, even if not continuously so. Both can be used to ensure that some limit is saturated.

Given a relation ${\rhd } \subseteq X^2$ (pronounced “triangle”), a $\rhd$-*derivation* is a sequence of *X* elements such that $x_i \rhd x_{i+1}$ for every *i*. Finite runs can be extended to derivations by repeating the final state infinitely. We must then ensure that $\rhd$ supports such stuttering. Abusing language, and departing slightly from the saturation framework, we will say that a derivation $(x_i)_i$ *terminates* if $x_i = x_{i+1} = \cdots$ for some index *i*.

Let ${\rhd _{\!\textit{Red}_\text {F}}} \subseteq ({\mathscr {P}}({\textbf{F}}))^2$ be the relation such that $M \rhd _{\!\textit{Red}_\text {F}}N$ if and only if $M {\setminus } N \subseteq \textit{Red}_\text {F}(N).$ Note that it is reflexive and hence supports stuttering. The relation is also transitive due to (R3). We could additionally require soundness ($M |\!\!\!\approx N$) or at least consistency preservation ($M \not |\!\!\!\approx \{\bot \}$ implies $N \not |\!\!\!\approx \{\bot \}$), but this is unnecessary for proving completeness.

The calculus $(\textit{Inf}{}, \textit{Red})$ is *dynamically* (*refutationally*) *complete* (w.r.t. $\models$) if for every $\rhd _{\!\textit{Red}_\text {F}}$-derivation $(N_i)_i$ that is weakly fair w.r.t. $\textit{Inf}{}$ and $\textit{Red}_\text {I}$ and such that $N_0 \models \{\bot \},$ we have $\bot \in N_i$ for some *i*.

### A-Formulas

We fix throughout a countable set ${{\textbf {V}}}$ of *propositional variables* $\textsf{v}_0,\textsf{v}_1,\dots .$ For each $\textsf{v} \in {{\textbf {V}}},$ let $\lnot \textsf{v} \in \lnot {{\textbf {V}}}$ denote its negation, with $\lnot \lnot \textsf{v} = \textsf{v}.$ We assume that a formula $\textit{fml}(\textsf{v}) \in {\textbf{F}}$ is associated with each propositional variable $\textsf{v} \in {{\textbf {V}}}.$ Intuitively, $\textsf{v}$ approximates $\textit{fml}(\textsf{v})$ at the propositional level. This definition is extended so that $\textit{fml}(\lnot \textsf{v}) = {{\sim }\textit{fml}(\textsf{v})}.$ A propositional literal, or *assertion*, $a \in {{\textbf {A}}}= {{\textbf {V}}}{\cup }\lnot {{\textbf {V}}}$ over ${{\textbf {V}}}$ is either a propositional variable $\textsf{v}$ or its negation $\lnot \textsf{v}.$

A *propositional interpretation* $\mathcal {J} \subseteq {{\textbf {A}}}$ is a set of assertions such that for every variable $\textsf{v} \in {{\textbf {V}}}$, exactly one of $\textsf{v} \in \mathcal {J}$ and $\lnot \textsf{v} \in \mathcal {J}$ holds. We lift $\textit{fml}$ to sets in an elementwise fashion: . In the rest of this article, we will often implicitly lift functions elementwise to sets. The condition on the variables ensures that $\mathcal {J}$ is propositionally consistent. $\mathcal {J}$ might nevertheless be inconsistent for $\models$, which takes into account the semantics of the formulas $\textit{fml}(\textsf{v})$ associated with the variables $\textsf{v}$; for example, we might have $\mathcal {J} = \{\textsf{v}_0, \textsf{v}_1\}$, $\textit{fml}(\textsf{v}_0) = \textsf{p}(x)$, and $\textit{fml}(\textsf{v}_1) = \lnot \textsf{p}(\textsf{a})$, and $\mathcal {J} \models \{\bot \}.$

An *A-formula* over a set ${\textbf{F}}$ of *base formulas* and an assertion set ${{\textbf {A}}}$ is a pair ${{\mathscr {C}}} = (C, A) \in {\textbf {AF}}= {\textbf{F}}\times {{\mathscr {P}}}_\text {fin}({{\textbf {A}}}),$ written $C \mathbin {\leftarrow }A,$ where *C* is a formula and *A* is a finite set of assertions $\{a_1, \dotsc , a_n\}$ understood as an implication . We identify $C \mathbin {\leftarrow }\emptyset$ with *C* and define the projections $\lfloor C \mathbin {\leftarrow }A \rfloor = C$ and $\lfloor (C_n \mathbin {\leftarrow }A_n, \dots , C_0 \mathbin {\leftarrow }A_0) \rfloor = (C_n, \dots , C_0)$. Moreover, ${{\mathscr {N}}}_\bot$ is the set consisting of all A-formulas of the form $\bot \mathbin {\leftarrow }A \in {{\mathscr {N}}}$, where $A \in {{\mathscr {P}}}_\text {fin}({{\textbf {A}}}).$ Since $\bot \mathbin {\leftarrow }\{a_1,\dotsc ,a_n\}$ can be read as $\lnot a_1 \vee \dots \vee \lnot a_n,$ we call such A-formulas *propositional clauses*. (In contrast, we call a variable-free base formula such as $\textsf{p} \vee \textsf{q}$ a ground clause when ${\textbf{F}}$ is first-order logic.) The set ${{\mathscr {N}}}_\bot$ represents the clauses considered by the SAT solver in the original AVATAR [28]. Note the use of calligraphic letters (e.g., ${{\mathscr {C}}}, {{\mathscr {N}}}$) to range over A-formulas and sets of A-formulas.

Model-guided provers only consider A-formulas whose assertions are true in the current interpretation. Thus we say that an A-formula $C \mathbin {\leftarrow }A \in {\textbf {AF}}$ is *enabled* in a propositional interpretation $\mathcal {J}$ if $A \subseteq \mathcal {J}$. A set of A-formulas is *enabled* in $\mathcal {J}$ if all of its members are enabled in $\mathcal {J}.$ Given an A-formula set ${{\mathscr {N}}} \subseteq {\textbf {AF}},$ the *enabled projection* ${{\mathscr {N}}}_{\mathcal {J}} \subseteq \lfloor \mathscr {N} \rfloor$ consists of the projections $\lfloor \mathscr {C} \rfloor$ of all A-formulas $\mathscr {C}$ enabled in $\mathcal {J}.$ Analogously, the *enabled projection* $\textit{Inf}{}_{\mathcal {J}} \subseteq \lfloor \textit{Inf}{} \rfloor$ of a set $\textit{Inf}{}$ of ${\textbf {AF}}$-inferences consists of the projections $\lfloor \iota \rfloor$ of all inferences $\iota \in \textit{Inf}{}$ whose premises are all enabled in $\mathcal {J}.$

A propositional interpretation $\mathcal {J}$ is a *propositional model* of ${{\mathscr {N}}}_\bot ,$ written $\mathcal {J} \models {{\mathscr {N}}}_\bot ,$ if $\bot \notin ({{\mathscr {N}}}_\bot )_{\mathcal {J}}$. (i.e., $({{\mathscr {N}}}_\bot )_{\mathcal {J}} = \emptyset$). Moreover, we write $\mathcal {J} |\!\!\!\approx {{\mathscr {N}}}_\bot$ if $\bot \notin ({{\mathscr {N}}}_\bot )_{\mathcal {J}}$ or $\textit{fml}(\mathcal {J}) |\!\!\!\approx \{\bot \}$. A set ${{\mathscr {N}}}_\bot$ is *propositionally satisfiable* if there exists an interpretation $\mathcal {J}$ such that $\mathcal {J} \models {{\mathscr {N}}}_\bot$. In contrast to consequence relations, propositional modelhood $\models$ interprets the set ${{\mathscr {N}}}_\bot$ conjunctively: $\mathcal J \models \mathscr {N}_\bot$ is informally understood as $\mathcal {J} \models \bigwedge {{\mathscr {N}}}_\bot .$

Given consequence relations $\models$ and $|\!\!\!\approx$, we lift them from ${\mathscr {P}}({\textbf{F}})$ to ${\mathscr {P}}({\textbf {AF}})$: ${{\mathscr {M}}} \models {{\mathscr {N}}}$ if and only if ${{\mathscr {M}}}_{\mathcal {J}} \models \lfloor {{\mathscr {N}}} \rfloor$ for every $\mathcal {J}$ in which $\mathscr {N}$ is enabled, and ${{\mathscr {M}}} |\!\!\!\approx {{\mathscr {N}}}$ if and only if $\textit{fml}(\mathcal {J}) {\cup }{{\mathscr {M}}}_{\mathcal {J}} |\!\!\!\approx \lfloor {{\mathscr {N}}} \rfloor$ for every $\mathcal {J}$ in which $\mathscr {N}$ is enabled. The consequence relation $\models$ is used for the completeness of the splitting prover and only captures what inferences such a prover must perform. In contrast, $|\!\!\!\approx$ captures a stronger semantics: For example, thanks to $\textit{fml}(\mathcal {J})$ among the premises for $|\!\!\!\approx ,$ the A-formula $\textit{fml}(a) \mathbin {\leftarrow }\{a\}$ is always a $|\!\!\!\approx$-tautology. Also note that assuming $\emptyset \not \models \emptyset$, then ${\models } \subseteq {|\!\!\!\approx }$ on sets that contain exclusively propositional clauses. When needed, we use $|\!\!\!\approx _{\textbf{F}}$ to denote $|\!\!\!\approx$ on ${\mathscr {P}}({\textbf{F}})$ and analogously for $|\!\!\!\approx _{\textbf {AF}}$, as well as $\models _{\textbf{F}}$ and $\models _{\textbf {AF}}$.

#### Lemma 4

The relations $\models$ and $|\!\!\!\approx$ on ${\mathscr {P}}({\textbf {AF}})$ are consequence relations.

#### Proof

We consider only ${|\!\!\!\approx }$; the proof for $\models$ is analogous. For (D1), we need to show $\textit{fml}(\mathcal {J}) \cup \{\bot \} |\!\!\!\approx \emptyset$ for every $\mathcal {J}$ because $\emptyset$ is always enabled. This follows from (D1) and (D3). For (D2), we need to show $\textit{fml}(\mathcal {J}) \cup \{C \mathbin {\leftarrow }A\}_{\mathcal {J}} |\!\!\!\approx \lfloor \{C \mathbin {\leftarrow }A\} \rfloor ,$ assuming $C \mathbin {\leftarrow }A$ is enabled in $\mathcal {J}.$ Hence it suffices to show $\textit{fml}(\mathcal {J}) \cup \{C\} |\!\!\!\approx \{C\},$ which follows from (D2) and (D3). For (D3), it suffices to show $\textit{fml}(\mathcal {J}) \cup \mathscr {M}_{\mathcal {J}}|\!\!\!\approx \lfloor \mathscr {N} \rfloor$ assuming that $\mathscr {N}$ is enabled in $\mathcal {J},$ and $\textit{fml}(\mathcal {J}) \cup \mathscr {M}'_{\mathcal {J}} |\!\!\!\approx \lfloor \mathscr {N}' \rfloor$ for every $\mathscr {M}' \subseteq \mathscr {M}$ and $\mathscr {N}' \subseteq \mathscr {N}.$ This follows from (D3) and monotonicity of $\lfloor \, \rfloor$ and $(\,)_{\mathcal {J}}$. For (D4), we need to show $\textit{fml}(\mathcal J) \cup (\mathscr {M} \cup \mathscr {M}') _{\mathcal J} |\!\!\!\approx \lfloor \mathscr {N} \cup \mathscr {N}' \rfloor ,$ assuming $\textit{fml}(\mathcal J) \cup \mathscr {M} _{\mathcal J} |\!\!\!\approx \lfloor \mathscr {N} \rfloor \cup \{C\}$ if $C \mathbin {\leftarrow }A$ is enabled in $\mathcal J,$ $\textit{fml}(\mathcal J) \cup \mathscr {M}' _{\mathcal J} \cup \{C \mathbin {\leftarrow }A\} _{\mathcal J} |\!\!\!\approx \lfloor \mathscr {N}' \rfloor ,$ and $\mathscr {N} \cup \mathscr {N}'$ is enabled in $\mathcal J.$ This follows directly from (D4) if $C \mathbin {\leftarrow }A$ is enabled in $\mathcal J,$ and from (D3) if $C \mathbin {\leftarrow }A$ is not enabled.

Finally, we show the compactness of $|\!\!\!\approx _{\textbf {AF}}$ (D5), using the compactness of propositional logic. First we consider the case where $\mathscr {N}$ is never enabled. Then the set of assertions in $\mathscr {N}$, seen as conjunctions of propositional literals, is unsatisfiable. By compactness, there exists a finite subset of these assertions that is also unsatisfiable, i.e., there is a finite subset $\mathscr {N}'$ of $\mathscr {N}$ that is also never enabled. Thus for any finite subset $\mathscr {M}'$ of $\mathscr {M}$, $\mathscr {M}'|\!\!\!\approx \mathscr {N}'$ as wanted.

Otherwise, there is at least one $\mathcal J$ enabling $\mathscr {N}.$ By abuse of notation, we write $\mathscr {N}_A$ even if $A \subseteq {{\textbf {A}}}$ is not an interpretation. For every interpretation $\mathcal J$ in which $\mathscr {N}$ is enabled, there exist by compactness of ${|\!\!\!\approx _{\textbf{F}}}$ finite sets $\mathcal J' \subseteq \mathcal J,$ $\mathscr {M}^\mathcal J \subseteq \mathscr {M},$ and $\mathscr {N}^\mathcal J \subseteq \mathscr {N}$ such that $\textit{fml}(\mathcal J') \cup {\mathscr {M}^{\mathcal J}} _{\mathcal J'} |\!\!\!\approx \lfloor {\mathscr {N}^{\mathcal J}} \rfloor .$ Define  Note that $\mathcal J \models E$ if and only if $\mathscr {N}$ is enabled in $\mathcal J.$ This observation implies that the sets of propositional clauses *E* and $\{ \bot \mathbin {\leftarrow }\mathcal J' \mid \mathcal J\,\text {interpretation where}\,\mathscr {N}\,\text { is enabled} \} \cup E$ are, respectively, propositionally satisfiable and propositionally unsatisfiable. By compactness, there exists a finite unsatisfiable subset $\{ \bot \mathbin {\leftarrow }\mathcal J_1', \dots , \bot \mathbin {\leftarrow }\mathcal J_n' \} \cup E'$ of the latter set.

Let $\mathscr {M}' = \bigcup _i \mathscr {M}^{\mathcal J_i}$ and $\mathscr {N}' = \bigcup _i \mathscr {N}^{\mathcal J_i} \cup \mathscr {N}''$ where $\mathcal J_i$ is any of the interpretations enabling $\mathscr {N}$ that is at the origin of the existence of this $\mathcal J_i'$ and $\mathscr {N}''$ is a finite subset of $\mathscr {N}$ such that all assertions in $E'$ also occur negated in $\mathscr {N}''.$ Note that both $\mathscr {M}'$ and $\mathscr {N}'$ are finite sets. It now suffices to show $\mathscr {M}' |\!\!\!\approx \mathscr {N}'.$ Thus let $\mathcal J$ be an interpretation in which $\mathscr {N}'$ is enabled. Then $\mathcal J \models E'$ because all assertions in $E'$ also appear negated in $\mathscr {N}'' \subseteq \mathscr {N}'.$ Thus, since $\{ \bot \mathbin {\leftarrow }\mathcal J_1', \dots , \bot \mathbin {\leftarrow }\mathcal J_n' \} \cup E'$ is unsatisfiable, there must exist an index *k* such that $\mathcal J \not \models \bot \mathbin {\leftarrow }\mathcal J_k'$, that is, $\mathcal J_k' \subseteq \mathcal J$. We have $\textit{fml}(\mathcal J_k') \cup {\mathscr {M} ^ {\mathcal J_k}} _{\mathcal J_k'} |\!\!\!\approx \lfloor \mathscr {N} ^ {\mathcal J_k} \rfloor$ by construction, and thus $\smash {\textit{fml}(\mathcal J) \cup \bigcup _i {\mathscr {M} ^ {\mathcal J_i}} _{\mathcal J} |\!\!\!\approx \bigcup _i \lfloor \mathscr {N} ^ {\mathcal J_i} \rfloor \cup \lfloor \mathscr {N}'' \rfloor }$ by (D3). $\square$

Given sets $M, N \subseteq {\mathscr {P}}({\textbf{F}}),$ the expression $M \models N$ can refer to either the base consequence relation on ${\mathscr {P}}({\textbf{F}})$ or the lifted consequence relation on ${\mathscr {P}}({\textbf {AF}})$ (since ${\textbf{F}}\subseteq {\textbf {AF}}$). Fortunately, there is no ambiguity. First, let us show a preparatory lemma:

#### Lemma 5

Let $\models$ be a consequence relation on ${\textbf{F}},$ and $M, N \subseteq {\textbf{F}}.$ If $M' \models N'$ for all $M' \supseteq M$ and $N' \supseteq N$ such that $M' \cup N' = {\textbf{F}}$, then $M \models N.$

#### Proof

By contraposition, we assume that $M \not \models N$, and we need to find $M' \supseteq M$ and $N' \supseteq N$ such that $M' \cup N' = {\textbf{F}}$ and $M' \not \models N'.$ We apply Zorn’s lemma to obtain a maximal element $(M', N')$ of the set  with the order $(M_1,N_1) \le (M_2,N_2)$ if and only if $M_1 \subseteq M_2$ and $N_1 \subseteq N_2$. Compactness of $\models$, together with (D3), guarantees that every chain in this set has an upper bound; for nonempty chains, this is the pairwise union of all the elements in the chain. It remains to show that $M' \cup N'= {\textbf{F}}.$ Assume to the contrary that $C \not \in M' \cup N'$ for some *C*. Due to the maximality of $(M',N')$, we necessarily have $M' \cup \{C\} \models N'$ and $M' \models N' \cup \{C\}.$ Applying the cut rule for ${\models },$ we get $M' \models N',$ a contradiction. $\square$

#### Lemma 6

The two versions of $\models$ coincide on ${\textbf{F}}$-formulas, and similarly for $|\!\!\!\approx$.

#### Proof

The first property is obvious. For the second property, the argument is as follows. Let $M, N \subseteq {\textbf{F}}$. Then we must show that $M |\!\!\!\approx _{\textbf{F}}N$ if and only if $M |\!\!\!\approx _{\textbf {AF}}N$. First assume that $M |\!\!\!\approx _{\textbf{F}}N.$ Then clearly $\textit{fml}(\mathcal {J}) \cup M |\!\!\!\approx _{\textbf{F}}N$ for any $\mathcal {J}$ by (D3) and thus $M |\!\!\!\approx _{\textbf {AF}}N.$ Assuming $M |\!\!\!\approx _{\textbf {AF}}N,$ we show $M |\!\!\!\approx _{\textbf{F}}N$ using Lemma 5. It thus suffices to show that $M' |\!\!\!\approx _{\textbf{F}}N'$ for every $M' \supseteq M$ and $N' \supseteq N$ such that $M' \cup N' = {\textbf{F}}.$ Set  Then $\textit{fml}(\mathcal J) \cup M \cup {{\sim }N} \subseteq M' \cup {{\sim }N'}.$ By the assumption $M |\!\!\!\approx _{\textbf {AF}}N$ we have $\textit{fml}(\mathcal {J}) \cup M |\!\!\!\approx _{\textbf{F}}N$ and thus $M' |\!\!\!\approx _{\textbf{F}}N'$ via (D3). $\square$

Aside from resolving ambiguity, Lemma 6 justifies the use of splitting in provers without compromising soundness or completeness: When we prove a completeness theorem that claims that a given prover derives $\bot$ from any initial $\models _{\textbf {AF}}$-unsatisfiable set $M \subseteq {\textbf {AF}}$, Lemma 6 allows us to conclude that it also derives $\bot$ when starting from any initial $\models _{\textbf{F}}$-unsatisfiable set $M \subseteq {\textbf{F}}$.

Given a formula $C \in {\textbf{F}}_{\!{{\sim }}},$ let $\textit{asn}(C)$ denote the set of assertions $a \in {{\textbf {A}}}$ such that $\{\textit{fml}(a)\} |\!\!\!\approx \!\!\!|\{C\}.$ Normally, we would make sure that $\textit{asn}(C)$ is nonempty for every formula *C*. Given $a \in \textit{asn}(C),$ observe that if $a \in \textit{asn}(D),$ then $\{C\} |\!\!\!\approx \!\!\!|\{D\},$ and if $\lnot a \in \textit{asn}(D),$ then $\{C\} |\!\!\!\approx \!\!\!|\{{{\sim }D\}}.$

#### Remark 7

Our propositional interpretations are always total. We could also consider partial interpretations—that is, $\mathcal J \subseteq {{\textbf {A}}}$ such that at most one of $\textsf{v} \in \mathcal J$ and $\lnot \textsf{v} \in \mathcal J$ holds for every $\textsf{v} \in V$. But this is not necessary, because partial interpretations can be simulated by total ones: For every variable $\textsf{v}$ in the partial interpretation, we can use two variables $\textsf{v}^+$ and $\textsf{v}^-$ in the total interpretation and interpret $\textsf{v}^+$ as true if $\textsf{v}$ is true and $\textsf{v}^-$ as true if $\textsf{v}$ is false. By adding the propositional clause $\bot \mathbin {\leftarrow }\{\textsf{v}^-, \textsf{v}^+\}$, every total model of the translated A-formulas corresponds to a partial model of the original A-formulas.

#### Example 8

In the original description of AVATAR [28], the connection between first-order clauses and assertions takes the form of a function $[\,]: {\textbf{F}}\rightarrow {{\textbf {A}}}.$ The encoding is such that $[\lnot C] = \lnot [C]$ for every ground unit clause *C* and $[C] = [D]$ if and only if *C* is syntactically equal to *D* up to variable renaming. This can be supported in our framework by letting $\textit{fml}(\textsf{v}) = C$ for some *C* such that $[C] = \textsf{v}$, for every propositional variable $\textsf{v}.$

A different encoding is used to exploit the theories of an SMT solver [4]. With a notion of $|\!\!\!\approx$-entailment that gives a suitable meaning to Skolem symbols, we can go further and have $[\lnot C(\textsf{sk}_{\lnot C(x)})] = \lnot [C(x)].$ Even if the superposition prover considers $\textsf{sk}_{\lnot C(x)}$ an uninterpreted symbol (according to $\models$), the SAT or SMT solver can safely prune the search space by assuming that *C*(*x*) and $\lnot C(\textsf{sk}_{\lnot C(x)})$ are exhaustive (according to $|\!\!\!\approx$).

## Splitting Calculi

Let ${\textbf{F}}$ be a set of base formulas equipped with $\bot ,$ $\models ,$ and ${|\!\!\!\approx }.$ The consequence relation $|\!\!\!\approx$ is assumed to be nontrivial: (D6) $\emptyset \not |\!\!\!\approx \emptyset .$ Let ${{\textbf {A}}}$ be a set of assertions over ${{\textbf {V}}}$, and let ${\textbf {AF}}$ be the set of A-formulas over ${\textbf{F}}$ and ${{\textbf {A}}}.$ Let $(\textit{FInf}{},\textit{FRed})$ be a base calculus for ${\textbf{F}}$-formulas, where $\textit{FRed}$ is a redundancy criterion that additionally satisfies

(R5) $$\textit{Inf}{}({\textbf{F}}, \textit{Red}_\text {F}(N)) \subseteq \textit{Red}_\text {I}(N)$$ for every $$N \subseteq {\textbf{F}}$$;
(R6) $$\bot \notin \textit{FRed}_\text {F}(N)$$ for every $$N \subseteq {\textbf{F}}$$;
(R7) $$C \in \textit{FRed}_\text {F}(\{\bot \})$$ for every $$C \not = \bot .$$

These requirements can easily be met by a well-designed redundancy criterion. Requirement (R5) is called *reducedness* by Waldmann et al. [30, Sect. 2.3]. Requirement (R6) must hold of any complete calculus (Lemma 2), and (R7) can be made without loss of generality (Remark 3). Bachmair and Ganzinger’s redundancy criterion for superposition [1, Sect. 4.3] meets (R1)–(R7).

From a base calculus, we will define an induced *splitting calculus* $(\textit{SInf}{},\textit{SRed})$. We will show that the splitting calculus is sound w.r.t. $|\!\!\!\approx$ and that it is statically and dynamically complete w.r.t. ${\models }.$ Furthermore, we will show two stronger results that take into account the switching of propositional models that characterizes most splitting architectures: strong static completeness and strong dynamic completeness.

### The Inference Rules

We start with the mandatory inference rules.

#### Definition 9

The *splitting inference system* $\textit{SInf}{}$ consists of all instances of the following two rules:
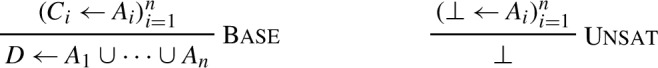


For Base, the side condition is $(C_n,\dotsc ,C_1,D) \in \textit{FInf}{}.$ For Unsat, the side condition is that $\{\bot \mathbin {\leftarrow }A_1,\dotsc ,\bot \mathbin {\leftarrow }A_n\}$ is propositionally unsatisfiable.

In addition, the following optional inference rules can be used if desired; the completeness proof does not depend on their application. Rules identified by double bars, such as Split, are simplifications; they replace their premises with their conclusions in the current A-formula set. The premises’ removal is justified by $\textit{SRed}_\text {F},$ defined in Sect. 3.2.
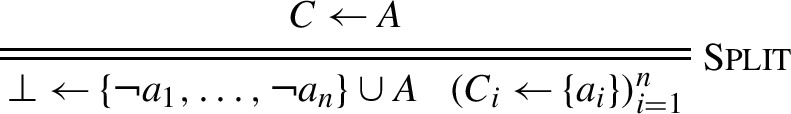


In the Split rule, we require that $C \ne \bot$ is splittable into $C_1,\dotsc ,C_n$ and that $a_i \in \textit{asn}(C_i)$ for each *i*. A-formula *C* is *splittable* into formulas $C_1,\dotsc ,C_n$ if $n \ge 2$, $\{C\} |\!\!\!\approx \{C_1,\dotsc ,C_n\}$ and $C \in \textit{FRed}_\text {F}(\{C_i\})$ for each *i*.

Split performs an *n*-way case analysis on *C*. Each case $C_i$ is approximated by an assertion $a_i.$ The first conclusion expresses that the cases are exhaustive. The *n* other conclusions assume $C_i$ if its approximation $a_i$ is true.

In a clausal prover, typically $C = C_1 \vee \cdots \vee C_n,$ where the subclauses $C_i$ have mutually disjoint sets of variables and form a maximal split. For example, the clause $\textsf{p}(x) \vee \textsf{q}(x)$ is not splittable because of the shared variable *x*, whereas $\textsf{p}(x) \vee \textsf{q}(y)$ can be split into $\{\textsf{p}(x){,}\;\textsf{q}(y)\}$.
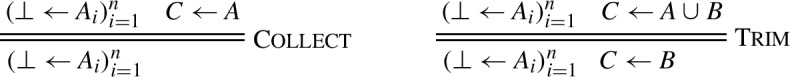


For Collect, we require $C \not = \bot$ and $\{\bot \mathbin {\leftarrow }A_i\}_{i=1}^n |\!\!\!\approx \{\bot \mathbin {\leftarrow }A\}.$ For Trim, we require $C \not = \bot$ and $\{\bot \mathbin {\leftarrow }A_i\}_{i=1}^n \cup \{\bot \mathbin {\leftarrow }A\} |\!\!\!\approx \{\bot \mathbin {\leftarrow }B\}.$

Collect removes A-formulas whose assertions cannot be satisfied by any model of the propositional clauses—a form of garbage collection. Similarly, Trim removes assertions that are entailed by existing propositional clauses.
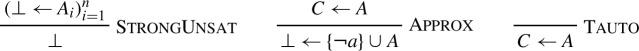


For StrongUnsat, we require $\{\bot \mathbin {\leftarrow }A_i\}_{i=1}^n |\!\!\!\approx \{\bot \}.$ For Approx, we require $a \in \textit{asn}(C).$ For Tauto, we require ${|\!\!\!\approx }\; \{C \mathbin {\leftarrow }A\}.$

StrongUnsat is a variant of Unsat that uses $|\!\!\!\approx$ instead of ${\models }.$ A splitting prover may choose to apply StrongUnsat if desired, but only Unsat is necessary for completeness. In practice, $|\!\!\!\approx$-entailment can be much more expensive to decide, or even be undecidable. A splitting prover could invoke an SMT solver [4] ($|\!\!\!\approx$) with a time limit, falling back on a SAT solver ($\models$) if necessary.

Approx can be used to make any derived A-formula visible to $|\!\!\!\approx$. It is similar to a one-way split. Tauto, which asserts a $|\!\!\!\approx$-tautology, allows communication in the other direction, from the SMT or SAT solver to the calculus.

#### Example 10

Suppose the base calculus is first-order resolution [2] and the initial clauses are $\lnot \textsf{p}(\textsf{a}),$ $\lnot \textsf{q}(z, z),$ and $\textsf{p}(x)\vee \textsf{q}(y, \textsf{b}),$ as in Sect. 1. Split replaces the last clause by $\bot \mathbin {\leftarrow }\{\lnot \textsf{v}_0, \lnot \textsf{v}_1\},$ $\textsf{p}(x)\mathbin {\leftarrow }\{\textsf{v}_0\},$ and $\textsf{q}(y, \textsf{b})\mathbin {\leftarrow }\{\textsf{v}_1\}.$ Two Base inferences then generate $\bot \mathbin {\leftarrow }\{\textsf{v}_0\}$ and $\bot \mathbin {\leftarrow }\{\textsf{v}_1\}.$ Finally, Unsat generates $\bot .$

#### Example 11

Consider a splitting calculus obeying the AVATAR conventions of Example 8. When splitting on $C(x) \vee D(y),$ after closing the *C*(*x*) case, we can assume that *C*(*x*) does not hold when considering the *D*(*y*) case. This can be achieved by adding the A-clause $\lnot C(\textsf{sk}_{\lnot C(x)}) \mathbin {\leftarrow }\{\lnot [C(x)]\}$ using Tauto. If we use an SMT solver that is strong enough to determine that $\lnot C(\textsf{sk}_{\lnot C(x)})$ and *D*(*y*) are inconsistent, we can then apply StrongUnsat immediately, skipping the *D*(*y*) branch altogether. This would be the case if we took $C(x):= \textsf{f}(x) \mathbin {>} 0$ and $D(y):= \textsf{f}(y) \mathbin {>} 3$ with a solver that supports linear arithmetic and quantifiers. We are not aware of any prover that implements this idea, although a similar idea is described for ground *C*(*x*) in the context of labeled splitting [15, Sect. 2].

#### Example 12

Consider a splitting calculus whose propositional solver is an SMT solver supporting linear arithmetic. Suppose that we are given the inconsistent clause set $\{\textsf{c} > 0{,}\; \textsf{c} < 0\}$. Two applications of Approx make these clauses visible to the SMT solver, as the propositional clause set $\{\bot \mathbin {\leftarrow }\lnot (\textsf{c} > 0){,}\; \bot \mathbin {\leftarrow }\lnot (\textsf{c} < 0)\}$. Then the SMT solver, modeled by StrongUnsat, detects the unsatisfiability.

The splitting inference system commutes nicely with the enabled projection:

#### Lemma 13

($$\textit{SInf}{}(\mathscr {N}) )_{\mathcal J} = \textit{FInf}{}(\mathscr {N} _{\mathcal J})$$ if $$\bot \not \in \mathscr {N} _{\mathcal J}.$$

#### Proof

The condition $\bot \not \in \mathscr {N} _{\mathcal J}$ rules out the Unsat inferences. It remains to show that the enabled projection of a Base inference is an $\textit{FInf}{}$-inference from enabled premises, and vice versa. $\square$

#### Theorem 14

**(Soundness)** The rules Unsat, Split, Collect, Trim, StrongUnsat, Approx, and Tauto are sound w.r.t. ${|\!\!\!\approx }.$ Moreover, if every rule in $\textit{FInf}{}$ is sound w.r.t. $|\!\!\!\approx$ (on ${\mathscr {P}}({\textbf{F}})$), then the rule Base is sound w.r.t. $|\!\!\!\approx$ (on ${\mathscr {P}}({\textbf {AF}})$).

#### Proof

Cases Unsat, StrongUnsat, Tauto:Trivial.

Case Split:For the left conclusion, by definition of $|\!\!\!\approx ,$ it suffices to show $\textit{fml}(\mathcal {J}) {\cup }\{C\} |\!\!\!\approx \{\bot \}$ for every $\mathcal {J} \supseteq A {\cup }\{\lnot a_1, \dotsc , \lnot a_n\}.$ By the side condition $\{C\} |\!\!\!\approx \{C_1,\dotsc ,C_n\},$ it suffices in turn to show $\textit{fml}(\mathcal {J}) {\cup }\{C_i\} |\!\!\!\approx \{\bot \}$ for every *i*. Notice that ${{\sim }C_i} \in \textit{fml}(\mathcal {J}).$ The entailment amounts to $\bigl (\textit{fml}(\mathcal {J}) {\setminus } \{{{\sim }C_i}\}\bigr ) {\cup }\{C_i\} |\!\!\!\approx \{C_i\},$ which follows from (D2) and (D3).

For the right conclusions, we must show $\textit{fml}(\mathcal {J}) {\cup }\lfloor \{C \mathbin {\leftarrow }A\}_{\mathcal {J}} \rfloor |\!\!\!\approx \{C_i\}$ for every $\mathcal {J} \supseteq \{a_i\}.$ Notice that $C_i \in \textit{fml}(\mathcal {J}).$ The desired result follows from (D2) and (D3).

Case Collect:We must show $\{\bot \mathbin {\leftarrow }A_i\}_{i=1}^n |\!\!\!\approx \{C \mathbin {\leftarrow }A\}.$ This follows from the stronger side condition $\{\bot \mathbin {\leftarrow }A_i\}_{i=1}^n |\!\!\!\approx \{\bot \mathbin {\leftarrow }A\}.$

Case Trim:Only the right conclusion is nontrivial. Let ${{\mathscr {N}}} = \{\bot \mathbin {\leftarrow }A_i\}_{i=1}^n.$ It suffices to show ${{\mathscr {N}}}_{\mathcal {J}} {\cup }\{C \mathbin {\leftarrow }A\}_{\mathcal {J}} |\!\!\!\approx \{C\}$ for every $\mathcal {J} \supseteq B.$ Assume $\mathcal {J} |\!\!\!\approx {{\mathscr {N}}}_{\mathcal {J}} {\cup }\{C \mathbin {\leftarrow }A\}_{\mathcal {J}}.$ By the side condition ${{\mathscr {N}}} \cup \{\bot \mathbin {\leftarrow }A\} |\!\!\!\approx \{\bot \mathbin {\leftarrow }B\},$ we get ${{\mathscr {N}}}_{\mathcal {J}} \cup \{\bot \mathbin {\leftarrow }A\}_{\mathcal {J}} |\!\!\!\approx \{\bot \}$, meaning that either ${{\mathscr {N}}}_{\mathcal {J}} |\!\!\!\approx \{\bot \}$ or $\mathcal J \supseteq A$. The first case is trivial. In the other case, $\mathcal {J} |\!\!\!\approx {{\mathscr {N}}}_{\mathcal {J}} {\cup }\{C\}$ and thus $\mathcal {J} |\!\!\!\approx \{C\},$ as required.

Case Approx:The proof is as for the left conclusion of Split.

Case Base:To show $\{C_i \mathbin {\leftarrow }A_i\}_{i=1}^n |\!\!\!\approx \{D \mathbin {\leftarrow }A_1 \cup \cdots \cup A_n\},$ by the definition of $|\!\!\!\approx$ on ${\mathscr {P}}({\textbf {AF}})$, it suffices to show $\{C_1,\dotsc ,C_n\} |\!\!\!\approx \{D\}.$ This follows from the soundness of the inferences in $\textit{FInf}{}.$ $\square$

### The Redundancy Criterion

Next, we lift the base redundancy criterion.

#### Definition 15

The *splitting redundancy criterion* $\textit{SRed}= (\textit{SRed}_\text {I}, \textit{SRed}_\text {F})$ is specified as follows. An A-formula $C \mathbin {\leftarrow }A \in {\textbf {AF}}$ is redundant w.r.t. ${{\mathscr {N}}}$, written $C \mathbin {\leftarrow }A \in \textit{SRed}_\text {F}({{\mathscr {N}}}),$ if either of these conditions is met:

1. $C \in \textit{FRed}_\text {F}({{\mathscr {N}}}_{\mathcal {J}})$ for every propositional interpretation $\mathcal {J} \supseteq A$; or
2. there exists an A-formula $C \mathbin {\leftarrow }B \in {{\mathscr {N}}}$ such that $B \subset A.$

An inference $\iota \in \textit{SInf}{}$ is redundant w.r.t. ${{\mathscr {N}}}$, written $\iota \in \textit{SRed}_\text {I}({{\mathscr {N}}}),$ if either of these conditions is met:

(3) $\iota$ is a Base inference and $\{\iota \}_{\mathcal {J}} \subseteq \textit{FRed}_\text {I}({{{\mathscr {N}}}_{\mathcal {J}}})$ for every $\mathcal {J}$; or
(4) $\iota$ is an Unsat inference and $\bot \in {{\mathscr {N}}}.$

Condition (1) lifts $\textit{FRed}_\text {F}$ to A-formulas. It is used both as such and to justify the Split and Collect rules, as we will see below. Condition (2) is used to justify Trim. We will use $\textit{SRed}_\text {F}$ to justify global A-formula deletion, but also $\textit{FRed}_\text {F}$ for local A-formula deletion in the locking prover. Note that $\textit{SRed}$ is not reduced. Inference redundancy partly commutes with the enabled projection:

#### Lemma 16

$$(\textit{SRed}_\text {I}(\mathscr {N})) _{\mathcal J} \subseteq \textit{FRed}_\text {I}(\mathscr {N} _{\mathcal J})$$ if $$\bot \not \in \mathscr {N}.$$

#### Proof

Since $\bot \not \in \mathscr {N},$ condition (4) of the definition of $\textit{SRed}_\text {I}$ cannot apply. The inclusion then follows directly from condition (3) applied to the interpretation $\mathcal J.$ $\square$

#### Lemma 17

$$\bot \notin \textit{SRed}_\text {F}({{\mathscr {N}}})$$ for every $${{\mathscr {N}}} \subseteq {\textbf {AF}}.$$

#### Proof

By Lemma 2, condition (1) of the definition of $\textit{SRed}_\text {F}$ cannot apply. Nor can condition (2). $\square$

#### Lemma 18

$\textit{SRed}$ is a redundancy criterion.

#### Proof

We will first show that the restriction $\textit{ARed}$ of $\textit{SRed}$ to Base inferences is a redundancy criterion. Then we will consider Unsat inferences.

We start by showing that $\textit{ARed}$ is a special case of the redundancy criterion $\textit{FRed}^{\cap {{\mathscr {G}}},\sqsupset }$ of Waldmann et al. [29, Sect. 3]—the *intersection of lifted redundancy criteria with tiebreaker orders*. Then we can simply invoke Theorem 37 and Lemma 19 from their technical report [30].

To strengthen the redundancy criterion, we define a *tiebreaker order* $\sqsupset$ such that $C \mathbin {\leftarrow }A \sqsupset D \mathbin {\leftarrow }B$ if and only if $C = D$ and $A \subset B.$ In this way, $C \mathbin {\leftarrow }B$ is redundant w.r.t. $C \mathbin {\leftarrow }A$ if $A \subset B$, even though the base clause is the same. The only requirement on $\sqsupset$ is that it must be well founded, which is the case since the assertion sets of A-formulas are finite. We also define a family of *grounding functions* ${{\mathscr {G}}}_{\mathcal {J}}$ indexed by a propositional model $\mathcal {J}.$ Here, “grounding” will mean enabled projection. For A-formulas ${{\mathscr {C}}},$ we set ${{\mathscr {G}}}_{\mathcal {J}}({{\mathscr {C}}}) = {\{{{\mathscr {C}}}\}_{\mathcal {J}}}.$ For inferences $\iota ,$ we set ${{\mathscr {G}}}_{\mathcal {J}}(\iota ) = {\{\iota \}_{\mathcal {J}}}.$

We must show that ${{\mathscr {G}}}_{\mathcal {J}}$ satisfies the following characteristic properties of grounding function: (G1) ${{\mathscr {G}}}_{\mathcal {J}}(\bot ) = \{\bot \}$; (G2) for every ${{\mathscr {C}}} \in {\textbf {AF}},$ if $\bot \in {{\mathscr {G}}}_{\mathcal {J}}({{\mathscr {C}}}),$ then ${{\mathscr {C}}} = \bot$; and (G3) for every $\iota \in \textit{SInf}{},$ ${{\mathscr {G}}}_{\mathcal {J}}(\iota ) \subseteq \textit{FRed}_\text {I}({{\mathscr {G}}}_{\mathcal {J}}(\textit{concl}(\iota ))).$

Condition (G1) obviously holds, and (G3) holds by property (R4) of $\textit{FRed}.$ However, (G2) does not hold, a counterexample being $\bot \mathbin {\leftarrow }\{a\}.$ On closer inspection, Waldmann et al. use (G2) only to prove static completeness (Theorems 27 and  45 in their technical report) but not to establish that $\textit{FRed}^{\cap {{\mathscr {G}}},\sqsupset }$ is a redundancy criterion, so we can proceed. It is a routine exercise to check that $\textit{ARed}$ coincides with $\textit{FRed}^{\cap {{\mathscr {G}}},\sqsupset } = (\textit{FRed}_\text {I}^{\cap {{\mathscr {G}}}},\textit{FRed}_\text {F}^{\cap {{\mathscr {G}}},\sqsupset }),$ which is defined as follows:

1. $\iota \in \textit{FRed}_\text {I}^{\cap {{\mathscr {G}}}}({{\mathscr {N}}})$ if and only if for every propositional interpretation $\mathcal {J},$ we have ${{\mathscr {G}}}_{\mathcal {J}}(\iota ) \subseteq \textit{FRed}_\text {I}({{\mathscr {G}}}_{\mathcal {J}}({{\mathscr {N}}}))$;
2. ${{\mathscr {C}}} \in \textit{FRed}_\text {F}^{\cap {{\mathscr {G}}},\sqsupset }({{\mathscr {N}}})$ if and only if for every propositional interpretation $\mathcal {J}$ and every ${{\mathscr {D}}} \in {{\mathscr {G}}}_{\mathcal {J}}({{\mathscr {C}}}),$ either ${{\mathscr {D}}} \mathbin {\in } \textit{FRed}_\text {F}({{\mathscr {G}}}_{\mathcal {J}}({{\mathscr {N}}}))$ or there exists ${{\mathscr {C}}}' \in {{\mathscr {N}}}$ such that ${{\mathscr {C}}}' \sqsubset {{\mathscr {C}}}$ and ${{\mathscr {D}}} \in {{\mathscr {G}}}_{\mathcal {J}}({{\mathscr {C}}}').$

We also need to check that the consequence relation $\models$ used in $\textit{SRed}$ coincides with the consequence relation $\models ^{\cap }_{\mathscr {G}}$, which is defined as $\mathscr {M} \models ^{\cap }_{\mathscr {G}} \{\mathscr {C}\}$ if and only if for every $\mathcal J$ and $D \in \mathscr {G}_\mathcal J(\{\mathscr {C}\})$, we have $\mathscr {G}_\mathcal J(\mathscr {M}) \models \{ D \}$. After expanding $\mathscr {G}_\mathcal J$, this is exactly the definition we used for lifting $\models$ to ${\textbf {AF}}$.

To extend the above result to $\textit{SRed},$ we must show the second half of conditions (R2) and (R3) as well as (R4) for Unsat inferences.

(R2)Given an Unsat inference $\iota ,$ we must show that if ${{\mathscr {M}}} \subseteq {{\mathscr {N}}}$ and $\iota \in \textit{SRed}_\text {I}({{\mathscr {M}}}),$ then $\iota \in \textit{SRed}_\text {I}({{\mathscr {N}}}).$ This holds because if $\bot \in {{\mathscr {M}}},$ then $\bot \in {{\mathscr {N}}}.$

(R3)Given an Unsat inference $\iota ,$ we must show that if ${{\mathscr {M}}} \subseteq \textit{SRed}_\text {F}({{\mathscr {N}}})$ and $\iota \in \textit{SRed}_\text {I}({{\mathscr {N}}}),$ then $\iota \in \textit{SRed}_\text {I}({{\mathscr {N}}} {\setminus } {{\mathscr {M}}}).$ This amounts to proving that if $\bot \in {{\mathscr {N}}},$ then $\bot \in {{\mathscr {N}}} {\setminus } {{\mathscr {M}}},$ which follows from Lemma 17.

(R4)Given an Unsat inference $\iota ,$ we must show that if $\bot \in {{\mathscr {N}}},$ then $\iota \in \textit{SRed}_\text {I}({{\mathscr {N}}}).$ This follows from the definition of $\textit{SRed}_\text {I}.$ $\square$

$\textit{SRed}$ is highly versatile. It can justify the deletion of A-formulas that are propositionally tautological, such as $C \mathbin {\leftarrow }\{\textsf{v}, \lnot \textsf{v}\}$. It lifts the base redundancy criterion gracefully: If $D \in \textit{FRed}_\text {F}(\{C_i\}_{i=1}^n),$ then $D \mathbin {\leftarrow }A_1 \cup \cdots \cup A_n \in \textit{SRed}_\text {F}(\{C_i \mathbin {\leftarrow }A_i\}_{i=1}^n)$. It also allows other simplifications, as long as the assertions on A-formulas used to simplify a given $C \mathbin {\leftarrow }A$ are contained in *A*. If the base criterion $\textit{FRed}_\text {F}$ supports subsumption (e.g., following the lines of Waldmann et al. [29]), this also extends to A-formulas: $D \mathbin {\leftarrow }B \in \textit{SRed}_\text {F}(\{C \mathbin {\leftarrow }A\})$ if *D* is strictly subsumed by *C* and $B \supseteq A$, or if $C = D$ and $B \supset A.$ Finally, it is strong enough to justify case splits and the other simplification rules presented in Sect. 3.1.

#### Theorem 19

**(Simplification)**  For every Split, Collect, or Trim inference, the conclusions collectively make the premises redundant according to $\textit{SRed}_\text {F}.$

#### Proof

Case Split:We must show $C \mathbin {\leftarrow }A \in \textit{SRed}_\text {F}( \{\bot \mathbin {\leftarrow }\{\lnot a_1, \dotsc , \lnot a_n\} \cup A\} {\cup }\{C_i \mathbin {\leftarrow }\{a_i\}\}_{i=1}^n ).$ By condition (1) of the definition of $\textit{SRed}_\text {F},$ it suffices to show $C \in \textit{FRed}_\text {F}( \{\bot \mathbin {\leftarrow }\{\lnot a_1, \dotsc , \lnot a_n\}\}_{\mathcal {J}}{\cup }(\{C_i \mathbin {\leftarrow }\{a_i\}\}_{i=1}^n)_{\mathcal {J}}$ for every $\mathcal {J} \supseteq A.$ If $a_i \in \mathcal {J}$ for some *i*,  this follows from Split’s side condition $C \in \textit{FRed}_\text {F}(\{C_i\}).$ Otherwise, this follows from (R7), the requirement that $C \in \textit{FRed}_\text {F}(\{\bot \})$, since $C \not = \bot .$

Case Collect:We must show $C \mathbin {\leftarrow }A \in \textit{SRed}_\text {F}( \{\bot \mathbin {\leftarrow }A_i\}_{i=1}^n ).$ By condition (1) of the definition of $\textit{SRed}_\text {F},$ it suffices to show $C \in \textit{FRed}_\text {F}( {(\{\bot \mathbin {\leftarrow }A_i\}_{i=1}^n)_{\mathcal {J}}} )$ for every $\mathcal {J} \supseteq A.$ If $A_i \subseteq \mathcal {J}$ for some *i*,  this follows from Collect’s side condition that $C \not = \bot$ and (R7). Otherwise, from the side condition $\{\bot \mathbin {\leftarrow }A_i\}_{i=1}^n |\!\!\!\approx \{\bot \mathbin {\leftarrow }A\},$ we obtain $\emptyset |\!\!\!\approx \{\bot \},$ which contradicts (D6).

Case Trim:We must show $C \mathbin {\leftarrow }A \cup B \in \textit{SRed}_\text {F}( \{\bot \mathbin {\leftarrow }A_i\}_{i=1}^n \cup \{C \mathbin {\leftarrow }B\} ).$ This follows directly from condition (2) of the definition of $\textit{SRed}_\text {F}.$ $\square$

Annoyingly, the redundancy criterion $\textit{SRed}$ does not mesh well with $\alpha$-equivalence. We would expect the A-formula $\textsf{p}(x) \mathbin {\leftarrow }\{a\}$ to be subsumed by $\textsf{p}(y) \mathbin {\leftarrow }\emptyset ,$ where *x*, *y* are variables, but this is not covered by condition (2) of $\textit{SRed}_\text {F}$ because $\textsf{p}(x) \not = \textsf{p}(y).$ The simplest solution is to take ${\textbf{F}}$ to be the quotient of some set of raw formulas by $\alpha$-equivalence. An alternative is to generalize the theory so that the projection operator ${\mathscr {G}_{\mathcal {J}}}$ generates entire $\alpha$-equivalence classes (e.g., ${\mathscr {G}_{\mathcal {J}} (\{\textsf{p}(x)\})} = \{\textsf{p}(x){,}\> \textsf{p}(y){,}\> \textsf{p}(z){,}\> \cdots \}$) or groundings (e.g., ${\mathscr {G}_{\mathcal {J}}(\{\textsf{p}(x)\})} = \{\textsf{p}(\textsf{a}){,}\> \textsf{p}(\textsf{f}(\textsf{a})){,}\> \cdots \}$). Waldmann et al. describe the second approach [29, Sect. 4].

### Standard Saturation

We will now prove that the splitting calculus is statically complete and therefore dynamically complete. Unfortunately, derivations produced by most practical splitting architectures violate the fairness condition associated with dynamic completeness. Nevertheless, the standard completeness notions are useful stepping stones, so we start with them.

#### Lemma 20

Let ${{\mathscr {N}}} \subseteq {\textbf {AF}}$ be an A-formula set, and let $\mathcal {J}$ be a propositional interpretation. If ${{\mathscr {N}}}$ is saturated w.r.t. $\textit{SInf}{}$ and $\textit{SRed}_\text {I},$ then ${{{\mathscr {N}}}_{\mathcal {J}}}$ is saturated w.r.t. $\textit{FInf}{}$ and $\textit{FRed}_\text {I}.$

#### Proof

Assuming $\iota \in \textit{FInf}{}({{{\mathscr {N}}}_{\mathcal {J}}}),$ we must show $\iota \in \textit{FRed}_\text {I}({{{\mathscr {N}}}_{\mathcal {J}}}).$ The argument follows that of the “folklore” Lemma 26 in the technical report of Waldmann et al. [30]. First note that any inference in $\textit{FInf}{}$ is lifted, via Base, in $\textit{SInf}{},$ so that we have $\iota \in (\textit{SInf}{}({{\mathscr {N}}}))_{\mathcal {J}}.$ This means that there exists a Base inference $\iota _0 \in \textit{SInf}{}({{\mathscr {N}}}).$ By saturation of ${{\mathscr {N}}},$ we have $\iota _0 \in \textit{SRed}_\text {I}({{\mathscr {N}}}).$ By definition of $\textit{SRed}_\text {I},$ ${\{\iota _0\}_{\mathcal {J}}} = \{\iota \} \subseteq \textit{FRed}_\text {I}({{{\mathscr {N}}}_{\mathcal {J}})},$ as required. $\square$

#### Theorem 21

**(Static completeness)**  Assume $(\textit{FInf}{},\textit{FRed})$ is statically complete.  Then $(\textit{SInf}{},\textit{SRed})$ is statically complete.

#### Proof

Suppose ${{\mathscr {N}}} \subseteq {\textbf {AF}},$ ${{\mathscr {N}}} \models \{\bot \},$ and ${{\mathscr {N}}}$ is saturated w.r.t. $\textit{SInf}{}$ and $\textit{SRed}_\text {I}.$ We will show $\bot \in {{\mathscr {N}}}.$

First, we show $\bot \in {{{\mathscr {N}}}_{\mathcal {J}}}$ for every $\mathcal {J}.$ From ${{\mathscr {N}}} \models \{\bot \},$ by the definition of $\models$ on A-formulas, it follows that ${N_{\mathcal {J}}} \models \{\bot \}.$ Moreover, by Lemma 20, ${N_{\mathcal {J}}}$ is saturated w.r.t. $\textit{FInf}{}$ and $\textit{FRed}_\text {I}.$ By static completeness of $(\textit{FInf}{}, \textit{FRed}),$ we get $\bot \in {{{\mathscr {N}}}_{\mathcal {J}}}.$

Hence $\mathscr {N}_\bot$ is propositionally unsatisfiable. By compactness of propositional logic, there exists a finite subset $\mathscr {M}\subseteq {\mathscr {N}}$ such that $\mathscr {M}$ is propositionally unsatisfiable. By saturation w.r.t. Unsat, we obtain $\bot \in {{\mathscr {N}}},$ as required. $\square$

Thanks to the requirements on the redundancy criterion, we obtain dynamic completeness as a corollary:

#### Corollary 22

**(Dynamic completeness)**  Assume $(\textit{FInf}{},\textit{FRed})$ is statically complete. Then $(\textit{SInf}{},\textit{SRed})$ is dynamically complete.

#### Proof

This immediately follows from Theorem 21 by Lemma 6 in the technical report of Waldmann et al. [30]. $\square$

### Local Saturation

The above completeness result, about $\rhd _{\!\textit{SRed}_\text {F}}$-derivations, can be extended to prover designs based on the given clause procedure, such as the Otter, DISCOUNT, and Zipperposition loops, as explained by Waldmann et al. [29, Sect. 4]. But it fails to capture a crucial aspect of most splitting architectures. Since $\rhd _{\!\textit{SRed}_\text {F}}$-derivations have no notion of current split branch or propositional model, they place no restrictions on which inferences may be performed when.

To fully capture splitting, we need to start with a weaker notion of saturation. If an A-formula set is consistent, it should suffice to saturate w.r.t. a single propositional model. In other words, if no A-formula $\bot \mathbin {\leftarrow }A$ such that $A \subseteq \mathcal {J}$ is derivable for some model $\mathcal {J} \models {{\mathscr {N}}}_\bot ,$ the prover will never be able to apply the Unsat rule to derive $\bot .$ It should then be allowed to deliver a verdict of “consistent.” We will call such model-specific saturations *local* and standard saturations *global*.

#### Definition 23

A set ${{\mathscr {N}}} \subseteq {\textbf {AF}}$ is *locally saturated* w.r.t. $\textit{SInf}{}$ and $\textit{SRed}_\text {I}$ if either $\bot \in {{\mathscr {N}}}$ or there exists a propositional model $\mathcal {J} \models {{\mathscr {N}}}_\bot$ such that ${{{\mathscr {N}}}_{\mathcal {J}}}$ is saturated w.r.t. $\textit{FInf}{}$ and $\textit{FRed}_\text {I}.$

Local saturation works in tandem with *strong static completeness*:

#### Theorem 24

**(Strong static completeness)**  Assume $(\textit{FInf}{},\textit{FRed})$ is statically complete. Given a set ${{\mathscr {N}}} \subseteq {\textbf {AF}}$ that is locally saturated w.r.t. $\textit{SInf}{}$ and $\textit{SRed}_\text {I}$ and such that ${{\mathscr {N}}} \models \{\bot \},$ we have $\bot \in {{\mathscr {N}}}.$

#### Proof

We show $\bot \in {{\mathscr {N}}}$ by case analysis on the condition by which ${{\mathscr {N}}}$ is locally saturated. The first case is vacuous. Otherwise, let $\mathcal {J} \models {{\mathscr {N}}}_\bot .$ Since ${{\mathscr {N}}} \models \{\bot \},$ we have ${{{\mathscr {N}}}_{\mathcal {J}}} \models \{\bot \}.$ By the definition of local saturation and static completeness of $(\textit{FInf}{}, \textit{FRed}),$ we get $\bot \in {{{\mathscr {N}}}_{\mathcal {J}}},$ contradicting $\mathcal {J} \models {{\mathscr {N}}}_\bot .$ $\square$

#### Example 25

Consider the following A-clause set expressed using AVATAR conventions:

$$\begin{aligned}\{ \bot \mathbin {\leftarrow }\{\lnot [\textsf{p}(x)], \lnot [\textsf{q}(y)]\}{,}\qquad \textsf{p}(x) \mathbin {\leftarrow }\{[\textsf{p}(x)]\}{,}\qquad \textsf{q}(y) \mathbin {\leftarrow }\{[\textsf{q}(y)]\}{,}\qquad \lnot \textsf{q}(\textsf{a}) \}\end{aligned}$$

It is not globally saturated for resolution, because the conclusion $\bot \mathbin {\leftarrow }\{[\textsf{q}(y)]\}$ of resolving the last two A-clauses is missing, but it is locally saturated with $\mathcal {J} \supseteq \{[\textsf{p}(x)], \lnot [\textsf{q}(y)]\}$ as the witness in Definition 23.

We also need a notion of local fairness that works in tandem with local saturation.

#### Definition 26

A sequence $({{\mathscr {N}}}_i)_i$ of sets of A-formulas is *locally fair* w.r.t. $\textit{SInf}{}$ and $\textit{SRed}_\text {I}$ if either $\bot \in {{\mathscr {N}}}_i$ for some *i* or there exists a propositional model $\mathcal {J} \models ({{\mathscr {N}}}_\infty )_\bot$ such that $\textit{FInf}{}({(\mathscr {N}_\infty )_{\mathcal {J}}}) \subseteq \bigcup _i \textit{FRed}_\text {I}({(\mathscr {N}_i)_{\mathcal {J}}}).$

#### Lemma 27

Let $({{\mathscr {N}}}_i)_i$ be a $\rhd _{\!\textit{SRed}_\text {F}}$-derivation that is locally fair w.r.t. $\textit{SInf}{}$ and $\textit{SRed}_\text {I}.$ Then the limit inferior ${{\mathscr {N}}}_\infty$ is locally saturated w.r.t. $\textit{SInf}{}$ and $\textit{SRed}_\text {I}.$

#### Proof

The proof is by case analysis on the condition by which $({{\mathscr {N}}}_i)_i$ is locally fair. If $\bot \in \mathscr {N}_i,$ then $\bot \in \mathscr {N}_\infty$ by Lemma 17, and $\mathscr {N}_\infty$ is therefore locally saturated. In the remaining case, we have ${\mathscr {N}}_i \subseteq {\mathscr {N}}_\infty \cup \textit{SRed}_\text {F}({\mathscr {N}}_\infty )$ by Lemma 4 in the technical report of Waldmann et al. [30], and therefore $\bigcup _i \textit{FRed}_\text {I}({(\mathscr {N}_i)_{\mathcal {J}}}) \subseteq \bigcup _i \textit{FRed}_\text {I}({(\mathscr {N}_\infty )_{\mathcal {J}}} \cup \textit{FRed}_\text {F}({(\mathscr {N}_\infty )_{\mathcal {J}}})) = \bigcup _i \textit{FRed}_\text {I}({(\mathscr {N}_\infty )_{\mathcal {J}}})$ because we clearly have ${(\textit{SRed}_\text {F}(\mathscr {N}_\infty ))_{\mathcal {J}}} \subseteq \textit{FRed}_\text {F}({(\mathscr {N}_\infty )_{\mathcal {J}}}) \cup {(\mathscr {N}_\infty )_{\mathcal {J}}}.$ $\square$

Local fairness works in tandem with *strong dynamic completeness*.

#### Theorem 28

**(Strong dynamic completeness)**  Assume $(\textit{FInf}{},\textit{FRed})$ is statically complete. Given a $\rhd _{\!\textit{SRed}_\text {F}}$-derivation $({{\mathscr {N}}}_i)_i$ that is locally fair w.r.t. $\textit{SInf}{}$ and $\textit{SRed}_\text {I}$ and such that ${{\mathscr {N}}}_0 \models \{\bot \},$ we have $\bot \in {{\mathscr {N}}}_i$ for some *i*.

#### Proof

We connect the dynamic and static points of view along the lines of the proof of Lemma 6 in the technical report of Waldmann et al. [30]. First, we show that the limit inferior is inconsistent: ${{\mathscr {N}}}_\infty \models \{\bot \}.$ We have $\bigcup _i {{\mathscr {N}}}_i \supseteq {{\mathscr {N}}}_0 \models \{\bot \},$ and by (R1), it follows that $(\bigcup _i {{\mathscr {N}}}_i) {\setminus } \textit{SRed}_\text {F}(\bigcup _i {{\mathscr {N}}}_i) \models \{\bot \}.$ By their Lemma 2, $(\bigcup _i {{\mathscr {N}}}_i) {\setminus } \textit{SRed}_\text {F}(\bigcup _i {{\mathscr {N}}}_i) \subseteq {{\mathscr {N}}}_\infty .$ Hence ${{\mathscr {N}}}_\infty \supseteq (\bigcup _i {{\mathscr {N}}}_i) {\setminus } \textit{SRed}_\text {F}(\bigcup _i {{\mathscr {N}}}_i) \models \{\bot \}.$ By Lemma 27, ${{\mathscr {N}}}_\infty$ is locally saturated, so by Theorem 24, $\bot \in {{\mathscr {N}}}_\infty .$ Thus, $\bot \in {{\mathscr {N}}}_i$ for some *i*. $\square$

An alternative proof based on dynamic completeness follows:

#### Proof

We show $\bot \in {{\mathscr {N}}}_i$ for some *i* by case analysis on the condition by which $({{\mathscr {N}}}_i)_i$ is locally fair. The first case is vacuous. Otherwise, we have $\mathcal {J} \models ({{\mathscr {N}}}_\infty )_\bot .$ Since ${{\mathscr {N}}}_0 \models \{\bot \},$ we have ${({{\mathscr {N}}}_0)_{\mathcal {J}}} \models \{\bot \}.$ By the definition of local fairness and Theorem 22, we get $\bot \in {({{\mathscr {N}}}_i)_{\mathcal {J}}}$ for some *i*. By Lemma 2 and the definition of $\rhd _{\!\textit{FRed}_\text {F}},$ we obtain $\bot \in {({{\mathscr {N}}}_\infty )_{\mathcal {J}}},$ contradicting $\mathcal {J} \models ({{\mathscr {N}}}_\infty )_\bot .$ $\square$

In Sects. 4 to 6, we will review three transition systems of increasing complexity, culminating with an idealized specification of AVATAR. They will be linked by a chain of stepwise refinements, like pearls on a string. All derivations using these systems will correspond to $\rhd _{\!\textit{SRed}_\text {F}}$-derivations, and their fairness criteria will imply local fairness. Consequently, by Theorem 28, they will all be complete.

## Model-Guided Provers

The transition system $\rhd _{\!\textit{SRed}_\text {F}}$ provides a very abstract notion of splitting prover. AVATAR and other splitting architectures maintain a model of the propositional clauses, which represents the split tree’s current branch. We can capture this abstractly by refining $\rhd _{\!\textit{SRed}_\text {F}}$-derivations to incorporate a propositional model.

### The Transition Rules

The states are now pairs $(\mathcal {J}, {{\mathscr {N}}}),$ where $\mathcal {J}$ is a propositional interpretation and ${{\mathscr {N}}} \subseteq {\textbf {AF}}$. Initial states have the form $(\mathcal J, N),$ where $N \subseteq {\textbf{F}}.$ The *model-guided prover* ${{\textsf{M}}}{{\textsf{G}}}$ is defined by the following transition rules:The Derive rule can add new A-formulas (${{\mathscr {M}}}'$) and delete redundant A-formulas (${{\mathscr {M}}}$). In practice, Derive will perform only sound or consistency-preserving inferences, but we impose no such restriction. If soundness of a prover is desired, it can be derived easily from the soundness of the individual inferences. Similarly, ${{\mathscr {M}}}$ and ${{\mathscr {M}}}'$ will usually be enabled in $\mathcal J$, but we do not require this.

The interpretation $\mathcal {J}$ should be a model of ${{\mathscr {N}}}_\bot$ most of the time; when it is not, Switch can be used to switch interpretation or StrongUnsat to finish the refutation. Although the condition $\mathcal {J}_i \models ({{\mathscr {N}}}_i)_\bot$ might be violated for some *i*,  to make progress we must periodically check it and apply Switch as needed. Much of the work that is performed while the condition is violated will likely be wasted. To avoid this waste, Vampire invokes the SAT solver whenever it selects a clause as part of the given clause procedure.

Transitions can be combined to form $\Longrightarrow _{{{\textsf{M}}}{{\textsf{G}}}}$-derivations (pronounced “arrow-${{\textsf{M}}}{{\textsf{G}}}$-derivations”).

#### Lemma 29

If $$(\mathcal {J}, {{\mathscr {N}}}) \Longrightarrow _{{{\textsf{M}}}{{\textsf{G}}}}(\mathcal {J}', {{\mathscr {N}}}'),$$ then $${{\mathscr {N}}} \rhd _{\!\textit{SRed}_\text {F}}{{\mathscr {N}}}'.$$

#### Proof

The only rule that deletes A-formulas, Derive, exclusively takes out A-formulas that are redundant w.r.t. the next state, as mandated by $\rhd _{\!\textit{SRed}_\text {F}}.$ $\square$

To develop our intuitions, we will study several examples of $\Longrightarrow _{{{\textsf{M}}}{{\textsf{G}}}}$-derivations. In all the examples in this section, the base calculus is first-order resolution, and $\models$ is entailment for first-order logic with equality.

#### Example 30

Let us revisit Example 10. Initially, the propositional interpretation is $\mathcal {J}_0 = \{ \lnot \textsf{v}_0, \lnot \textsf{v}_1 \}.$ After the split, we have the A-clauses $\lnot \textsf{p}(\textsf{a}),$ $\lnot \textsf{q}(z, z),$ $\textsf{p}(x)\mathbin {\leftarrow }\{\textsf{v}_0\},$ $\textsf{q}(y, \textsf{b})\mathbin {\leftarrow }\{\textsf{v}_1\},$ and $\bot \mathbin {\leftarrow }\{\lnot \textsf{v}_0, \lnot \textsf{v}_1\}.$ The natural option is to switch interpretation. We take $\mathcal {J}_1 = \{\textsf{v}_0, \lnot \textsf{v}_1\}.$ We then derive $\bot \mathbin {\leftarrow }\{\textsf{v}_0\}.$ Since $\mathcal {J}_1 \not \models \bot \mathbin {\leftarrow }\{\textsf{v}_0\},$ we switch to $\mathcal {J}_2 = \{\lnot \textsf{v}_0, \textsf{v}_1\},$ where we derive $\bot \mathbin {\leftarrow }\{\textsf{v}_1\}.$ Finally, we detect that the propositional clauses are unsatisfiable and generate $\bot$. This corresponds to the transitions below, where arrows are annotated by transition names and light gray boxes identify enabled A-clauses:
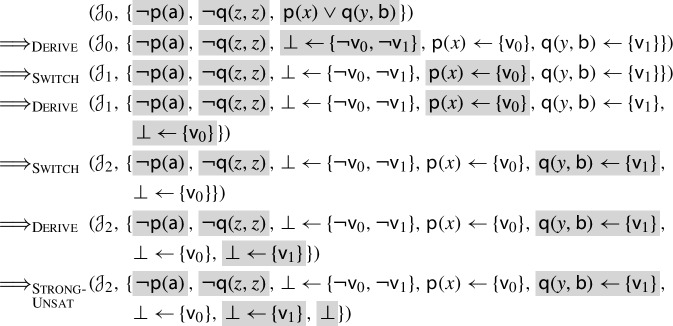


### Fairness

We need a fairness criterion for ${{\textsf{M}}}{{\textsf{G}}}$ that implies local fairness of the underlying $\rhd _{\!\textit{SRed}_\text {F}}$-derivation. The latter requires a witness $\mathcal {J}$ but gives us no hint as to where to look for one. This is where basic topology comes into play.

#### Definition 31

A propositional interpretation $\mathcal {J}$ is a *limit point* in a sequence $(\mathcal {J}_i)_i$ if there exists a subsequence $(\mathcal {J}'_i)_i$ of $(\mathcal {J}_i)_i$ such that $\mathcal {J} = \mathcal {J}'_\infty = \mathcal {J}'^\infty .$

Intuitively, a limit point is a propositional interpretation that is the limit of a family of interpretations that we revisit infinitely often. We will see that there always exists a limit point. To achieve fairness, we will focus on saturating a limit point.

#### Example 32

**Fig. 1:**
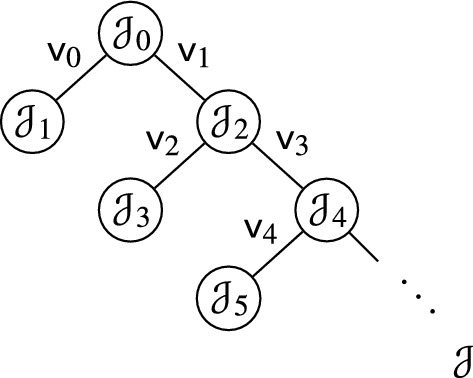
A split tree with a single infinite branch

Let $(\mathcal {J}_i)_i$ be the sequence such that $\mathcal {J}_{2i} \cap {{\textbf {V}}}= \{\textsf{v}_1,\textsf{v}_3,\ldots ,\textsf{v}_{2i-1} \}$ (i.e., $\textsf{v}_1,\textsf{v}_3,\ldots ,\textsf{v}_{2i-1}$ are true and the other variables are false) and $\mathcal {J}_{2i+1\!} = (\mathcal {J}_{2i} {\setminus } \{ \lnot \textsf{v}_{2i} \}) \cup \{\textsf{v}_{2i}\}.$ Although it is not in the sequence, the interpretation $\mathcal {J} \cap {{\textbf {V}}}= \{\textsf{v}_1, \textsf{v}_3, \ldots \}$ is a limit point. The split tree of $(\mathcal {J}_i)_i$ is depicted in Fig. 1. The direct path from the root to a node labeled $\mathcal {J}_i$ specifies the assertions that are true in $\mathcal {J}_i.$ The limit point $\mathcal {J}$ corresponds to the only infinite branch of the tree.

**Fig. 2:**
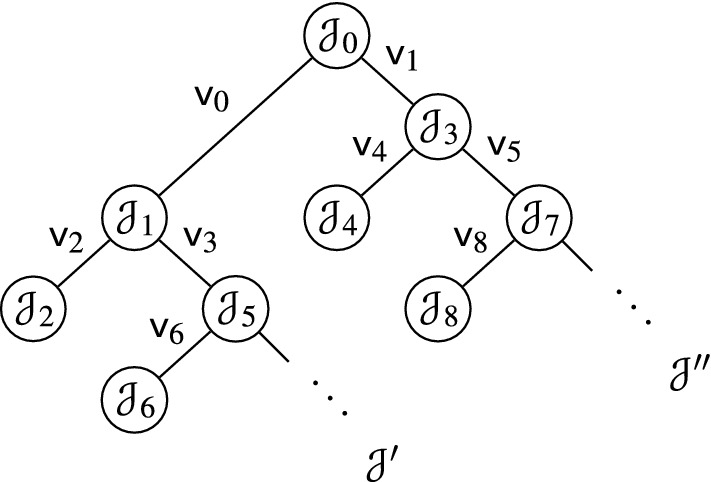
A split tree with two infinite branches

The above example hints at why the proof of ${{\textsf{M}}}{{\textsf{G}}}$’s dynamic completeness is nontrivial: Some derivations might involve infinitely many split branches, making it difficult for the prover to focus on any single one and saturate it.

#### Example 33

A sequence may have multiple limit points. Let $(\mathcal {J}_i)_i$ be the sequence such that $\mathcal {J}_0 \cap {{\textbf {V}}}= \emptyset ,$    and . This sequence has two limit points: $\mathcal {J}' = \liminf _{i\rightarrow \infty } \mathcal {J}_{4i+1}$ and $\mathcal {J}'' = \liminf _{i\rightarrow \infty } \mathcal {J}_{4i+3}.$ The split tree is depicted in Fig. 2.

#### Lemma 34

Let $(\mathcal {J}_i)_i$ be a sequence of propositional interpretations. Then $\mathcal {J}_\infty \subseteq \mathcal {J} \subseteq \mathcal {J}^\infty$ for all of its limit points $\mathcal {J}.$

#### Proof

By definition of limit point, there must exist a subsequence $(\mathcal {J}'_i)_i$ of $(\mathcal {J}_i)_i$ such that $\mathcal {J}'_\infty = \mathcal {J}'^\infty = \mathcal {J}.$ It is obvious by definition that the limit inferior of a subsequence must be a superset of the limit inferior of the original sequence, and analogously that the limit superior of a subsequence must be a subset of the limit superior of the original sequence. $\square$

#### Lemma 35

Every sequence $(\mathcal {J}_i)_i$ of propositional interpretations has at least one limit point.

#### Proof

The set of propositional interpretations is homeomorphic to the set of functions ${{\textbf {V}}}\rightarrow \{0, 1\}$ equipped with the product topology. Since ${{\textbf {V}}}$ is countable, this set of functions is a compact metric space—namely, the Cantor space. In a compact metric space, every sequence has a convergent subsequence, and thus a limit point in our notation. $\square$

We can nearly as easily supply an elementary proof:

#### Proof

We construct a subsequence $(\mathcal J'_j)_j$ converging to a limit point $\mathcal J$ in such a way that $\mathcal J'_j$ gets the first *j* variables right—i.e., such that $\mathcal J'_j \models \textsf{v}_k$ if and only if $\mathcal J \models \textsf{v}_k$ for every $k \le j.$ Moreover, we maintain the invariant that there are infinitely many elements in the sequence $(\mathcal J_i)_i$ that agree with this finite prefix. Assume that we have already defined $\mathcal J'_0, \dots , \mathcal J'_j$. Among the infinitely many elements $\mathcal J_i$ that agree with $\mathcal J'_0, \dots , \mathcal J'_j$ on $\textsf{v}_1,\dotsc \textsf{v}_j$, there must be infinitely many with $\mathcal J_i \models \textsf{v}_{j+1}$ or infinitely many with $\mathcal J_i \models \lnot \textsf{v}_{j+1}.$ In the first case, set $\mathcal J'_{j+1} = \mathcal J_i$ for one such index *i*, and analogously in the second case. $\square$

Lemma 35 tells us that every sequence has a limit point. No matter how erratically the prover switches branches, it will systematically explore at least one branch in a limit point. It then suffices to perform the base $\textit{FInf}{}$-inferences fairly in that branch:

#### Definition 36

An $\Longrightarrow _{{{\textsf{M}}}{{\textsf{G}}}}$-derivation $(\mathcal {J}_i, {{\mathscr {N}}}_i)_i$ is *fair* if either (1) $\bot \in {{\mathscr {N}}}_i$ for some *i* or (2) $\mathcal {J}_i \models ({{\mathscr {N}}}_i)_\bot$ for infinitely many indices *i* and there exists a limit point $\mathcal {J}$ of $(\mathcal {J}_i)_i$ such that $\textit{FInf}{}({(\mathscr {N}_\infty )_{\mathcal {J}}}) \subseteq \bigcup _i \textit{FRed}_\text {I}({(\mathscr {N}_i)_{\mathcal {J}}}).$

Until $\bot$ is derived, it is impossible in a fair $\Longrightarrow _{{{\textsf{M}}}{{\textsf{G}}}}$-derivation to delay Switch forever (by the first half of (2)) or to starve off Derive by performing only Switch transitions (by the second half of (2)). Also note that we make no assumptions about the order in which propositional models are enumerated; the propositional solver is given carte blanche.

We might at first expect that a realistic prover would ensure the inclusion $\textit{FInf}{}({(\mathscr {N}_\infty )_{\mathcal {J}}}) \subseteq \bigcup _i \textit{FRed}_\text {I}({(\mathscr {N}_i)_{\mathcal {J}}})$ for *all* limit points $\mathcal J$. However, a prover like Vampire, based on the given clause procedure with an age-based heuristic, might saturate only one of the limit points, as we will see in Sect. 6.2.

Fairness of $\Longrightarrow _{{{\textsf{M}}}{{\textsf{G}}}}$-derivations is deliberately defined in terms of $\textit{FRed}_\text {I}$ instead of $\textit{SRed}_\text {I}$. This results in a more suitable notion of fairness, since it allows the prover to ignore formulas and inferences that are locally redundant at the limit point but not redundant w.r.t. $(\textit{SInf}{}, \textit{SRed})$. For example, the inference $(t \approx s{,}\> \textsf{p}(t){,}\> \textsf{p}(s))$ is locally redundant in $\mathcal J \supseteq \{v_0\}$ if the A-clause $\textsf{p}(s) \mathbin {\leftarrow }\{\textsf{v}_0\}$ has already been derived, but it is not redundant w.r.t. $(\textit{SInf}{}, \textit{SRed})$.

#### Lemma 37

Let $(\mathcal {J}_i, {{\mathscr {N}}}_i)_i$ be an $\Longrightarrow _{{{\textsf{M}}}{{\textsf{G}}}}$-derivation such that $\mathcal {J}_i \models ({{\mathscr {N}}}_i)_\bot$ for infinitely many indices *i*. Then for every ${{\mathscr {D}}} \in ({{\mathscr {N}}}_\infty )_\bot ,$ there exists an index *i* such that $\mathcal {J}_{j} \models \{{{\mathscr {D}}}\}$ for every $j \ge i.$

#### Proof

Let $\mathscr {D} = \bot \mathbin {\leftarrow }A \in ({{\mathscr {N}}}_\infty )_\bot .$ Then $\bot \mathbin {\leftarrow }A \in \mathscr {N}_k$ for some *k*. For every $j \ge k$, we then have $\bot \mathbin {\leftarrow }B \in \mathscr {N}_j$ for some $B \subseteq A$, since every $\Longrightarrow _{{{\textsf{M}}}{{\textsf{G}}}}$-derivation is a $\rhd _{\!\textit{SRed}_\text {F}}$-derivation and $\bot \mathbin {\leftarrow }A$ can only become redundant due to such a $\bot \mathbin {\leftarrow }B$. Since $\{\bot \mathbin {\leftarrow }B\} \models \{\bot \mathbin {\leftarrow }A\}$, we get $(\mathscr {N}_j)_\bot \models \{\bot \mathbin {\leftarrow }A\}$ for every $j \ge k.$ By the assumption, there exists an index $i \ge k$ such that $\mathcal {J}_i \models \{\bot \mathbin {\leftarrow }A\}.$ For $j \ge i,$ the interpretation changes only in the Switch transition, which has $\mathcal {J}_j \models (\mathscr {N}_j)_\bot$ as a side condition. Since $(\mathscr {N}_j)_\bot \models \{\bot \mathbin {\leftarrow }A\},$ we have $\mathcal {J}_j \models \{\bot \mathbin {\leftarrow }A\}$ for every $j \ge i.$ $\square$

#### Lemma 38

Let $(\mathcal {J}_i, \mathscr {N}_i)_i$ be an $\Longrightarrow _{{{\textsf{M}}}{{\textsf{G}}}}$-derivation such that $\mathcal {J}_i \models (\mathscr {N}_i)_\bot$ for infinitely many indices *i*,  and let $\mathcal {J}$ be a limit point of $(\mathcal {J}_i)_i.$ Then $\mathcal {J} \models (\mathscr {N}_\infty )_\bot .$

#### Proof

Let $\mathscr {C} \in (\mathscr {N}_\infty )_\bot .$ By Lemma 37, there exists an index *i* such that $\mathcal {J}_j \models \{\mathscr {C}\}$ for every $j \ge i.$ Let $(\mathcal {J}_i')_i$ be the subsequence associated with the limit point $\mathcal {J}.$ Then there also exists an index $i'$ such that $\mathcal {J}_j' \models \{\mathscr {C}\}$ for every $j \ge i'$ and hence $\mathcal {J} \models \{\mathscr {C}\}.$ $\square$

In the spirit of refinement, we have that fairness of an $\Longrightarrow _{{{\textsf{M}}}{{\textsf{G}}}}$-derivation implies local fairness of the underlying $\rhd _{\!\textit{SRed}_\text {F}}$-derivation:

#### Theorem 39

**(Fairness)** Let $(\mathcal {J}_i, {{\mathscr {N}}}_i)_i$ be a fair $\Longrightarrow _{{{\textsf{M}}}{{\textsf{G}}}}$-derivation. Then $({{\mathscr {N}}}_i)_i$ is a $\rhd _{\!\textit{SRed}_\text {F}}$-derivation that is locally fair w.r.t. $\textit{SInf}{}$ and $\textit{SRed}_\text {I}.$

#### Proof

The case where $\bot \in {{\mathscr {N}}}_i$ for some *i* is trivial. Otherwise, we have that $\mathcal {J}_i \models ({{\mathscr {N}}}_i)_\bot$ for infinitely many *i* and there is a limit point $\mathcal {J}$ such that $\textit{FInf}{}({(\mathscr {N}_\infty )_{\mathcal {J}}}) \subseteq \bigcup _i \textit{FRed}_\text {I}({(\mathscr {N}_i)_{\mathcal {J}}}).$ We take this limit point as the witness for $\mathcal {J}$ in Definition 26. It remains to show that $\mathcal {J} \models ({{\mathscr {N}}}_\infty )_\bot$. This follows from Lemma 38. $\square$

#### Corollary 40

**(Dynamic completeness)**  Assume $(\textit{FInf}{},\textit{FRed})$ is statically complete. Given a fair $\Longrightarrow _{{{\textsf{M}}}{{\textsf{G}}}}$-derivation $(\mathcal {J}_i, {{\mathscr {N}}}_i)_i$ such that ${{\mathscr {N}}}_0 \models \{\bot \},$ we have $\bot \in {{\mathscr {N}}}_i$ for some *i*.

#### Proof

By Theorem 28. $$\square $$

A well-behaved propositional solver, as in labeled splitting, enumerates potential models in a systematic way and always gives rise to a single limit point $\mathcal {J}_\infty ,$ which can be taken for $\mathcal {J}$ in the definition of fairness (Definition 36). To achieve this kind of fairness, a splitting prover would perform all inferences from persistently enabled A-formulas—that is, A-formulas that eventually become enabled and remain enabled forever. In a prover based on the given clause procedure, this can be implemented in the standard way, using an age-based selection heuristic [27, Sect. 4]. However, such a strategy is not sufficient if the prover exploits local redundancy, as we will see in Sects. 5 and 7.2, even if the propositional solver is well behaved.

By contrast, an unconstrained solver, as supported by AVATAR, can produce multiple limit points; in particular, the restart feature of SAT solvers [20] could produce this kind of behavior. Then it is more challenging to ensure fairness, as we will see in Sect. 6.

#### Example 41

Suppose that we leave out $\lnot \textsf{q}(z, z)$ from the initial clause set of Example 10. Then we can still derive $\bot \mathbin {\leftarrow }\{\textsf{v}_0\},$ as in Example 30, but not $\bot \mathbin {\leftarrow }\{\textsf{v}_1\}.$ By static completeness of the splitting calculus, we conclude that the A-clause set is consistent.

#### Example 42

Consider the initial clause set consisting of $\textsf{p}(x) \vee \textsf{q}(\textsf{a})$ and $\lnot \textsf{q}(y) \vee \textsf{q}(\textsf{f}(y)).$ Without splitting, and without selection [2, Sect. 3], a resolution prover would diverge attempting to generate infinitely many clauses of the form $\textsf{p}(x) \vee \textsf{q}(\textsf{f}^i(\textsf{a})).$

By contrast, in a splitting prover, we might split the first clause, yielding the A-clauses $\textsf{p}(x) \mathbin {\leftarrow }\{\textsf{v}_0\},$ $\textsf{q}(\textsf{a}) \mathbin {\leftarrow }\{\textsf{v}_1\},$ and $\bot \mathbin {\leftarrow }\{\lnot \textsf{v}_0, \lnot \textsf{v}_1\}.$ If we then choose the model $\{\textsf{v}_1\}$ and commit to it, we will also diverge, although somewhat faster since we do not need to carry around the literal ${\textsf{p}(x)}.$ On the other hand, if we at any point switch to $\{\textsf{v}_0\},$ we notice that $\{\textsf{p}(x)\}$ is saturated and terminate. This illustrates the benefits of employing an unconstrained SAT solver.

#### Example 43

It is crucial to invoke the SAT solver often enough—in other words, to take Switch and StrongUnsat transitions periodically. Suppose that the inconsistent initial clause set of Example 10 is supplemented by the prolific but unhelpful clauses $\textsf{r}(\textsf{a})$ and $\lnot \textsf{r}(x) \vee \textsf{r}(\textsf{f}(x)).$ We can perform the same split as before, but if we ignore the fairness condition that $\mathcal {J}_i \models ({{\mathscr {N}}}_i)_\bot$ must hold infinitely often, we can stick to the interpretation $\{\lnot \textsf{v}_1, \lnot \textsf{v}_2\}$ and derive useless consequences of the form $\textsf{r}(\textsf{f}^i(\textsf{a}))$ forever, thereby failing to generate $\bot .$ Similarly, the SAT solver must be invoked eventually after deriving a propositional clause $\bot \mathbin {\leftarrow }A$ that conflicts with the current interpretation.

#### Example 44

Consider the consistent set consisting of $\lnot \textsf{p}(x),$ $\textsf{p}(\textsf{a}) \vee \textsf{q}(\textsf{a}),$ and $\lnot \textsf{q}(y) \vee \textsf{p}(\textsf{f}(y)) \vee \textsf{q}(\textsf{f}(y)).$ Splitting the second clause into $\textsf{p}(\textsf{a})$ and $\textsf{q}(\textsf{a})$ and resolving $\textsf{q}(\textsf{a})$ with the third clause yields $\textsf{p}(\textsf{f}(\textsf{a})) \vee \textsf{q}(\textsf{f}(\textsf{a})).$ This process can be iterated to yield arbitrarily many applications of $\textsf{f}$. Now suppose that $\textsf{v}_{2i}$ and $\textsf{v}_{2i+1}$ are associated with $\textsf{p}(\textsf{f}^i(\textsf{a}))$ and $\textsf{q}(\textsf{f}^i(\textsf{a})),$ respectively. If we split every emerging clause $\textsf{p}(\textsf{f}^i(\textsf{a})) \vee \textsf{q}(\textsf{f}^i(\textsf{a}))$ and the SAT solver always makes $\textsf{v}_{2i}$ true before $\textsf{v}_{2i+1}$, we end up with the situation of Example 32 and Fig. 1. For the limit point $\mathcal J$, all $\textit{FInf}{}$-inferences are performed. Thus, the derivation is fair.

#### Example 45

We build a clause set from two copies of Example 44, where each clause *C* from each copy $i \in \{1, 2\}$ is extended to $\lnot \textsf{r}_i \vee C$. We add the clause $\textsf{r}_1 \vee \textsf{r}_2$ and split it as our first move. From there, each branch imitates Example 44. A SAT solver might jump back and forth between them, as in Example 33 and Fig. 2. Even if the A-clauses get disabled and re-enabled infinitely often, we must select them eventually and perform all nonredundant inferences in at least one of the two limit points ($\mathcal J'$ or $\mathcal J''$).

## Locking Provers

With both AVATAR and labeled splitting, an enabled A-clause can be redundant locally, w.r.t. the current interpretation, and yet nonredundant globally. Both architectures provide mechanisms to temporarily lock away such A-clauses and unlock them when coming back to an interpretation where they are no longer locally redundant. In AVATAR, conditionally deleted A-clauses are stored in the locked set; in labeled splitting, they are stored in the split stack. We will refine the model-guided prover into a locking prover that captures these mechanisms.

### The Transition Rules

The states of a locking derivation are triples $(\mathcal {J}, \mathscr {N}, \mathscr {L})$, where $\mathcal {J}$ is a propositional interpretation, $\mathscr {N} \subseteq {\textbf {AF}}$ is a set of A-formulas, and $\mathscr {L} \subseteq {{\mathscr {P}}}_\text {fin}({{\textbf {A}}})\times {\textbf {AF}}$ is a set of pairs of finite assertion sets and A-formulas. Intuitively, $(B{,}\; C \mathbin {\leftarrow }A) \in \mathscr {L}$ means that $C \mathbin {\leftarrow }A$ is “locally redundant” in all interpretations $\mathcal J \supseteq B$. The function  erases the locks:  Initial states have the form $(\mathcal J, N, \emptyset )$, where $N \subseteq {\textbf{F}}.$ The *locking prover* is defined by two transition rules:The Lift rule performs an $\Longrightarrow _{{{\textsf{M}}}{{\textsf{G}}}}$-transition and unlocks any A-formulas that are no longer locally redundant. The Lock rule can be used to lock A-formulas that are locally redundant.

#### Lemma 46

Let $(\mathcal {J}_i, \mathscr {N}_i, \mathscr {L}_i)_i$ be an $\Longrightarrow _{{\textsf{L}}}$-derivation. Then  is an $\Longrightarrow _{{{\textsf{M}}}{{\textsf{G}}}}$-derivation.

#### Proof

Every Lift transition clearly corresponds to an ${{\textsf{M}}}{{\textsf{G}}}$ transition. Every Lock transition corresponds to a Derive transition with $\mathscr {M} = \mathscr {M}' = \emptyset .$ $\square$

#### Example 47

Let $\mathcal {J}_0 = \{\lnot \textsf{v}_0\}$ and $\mathcal {J}_1 = \{\textsf{v}_0\}$. The following derivation based on first-order resolution illustrates the locking and unlocking of an A-clause:Gray boxes indicate enabled unlocked clauses. We first put a lock on $\textsf{p}(\textsf{a})$, because it is “locally subsumed” by $\textsf{p}(\textsf{x}) \mathbin {\leftarrow }\{\lnot \textsf{v}_0\}$ in $\mathcal J_0$. Once we switch to $\mathcal J_1$, the lock is released, and we can use $\textsf{p}(\textsf{a})$ to conclude the refutation.

There are three things to note. First, if we had simply thrown away the clause $\textsf{p}(\textsf{a})$ instead of locking it, we would have lost refutability. Second, it would have been advantageous not to lock $\textsf{p}(\textsf{a})$ at all and to use it immediately to derive $\bot$; however, it is not difficult to come up with examples where locking actually helps, which is why AVATAR includes this mechanism. Third, although the derivation shows only “local subsumption,” it could easily be changed to perform “local simplification”—e.g., demodulation from an equation $s \approx t \mathbin {\leftarrow }A$.

### Counterexamples

Locking can cause incompleteness, because an A-formula can be locally redundant at every point in the derivation and yet not be so at any limit point, thereby breaking local saturation. For example, if we have derived $\textsf{p}(x) \mathbin {\leftarrow }\{\lnot \textsf{v}_k\}$ for every *k*,  then $\textsf{p}(\textsf{c})$ is locally redundant in any interpretation $\mathcal {J}$ that contains $\lnot \textsf{v}_k.$ If the sequence of interpretations is given by $\mathcal {J}_i = \{ \textsf{v}_0, \dots , \textsf{v}_{i-1}, \lnot \textsf{v}_i, \lnot \textsf{v}_{i+1}, \ldots \},$ the clause $\textsf{p}(\textsf{c})$ would always be locally redundant and never be considered for inferences. Yet $\textsf{p}(\textsf{c})$ might not be locally redundant at the unique limit point $\mathcal {J} = {{\textbf {V}}}$.

#### Example 48

Consider the inconsistent initial clause setand ordered resolution with selection as the calculus. Assume that the selection function always chooses the maximal negative literals w.r.t. the precedence $\textsf{p} \prec \textsf{q} \prec \textsf{r} \prec \textsf{s} \prec \textsf{t}.$ Let $\textit{fml}(\textsf{v}_i) = \lnot \textsf{r}(\textsf{f}^i(\textsf{a}),x) \vee \textsf{q}(x)$ and $\textit{fml}(\textsf{w}_i) = \lnot \textsf{s}(\textsf{f}^i(\textsf{a})),$ and let $\textsf{v}_i$ and $\textsf{w}_i$ be false in the initial model for all *i*. Following an age-based selection heuristic and maximal splitting, the second clause the prover derives is $\lnot \textsf{s}(\textsf{a}) \vee \lnot \textsf{r}(\textsf{a}, y) \vee \textsf{q}(y)$, which it splits into $\lnot \textsf{s}(\textsf{a}) \mathbin {\leftarrow }\{\textsf{w}_0\}$ and $\lnot r(\textsf{a}, y) \vee q(y) \mathbin {\leftarrow }\{\textsf{v}_0\}.$ The $\textsf{s}$ predicate’s role is purely to ensure that this clause is split and that the assertion $\textsf{v}_0$ is introduced. The prover later also derives $\textsf{q}(x) \vee \textsf{p}(x)$ and $\textsf{q}(y) \mathbin {\leftarrow }\{\textsf{v}_0\}$ and switches to a model in which $\textsf{v}_i$ is true if and only if $i = 0$. The first of the two clauses is clearly locally redundant, so Lock applies, and $(\{\textsf{v}_0\}{,}\> \textsf{q}(x) \vee \textsf{p}(x))$ is added to $\mathscr {L}$.

Next, $\textsf{q}(y) \mathbin {\leftarrow }\{\textsf{v}_1\}$ is derived, before $\textsf{q}(y) \mathbin {\leftarrow }\{\textsf{v}_0\}$ is selected for inferences. Eventually, that latter clause can be used together with $\lnot \textsf{q}(\textsf{c})$ to derive $\bot \mathbin {\leftarrow }\{\textsf{v}_0\}$. The prover then switches to a new model in which $\textsf{v}_i$ is true if and only if $i = 1$. The clause $\textsf{q}(x) \vee \textsf{p}(x)$ can immediately be relocked. This process can be repeated indefinitely. The clause $\textsf{q}(x) \vee \textsf{p}(x)$, which is necessary for a refutation (together with $\lnot \textsf{p}(\textsf{c})$ and $\lnot \textsf{q}(\textsf{c})$), is ignored because it is always locally redundant. It is locked each time the prover selects an A-clause for inferences, due to a different A-clause. But it is not locally redundant at the limit point $\mathcal J = \lnot {{\textbf {V}}}$.

In the derivation in Example 48, locking is not applied exhaustively: The A-clause $\lnot \textsf{r}(\textsf{f}^i(\textsf{a}), y) \vee \textsf{q}(y) \mathbin {\leftarrow }\{ \textsf{v}_i \}$ is not locked, even though $\textsf{q}(y) \mathbin {\leftarrow }\{ \textsf{v}_j \}$ has already been derived. This situation is unrealistic and would not happen in Vampire. We could hope that is enough for completeness to forbid such anomalous scenarios. However, this is not the case, as we can see from a more complicated example:

#### Example 49

The calculus is ordered resolution with selection using the precedence $\textsf{p} \prec \textsf{q}_1 \prec \textsf{q}_2 \prec \textsf{r}_1 \prec \textsf{r}_2 \prec \textsf{s} \prec \textsf{t}_1 \prec \textsf{t}_2 \prec \textsf{u},$ selecting nothing if the clause is of the form $\lnot \textsf{u}(\ldots ) \vee \textsf{u}(\ldots )$ and otherwise selecting the maximal negative literals.

The initial clauses are as follows. First, we have a splittable clause $\textsf{q}_1(x) \vee \textsf{q}_2(y).$ Then we have clauses $\textsf{u}(\textsf{a}, y)$ and $\lnot \textsf{u}(x, y) \vee \textsf{u}(\textsf{f}(x), y).$ We will use the predicate symbol $\textsf{u}$ to delay the selection of a clause in an age-based selection heuristic, by adding a literal $\lnot \textsf{u}(\textsf{f}^j(x), y)$ to the clause. Moreover, we have the clause $\textsf{s}(x,y)$. We can prevent splitting by adding the literal $\lnot \textsf{s}(x,y)$ to a clause. Finally, we add the following clauses:The initial clause set is clearly inconsistent. Yet we will sketch an infinite derivation that corresponds to an age-based selection heuristic and that does not derive $\bot .$ First, we split $\textsf{q}_1(x) \vee \textsf{q}_2(y)$ into $\textsf{q}_1(x) \mathbin {\leftarrow }\{\textsf{x}_1\}$ and $\textsf{q}_2(x) \mathbin {\leftarrow }\{\textsf{x}_2\}$, where the assertion denotations are as follows:The derivation uses the following sequence of interpretations $\mathcal J_i$:

- $\mathcal J_i \models \textsf{v}_j$ if and only if $j < i$;
- $\mathcal J_i \models \textsf{w}_j$ if and only if $j \in \{i, i+1\}$;
- $\mathcal J_i \models \textsf{x}_1$ if and only if *i* is even;
- $\mathcal J_i \models \textsf{x}_2$ if and only if *i* is odd.

The derivation thus alternates between two families of interpretations, with even and odd indices, giving rise to two limit points.

After the clause $\textsf{q}_1(x) \vee \textsf{q}_2(y)$ is split, the prover is in the model $\mathcal J_0$ and derives the clauses $\lnot \textsf{u}(y, x) \vee \textsf{r}_1(x) \vee \lnot \textsf{q}_1(x),$ $\lnot \textsf{u}(y, x) \vee \textsf{r}_2(x) \vee \lnot \textsf{q}_2(x),$ $\lnot \textsf{s}(\textsf{a}, y) \vee \textsf{r}_1(y) \vee \lnot \textsf{p}(\textsf{a}).$ The last clause is split into $\lnot \textsf{s}(\textsf{a}, y) \vee \textsf{r}_1(y) \mathbin {\leftarrow }\{\textsf{w}_0\},$ $\lnot \textsf{p}(\textsf{a}) \mathbin {\leftarrow }\{\textsf{v}_0\},$ and $\bot \mathbin {\leftarrow }\{\lnot \textsf{v}_0, \lnot \textsf{w}_0\}.$ Then an analogous split happens with $\textsf{r}_2$ instead of $\textsf{r}_1.$ After a few more inferences, we derive $\textsf{r}_1(x) \vee \lnot \textsf{q}_1(x)$ and then $\textsf{r}_1(y) \mathbin {\leftarrow }\{\textsf{w}_0\},$ which makes $\textsf{r}_1(x) \vee \lnot \textsf{q}_1(x)$ locally redundant (and analogously for $\textsf{r}_2$ in place of $\textsf{r}_1$). Once $\textsf{r}_1(y) \mathbin {\leftarrow }\{\textsf{w}_0\}$ is picked as the given clause, the prover derives $\bot \mathbin {\leftarrow }\{\textsf{w}_0\}$ and switches to the next model $\mathcal J_1$.

In the first family, $\mathcal {J}_{2i},$ the clause $\lnot \textsf{q}_1(x) \vee \textsf{r}_1(x)$ is always locally redundant due to $\textsf{r}_1(x) \mathbin {\leftarrow }\{\textsf{w}_{2i}\},$ and is locked with the assertion $\textsf{w}_{2i}.$ Similarly, $\lnot \textsf{q}_2(x) \vee \textsf{r}_2(x)$ is locally redundant in the second family, $\mathcal {J}_{2i+1},$ with the assertion $\textsf{w}_{2i+1}.$ In both cases we can already lock each of the clauses while the prover is still in the previous model ($\mathcal J_{2i-1}$ and $\mathcal J_{2i}$, respectively).

The clause $\lnot \textsf{q}_2(x) \vee \textsf{r}_2(x)$ is thus only ever unlocked in interpretations $\mathcal {J}_{2i}.$ In those interpretations, $\textsf{q}_2(x) \mathbin {\leftarrow }\{\textsf{x}_2\}$ is disabled and hence no inferences can be performed with $\lnot \textsf{q}_2(x) \vee \textsf{r}_2(x).$ The same holds *mutatis mutandis* for $\lnot \textsf{q}_1(x) \vee \textsf{r}_1(x),$ which is unlocked only when no inferences can be performed with it. As a result, the derivation never performs inferences with $\textsf{q}_1(x) \mathbin {\leftarrow }\{\textsf{x}_1\}$ or $\textsf{q}_2(x) \mathbin {\leftarrow }\{\textsf{x}_2\}.$ Removing these A-clauses makes the set satisfiable; thus, by soundness, the derivation cannot contain $\bot .$

Given the right sequence of propositional interpretations returned by the SAT solver, the derivation in Example 49 could potentially happen in a prover such as Vampire. It is difficult to exclude that the SAT solver used by Vampire could produce this sequence, or generally to characterize the sequence of models produced by SAT solvers in such a concrete way. This derivation is also strongly fair—every inference that is possible infinitely often, perhaps intermittently, is eventually made redundant. Thus strong fairness is not a sufficient criterion for completeness either.

### Fairness

Our solution to the issues encountered above is as follows. Let $(\mathcal {J}_i, \mathscr {N}_i, \mathscr {L}_i)_i$ be an $\Longrightarrow _{{\textsf{L}}}$-derivation. Given a subsequence $(\mathcal {J}'_j)_j$ of $(\mathcal {J}_i)_i$, let $(\mathscr {N}'_j)_j$ be the corresponding subsequence of $(\mathscr {N}_i)_i.$ To achieve fairness, we now consider $\mathscr {N}'_\infty$, the A-formulas persistent in the subsequence $(\mathscr {N}'_j)_j$. By contrast, with no A-formulas locked away, fairness of $\Longrightarrow _{{{\textsf{M}}}{{\textsf{G}}}}$-derivations could use $\mathscr {N}_\infty$.

#### Definition 50

An $\Longrightarrow _{{\textsf{L}}}$-derivation $(\mathcal {J}_i, \mathscr {N}_i, \mathscr {L}_i)_i$ is *fair* if (A) $\mathscr {L}_0 = \emptyset$ and (B) either (1) $\bot \in \bigcup _i \mathscr {N}_i$ or (2) $\mathcal {J}_i \models (\mathscr {N}_i)_\bot$ for infinitely many indices *i* and there exists a subsequence $(\mathcal {J}'_j)_j$ converging to a limit point $\mathcal {J}$ such that , where $(\mathscr {N}'_j)_j$ and $(\mathscr {L}'_j)_j$ correspond to $(\mathcal J'_j)_j$.

Fairness of an $\Longrightarrow _{{\textsf{L}}}$-derivation implies fairness of the corresponding $\Longrightarrow _{{{\textsf{M}}}{{\textsf{G}}}}$-derivation. The condition on the sets $\mathscr {L}'_j$ ensures that inferences from A-formulas that are locked infinitely often, but not infinitely often with the same lock, are redundant at the limit point. In particular, if we know that each A-formula is locked at most finitely often, then  and the inclusion in the definition above simplifies to .

#### Theorem 51

**(Fairness)** Let $(\mathcal {J}_i, \mathscr {N}_i, \mathscr {L}_i)_i$ be a fair $\Longrightarrow _{{\textsf{L}}}$-derivation. Then  is a fair $\Longrightarrow _{{{\textsf{M}}}{{\textsf{G}}}}$-derivation.

#### Proof

We already showed that $(\mathcal {J}_i, \mathscr {N}_i, \mathscr {L}_i)_i$ is an $\Longrightarrow _{{{\textsf{M}}}{{\textsf{G}}}}$-derivation in Lemma 46. It remains to show fairness. If the $\Longrightarrow _{{\textsf{L}}}$-derivation is fair by case (1) of Definition 50, we apply case (1) of Definition 36 to show that the $\Longrightarrow _{{{\textsf{M}}}{{\textsf{G}}}}$-derivation is fair. Otherwise, from case (2) of Definition 50, we retrieve a limit point $\mathcal J$. We will show, for that limit point, case (2) of Definition 36:Assume (a)  By the definition of fairness of $\Longrightarrow _{{\textsf{L}}}$-derivations, if all of $\iota$’s premises belong to , then $\iota$ is redundant. Otherwise, we have that (b) one of $\iota$’s premises, *C*, is not in that set; that is, $C \notin (\mathscr {N}'_\infty ) _{\mathcal J}$ and either  or .

By (a) we have that  for some $A \subseteq \mathcal J$. Since $C \notin ({\mathscr {N}'_\infty })_{\mathcal J}$ by (b), $C \mathbin {\leftarrow }A$ cannot be persistent in $(\mathscr {N}'_j)_j$ and hence must occur infinitely often in the sequence . Thus  and therefore  by (b).

Hence $(B{,}\> C \mathbin {\leftarrow }A') \in \mathscr {L}'^\infty$ for some $A' \subseteq \mathcal J$ and *B*. If $(B, C \mathbin {\leftarrow }A') \in \mathscr {L}'_j$ for some *j*,  then necessarily $B \subseteq \mathcal J'_j$ due to the side conditions of the $\Longrightarrow _{{\textsf{L}}}$-transitions. Since this is true for infinitely many indices *j*, we also have $B \subseteq \mathcal J'^\infty = \mathcal J,$ and thus $C \in \textit{FRed}( (\mathscr {N}_i) _{\mathcal J} )$ for some *i* by the side condition of the Lock transition. Therefore, by reducedness of $\textit{FRed}$, the inference $\iota$ is redundant. $\square$

#### Corollary 52

**(Dynamic completeness)**  Assume $(\textit{FInf}{},\textit{FRed})$ is statically complete. Given a fair $\Longrightarrow _{{\textsf{L}}}$-derivation $(\mathcal {J}_i, {{\mathscr {N}}}_i, {{\mathscr {L}}}_i)_i$ such that ${{\mathscr {N}}}_0 \models \{\bot \},$ we have $\bot \in {{\mathscr {N}}}_i$ for some *i*.

#### Proof

By Theorems 28 and 39. $\square$

## AVATAR-Based Provers

AVATAR was unveiled in 2014 by Voronkov [28], although it was reportedly present in Vampire already in 2012. Since then, he and his colleagues studied many options and extensions [4, 22]. At least two reimplementations exist, in Ebner’s super tactic for Lean [13] and in the Drodi prover by Oscar Contreras. Here we attempt to capture AVATAR’s essence.

We will define an abstract AVATAR-based prover that extends the locking prover ${\textsf{L}}$ with a given clause procedure [19, Sect. 2.3]. A-formulas are moved in turn from the passive to the active set, where inferences are performed. The heuristic for choosing the next *given* A-formula to move is guided by timestamps indicating when the A-formulas were derived, to ensure fairness.

### The Transition Rules

Let $\textbf{TAF}= {\textbf {AF}}\times {\mathbb {N}}$ be the set of *timestamped A-formulas*. (We will often omit the adjective “timestamped.”) Given a subset $\mathcal {N} \subseteq \textbf{TAF},$ we define  and overload existing notations to erase timestamps as necessary. Accordingly,   and . Note that we use a new set of calligraphic letters (e.g., $\mathcal {C}, \mathcal {N}$) to range over timestamped A-formulas and timestamped A-formula sets. We say that $\mathcal {N}$ is enabled in $\mathcal J$ if and only if  is enabled in $\mathcal J$. We also define  for $\textbf{TAF}$-inferences $\iota .$

Using the saturation framework [29, Sect. 3], we lift a calculus $(\textit{SInf}{}, \textit{SRed})$ on ${\textbf {AF}}$ to a calculus $(\textit{TSInf}{}, \textit{TSRed})$ on $\textbf{TAF}$ with the tiebreaker order < on timestamps. The tiebreaker is used to strengthen redundancy, so that if the same A-formula appears but with two different timestamps, the more recent version is considered redundant. In other words, if an A-formula appears with two timestamps, the later version is redundant. Note that $\textit{TSRed}$ is in general not reduced. Traditionally, provers use the active or passive status as tiebreaker: An active clause may subsume a passive copy of itself, but not the other way around. Timestamps are more fine-grained.

#### Lemma 53

Let $\mathcal {N} \subseteq {\textbf {AF}},$ $\mathscr {C} \in {\textbf {AF}},$ and $t,k \in \mathbb N.$ Then:

;
;
; and
4. $$(\mathscr {C}, t + k) \in \textit{TSRed}_\text {F}(\{(\mathscr {C}, t)\})$$ if $$k > 0$$.

#### Proof

This follows directly from the definition in Waldmann et al. [29]. $\square$

A state is a tuple $(\mathcal {J}, \mathcal {A}, \mathcal {P}, \mathcal {Q}, \mathcal {L})$ consisting of a propositional interpretation $\mathcal {J}$, a set of enabled nonpropositional *active* A-formulas $\mathcal {A} \subseteq \textbf{TAF}$, a set of enabled nonpropositional *passive* A-formulas $\mathcal {P} \subseteq \textbf{TAF}$, a set of A-formulas $\mathcal {Q} \subseteq \textbf{TAF}$ that are either disabled in $\mathcal {J}$ or propositional clauses, and a set of locked A-formulas $\mathcal {L} \subseteq {{\mathscr {P}}}_\text {fin}({{\textbf {A}}})\times \textbf{TAF}$ such that

1. $\mathcal {A}_\bot = \mathcal {P}_\bot = \emptyset$;(2)$\mathcal {A} \cup \mathcal {P}$ is enabled in $\mathcal {J}$;(3)$\mathcal {Q}_{\mathcal {J}} \subseteq \{\bot \}$.

Whenever we write a tuple $(\mathcal {J}, \mathcal {A}, \mathcal {P}, \mathcal {Q}, \mathcal {L})$, we assume that it satisfies all these invariants. For every $\mathcal {L} \subseteq {{\textbf {A}}}\times \textbf{TAF}$, we define 

The input formulas are first put in the passive set $\mathcal {P}$. Once an A-formula is selected for inferences and all inferences with it and the active A-formulas have been made redundant, it is moved to the active set $\mathcal {A}$. Inferences such as Split produce disabled and propositional clauses, which are put into $\mathcal {Q}$. When switching to a new model, the prover moves the newly enabled A-formulas from $\mathcal {Q}$ to $\mathcal {P}$ and the newly disabled A-formulas from $\mathcal {A}$ and $\mathcal {P}$ to $\mathcal {Q}$ to preserve the state invariant.

The division of nonactive A-formulas into the sets $\mathcal {P}$ and $\mathcal {Q}$ is done for notational convenience; for example, $\mathcal {P}$ is a separate set because fairness will be stated in terms of $\mathcal {A}$ and $\mathcal {P}$. In a practical implementation, this division would likely be different. The set $\mathcal {Q}$ would typically be distributed over two data structures: The propositional clauses in $\mathcal {Q}_\bot$ are directly passed to the SAT solver and need not be stored by the prover itself. The remaining A-formulas $\mathcal {Q} \setminus \mathcal {Q}_\bot$ are those that need to be moved back into $\mathcal {P}$ when the prover switches to an interpretation that enables them. These might be stored in the same data structure as the set of locked A-formulas $\mathcal {L},$ which also need to be reactivated depending on the interpretation. This is what Vampire does. Alternatively, they could be stored in the same data structure as $\mathcal {P},$ with the prover checking on every access whether an A-formula is enabled in the current interpretation.

The *AVATAR-based prover* ${{\textsf{A}}}{{\textsf{V}}}$ is defined as the following transitions:There is also a LockP rule that is identical to LockA except that it starts in the state $(\mathcal {J}, \mathcal {A}, \mathcal {P} \uplus \{(C \mathbin {\leftarrow }A{,}\>t)\}, \mathcal {Q}, \mathcal {L})$. An ${{\textsf{A}}}{{\textsf{V}}}$-derivation is *well timestamped* if every A-formula introduced by a rule is assigned a unique timestamp. In practice, a prover would ensure well-timestampedness by assigning timestamps monotonically, but this is not necessary for the fairness and completeness proofs.

#### Lemma 54

Let $(\mathcal {J}_i, \mathcal {A}_i, \mathcal {P}_i, \mathcal {Q}_i, \mathcal {L}_i)_i$ be an $\Longrightarrow _{{{\textsf{A}}}{{\textsf{V}}}}$-derivation. Then  is an $\Longrightarrow _{{\textsf{L}}}$-derivation.

#### Proof

The transitions map directly to the corresponding transitions in $\Longrightarrow _{{\textsf{L}}}$; both Infer and Process map to a Lift of Derive. $\square$

#### Lemma 55

Let $(\mathcal {J}_i, \mathcal {A}_i, \mathcal {P}_i, \mathcal {Q}_i, \mathcal {L}_i)_i$ be an $\Longrightarrow _{{{\textsf{A}}}{{\textsf{V}}}}$-derivation such that $\mathcal {A}_0 = \emptyset$. Then  for every *i*.

#### Proof

The invariant is preserved by each transition. $\square$

#### Example 56

Let us redo the $\Longrightarrow _{{{\textsf{M}}}{{\textsf{G}}}}$-derivation of Example 30 using $\Longrightarrow _{{{\textsf{A}}}{{\textsf{V}}}}$. For readability, we emphasize in gray the A-clauses that appear or move between state components and omit all timestamps. One possible derivation is 
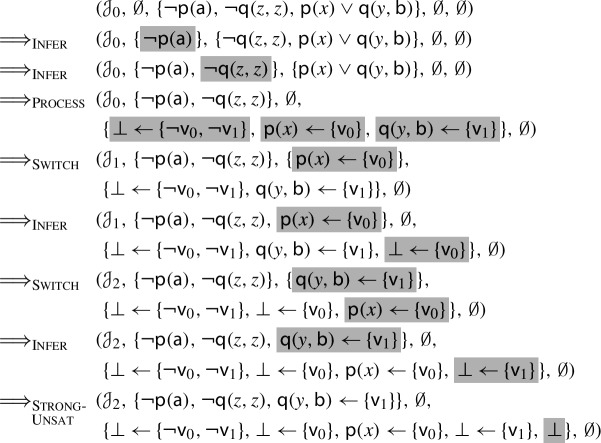


#### Example 57

Let us redo the $\Longrightarrow _{{\textsf{L}}}$-derivation of Example 47 using $\Longrightarrow _{{{\textsf{A}}}{{\textsf{V}}}}$, following the same conventions as in the previous example. One possible derivation is 
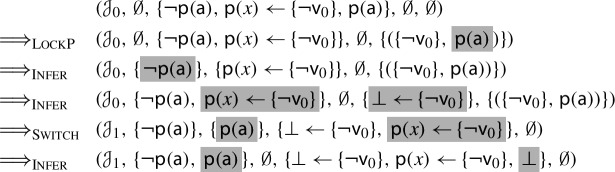


### Counterexamples

In contrast with nonsplitting provers, for ${{\textsf{A}}}{{\textsf{V}}}$, fairness w.r.t. formulas does not imply fairness w.r.t. inferences. To ensure fairness in a nonsplitting prover, it suffices to select the oldest formula for inferences infinitely often; for example, provers can alternate between choosing the oldest and choosing the heuristically best formula. In splitting provers, such a strategy is incomplete, and we need an even stronger fairness criterion.

A problematic scenario involves two premises $\mathcal {C}, \mathcal {D}$ of a binary inference $\iota$ and four transitions repeated forever, with other steps interleaved: Infer makes $\mathcal {C}$ active; Switch disables it; Infer makes $\mathcal {D}$ active; Switch disables it. Even though $\mathcal {C}$ and $\mathcal {D}$ are selected in a strongly fair fashion, $\iota$ is never performed.

#### Example 58

More concretely, make two copies of the clause set

$$\begin{aligned}\begin{aligned}\{ \lnot \textsf{p}(x) \vee \textsf{p}(\textsf{f}(x)) \vee \textsf{q}(x) {,}\qquad \textsf{p}(\textsf{a}){,}\qquad \lnot \textsf{q}(x){,}\qquad \lnot \textsf{p}(x) \vee \textsf{q}(\textsf{f}(x)) \}\end{aligned}\end{aligned}$$

one with the assertion $\{\textsf{x}_1\}$, the other with $\{\textsf{x}_2\}$, in addition to the propositional clause $\bot \mathbin {\leftarrow }\{\lnot \textsf{x}_1, \lnot \textsf{x}_2\}$. Suppose the prover starts with the model $\{\textsf{x}_1\}$ and processes the clauses in the order given above. It first chooses the A-clause $\lnot \textsf{p}(x) \vee \textsf{p}(\textsf{f}(x)) \vee \textsf{q}(x) \mathbin {\leftarrow }\{\textsf{x}_1\}$ for inferences, followed by $\textsf{p}(\textsf{a}) \mathbin {\leftarrow }\{\textsf{x}_1\}.$ Let $\textit{fml}(\textsf{v}_i) = \textsf{p}(\textsf{f}^i(\textsf{a}))$ and $\textit{fml}(\textsf{w}_i) = \textsf{q}(\textsf{f}^i(\textsf{a})).$ By resolution and splitting, it derives $\textsf{p}(\textsf{f}(\textsf{a})) \mathbin {\leftarrow }\{\textsf{v}_1\},$ $\textsf{q}(\textsf{a}) \mathbin {\leftarrow }\{\textsf{w}_0\},$ and $\bot \mathbin {\leftarrow }\{\textsf{x}_1,\lnot \textsf{v}_1,\lnot \textsf{w}_0\}.$ It then switches to a model in which $\textsf{x}_2$ is true. There it selects $\lnot \textsf{p}(x) \vee \textsf{p}(\textsf{f}(x)) \vee \textsf{q}(x) \mathbin {\leftarrow }\{\textsf{x}_2\}$ and $\textsf{p}(\textsf{a}) \mathbin {\leftarrow }\{\textsf{x}_2\}$ for inferences, deriving analogous A-clauses as in the $\textsf{x}_1$ branch.

Let the models alternate between the $\textsf{x}_1$ and $\textsf{x}_2$ branches, making as few variables true as possible. Because the models alternate between the two branches, $\lnot \textsf{p}(x) \vee \textsf{p}(\textsf{f}(x)) \vee \textsf{q}(x) \mathbin {\leftarrow }\{\textsf{x}_i\}$ will always be the oldest passive A-clause after switching to a new model. Assume that the prover chooses this clause for inferences based on age. If we are allowed to interleave age-based selection with heuristic selection, we can cause the prover to switch model after selecting at most two additional A-clauses for inferences: If an A-clause $\textsf{q}(\textsf{f}^j(\textsf{a})) \mathbin {\leftarrow }\{\textsf{w}_j\}$ is enabled, we heuristically select both that A-clause and $\lnot \textsf{q}(x) \mathbin {\leftarrow }\{\textsf{x}_i\}$. Otherwise, an A-clause of the form $\textsf{p}(\textsf{f}^j(\textsf{a})) \mathbin {\leftarrow }\{\textsf{v}_j\}$ is enabled. Assume that *j* is maximal among such clauses, and that thus $\textsf{v}_{j+1}$ is false in the model. We heuristically select that clause for inferences, deriving $\bot \mathbin {\leftarrow }\{\lnot \textsf{v}_{j+1}, \lnot \textsf{w}_j, \textsf{v}_j, \textsf{x}_i\}$ by splitting $\textsf{p}(\textsf{f}^{j+1}(\textsf{a})) \vee \textsf{q}(\textsf{f}^j(\textsf{a})) \mathbin {\leftarrow }\{\textsf{v}_j, \textsf{x}_i\}.$

With this strategy, the prover will never select $\lnot \textsf{p}(x) \vee \textsf{q}(\textsf{f}(x)) \mathbin {\leftarrow }\{\textsf{x}_i\}$ for inferences, since there is always an older clause to choose first. Consequently, it will never derive $\bot$.

In Example 58, the prover did not derive $\bot$ because it never performed an inference between $\textsf{p}(\textsf{a}) \mathbin {\leftarrow }\{\textsf{x}_1\}$ and $\lnot \textsf{p}(x) \vee \textsf{q}(\textsf{f}(x)) \mathbin {\leftarrow }\{\textsf{x}_1\}$ (and analogously for $\textsf{x}_2$), even though both A-clauses are enabled infinitely often. Forbidding this situation does not guarantee completeness either. As Example 49 showed, there exist strongly fair derivations that do not derive $\bot$ from an inconsistent initial set.

We believe that this counterexample cannot arise with Vampire, because Vampire alternates between age-based and weight-based selection using a fixed ratio (the “age–weight ratio” or “pick–given ratio”). In contrast, our example requires a highly unrestricted heuristic selection, where we choose young, large A-clauses such as $\textsf{p}(\textsf{f}^n(\textsf{a})) \mathbin {\leftarrow }\{\textsf{v}_n\}$ even though smaller, older ones are enabled.

Unrelated to completeness, we might expect that under a reasonable strategy an $\Longrightarrow _{{{\textsf{A}}}{{\textsf{V}}}}$-derivation saturates every limit point. This is, however, not the case either, even with strict age-based selection:

#### Example 59

Take the following consistent A-clause set:Assume ordered resolution as the base calculus with the precedence $\textsf{q} \prec \textsf{p}.$ Thus the prover will not resolve $\lnot \textsf{q}(x) \mathbin {\leftarrow }\{\textsf{x}\}$ with $\lnot \textsf{p}(x) \vee \textsf{p}(\textsf{f}(x)) \vee \textsf{q}(\textsf{f}(x)) \mathbin {\leftarrow }\{\textsf{x}\}.$ We will sketch a derivation with two limit points, $\mathcal J \models \textsf{x}$ and $\mathcal J' \not \models \textsf{x},$ where $\mathcal J'$ will not be locally saturated. Let $\textit{fml}(\textsf{v}_i) = \textsf{p}(\textsf{f}^i(\textsf{a}))$ and $\textit{fml}(\textsf{w}_i) = \textsf{q}(\textsf{f}^i(\textsf{a})).$ We define the sequence of models $(\mathcal J_i)_i$ such thatThe prover now starts in the model $\mathcal J_0,$ and processes the formulas in the order we listed them at the beginning of the example. The first new formula it derives is $\textsf{p}(\textsf{f}(\textsf{a})) \vee \textsf{q}(\textsf{f}(\textsf{a})) \mathbin {\leftarrow }\{\textsf{x}\},$ which it splits into $\textsf{p}(\textsf{f}(\textsf{a})) \mathbin {\leftarrow }\{\textsf{v}_1\},$ $\textsf{q}(\textsf{f}(\textsf{a})) \mathbin {\leftarrow }\{\textsf{w}_1\},$ and $\bot \mathbin {\leftarrow }\{\textsf{x}, \lnot \textsf{v}_1, \lnot \textsf{w}_1\}.$ The last propositional clause is not satisfied in $\mathcal J_0,$ so the prover switches to a new interpretation.

After switching to the next model $\mathcal J_1 = \mathcal J_{2 \cdot 0 + 1},$ the two formulas $\textsf{p}(\textsf{f}(\textsf{a})) \mathbin {\leftarrow }\{\textsf{v}_1\}$ and $\textsf{q}(\textsf{f}(\textsf{a})) \mathbin {\leftarrow }\{\textsf{w}_1\}$ remain in the active set. The prover then chooses the oldest enabled passive formula, $\lnot \textsf{q}(x) \mathbin {\leftarrow }\{\lnot \textsf{x}\},$ for inferences. Thus deriving the propositional clause $\bot \mathbin {\leftarrow }\{\lnot \textsf{x}, \textsf{w}_1\},$ which is not satisfied in $\mathcal J_1.$

This process is then repeated infinitely often. In the model $\mathcal J_{2i},$ the prover derives the three new formulas $\textsf{p}(\textsf{f}^{i+1}(\textsf{a})) \mathbin {\leftarrow }\{\textsf{v}_{i+1}\},$ $\textsf{q}(\textsf{f}^{i+1}(\textsf{a})) \mathbin {\leftarrow }\{\textsf{w}_{i+1}\},$ and $\bot \mathbin {\leftarrow }\{\textsf{x}, \lnot \textsf{v}_{i+1}, \lnot \textsf{w}_{i+1}\}.$ The last propositional clause causes a switch to the next model $\mathcal J_{2i+1},$ where $\bot \mathbin {\leftarrow }\{\lnot \textsf{x}, \textsf{w}_{i+1}\}$ is derived.

The subsequence $(\mathcal J_{2i+1})_i$ converges a limit point, call it $\mathcal J'.$ The formulas enabled at $\mathcal J'$ are not saturated: $\textsf{p}(\textsf{a})$ and $\lnot \textsf{p}(x)$ are enabled, but $\bot$ is not.

### Fairness

#### Definition 60

An $\Longrightarrow _{{{\textsf{A}}}{{\textsf{V}}}}$-derivation $(\mathcal {J}_i, \mathcal {A}_i, \mathcal {P}_i, \mathcal {Q}_i, \mathcal {L}_i)_i$ is *fair* if (A) $\mathcal {L}_0 = \emptyset ,$ (B) $\mathcal {A}_0 = \emptyset ,$ and (C) either (1)  or (2) $\mathcal {J}_i \models (\mathcal {Q}_i)_\bot$ for infinitely many indices *i* and there exists a subsequence $(\mathcal {J}'_j)$ converging to a limit point $\mathcal J$ such that (3) $\liminf _{j\rightarrow \infty }\textit{TSInf}{}(\mathcal {A}'_j, \mathcal {P}'_j) = \emptyset$ and (4) 

Condition (3) ensures that all inferences involving passive A-formulas are redundant at the limit point. It would not suffice to simply require $\mathcal {P}'_\infty = \emptyset$ because A-formulas can move back and forth between the sets $\mathcal {A}$, $\mathcal {P}$, and $\mathcal {Q}$, as we saw in Example 58. Condition (4) is similar to the condition on locks in Definition 50.

#### Theorem 61

**(Fairness)** Let $(\mathcal {J}_i, \mathcal {A}_i, \mathcal {P}_i, \mathcal {Q}_i, \mathcal {L}_i)_i$ be a fair $\Longrightarrow _{{{\textsf{A}}}{{\textsf{V}}}}$-derivation. Then the $\Longrightarrow _{{\textsf{L}}}$-derivation  is fair.

#### Proof

We trivially have . Furthermore, if $\bot \in \bigcup _i \mathcal {Q}_i$, we clearly have $\Longrightarrow _{{\textsf{L}}}$-fairness. It remains to show subcase (B)(2) of Definition 50, using the corresponding subsequence as used for $\Longrightarrow _{{{\textsf{A}}}{{\textsf{V}}}}$-fairness. So let  and  be the terms from the corresponding fairness conditions.

First we show  that is, every enabled formula in the subsequence is either persistent or redundant on the base level. So let $(C \mathbin {\leftarrow }A, t) \in P'_j.$ We prove the statement by induction on (*A*, *t*) w.r.t. the lexicographic order. If $C \in \clubsuit _{{\textsf{A}}}{{\textsf{V}}}$ or  we are done. Otherwise  (because ) and hence  (because $C \not \in \clubsuit _{{\textsf{A}}}{{\textsf{V}}}$). So since $(C \mathbin {\leftarrow }A, t)$ is not locked infinitely often, there exists an index after which it is never locked again, which means that it is either persistent in $(\mathcal {A}'_j \cup \mathcal {P}'_j)_j$ (and we are done) or deleted in Process and thus $(C \mathbin {\leftarrow }A, t) \in \bigcup _i \textit{TSRed}_\text {F}(\mathcal {A}_i \cup \mathcal {P}_i \cup \mathcal {Q}_i)$. By definition of $\textit{TSRed}_\text {F},$ either (a) $C \in \bigcup _i \textit{FRed}_\text {F}((\mathcal {A}_i \cup \mathcal {P}_i \cup \mathcal {Q}_i) _{\mathcal J}),$ (b)  for some $B \subset A,$ or (c) $(C \mathbin {\leftarrow }A, s) \in \bigcup _i \mathcal {A}_i \cup \mathcal {P}_i$ for some $s < t.$ In case (a), we are done. In cases (b) and (c), we apply the induction hypothesis.

Now let  and compute 
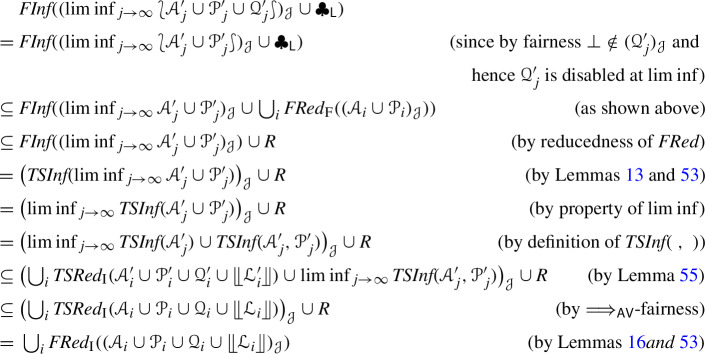
$$\square $$

#### Corollary 62

**(Dynamic completeness)**  Assume $(\textit{FInf}{},\textit{FRed})$ is statically complete. Given a fair $\Longrightarrow _{{{\textsf{A}}}{{\textsf{V}}}}$-derivation $(\mathcal {J}_i, \mathcal {A}_i, \mathcal {P}_i, \mathcal {Q}_i, \mathcal {L}_i)_i$ such that $\mathcal {P}_0 \cup \mathcal {Q}_0 \models \{\bot \},$ we have $\bot \in \mathcal {Q}_i$ for some *i*.

#### Proof

By Theorems 28, 39, and 51. $\square$

Assuming the restriction on locking we already required for $\Longrightarrow _{{\textsf{L}}}$-derivations, the $\Longrightarrow _{{{\textsf{A}}}{{\textsf{V}}}}$ relation is concrete enough to allow us to show that typical clause selection strategies are fair and avoid the counterexamples from Sects. 5.2 and 6.2. Many selection strategies are combinations of basic strategies, such as choosing the smallest formula by weight or the oldest by age. We capture such strategies using selection orders . Intuitively,  if the prover will select $\mathcal {C}$ before $\mathcal {D}$ whenever both are present. That is, the prover always chooses one of the -minimal A-formulas. We use two selection orders: , on timestamped A-formulas, must be followed infinitely often; , on base formulas, must be followed otherwise.

#### Definition 63

Let *X* be a set. A *selection order*  on *X* is an irreflexive and transitive relation such that  is finite for every $x \in X$.

#### Example 64

Let $X \subseteq \textbf{TAF}$ be such that *X* only contains finitely many A-formulas with the same timestamp. Define  on *X* so that  if and only if $t < t'.$ Then  is a selection order corresponding to age-based selection.

#### Remark 65

Every selection order is a well-founded relation, but not every well-founded relation is a selection order. A well order is a selection order if and only if its order type is less than $\omega +1$. The ordinal $\omega +1 = \{ 0< 1< 2< \cdots < \omega \}$ is not a selection order since  is infinite. Even well-founded relations of low rank need not be selection orders: The empty relation $\emptyset \subseteq {\mathbb {N}} \times {\mathbb {N}}$ is irreflexive, transitive, and well founded (with rank zero) but not a selection order.

Selection orders on $\textbf{TAF}$ also generalize the mechanism, outlined by Bachmair and Ganzinger in a footnote [2, Sect. 4.3] and elaborated by Schlichtkrull et al. [27, Sect. 4], of using an ${\mathbb {N}}$-valued weight function that is strictly monotone in the timestamp.

#### Example 66

Let ${\textbf{F}}$ be the set of first-order clauses in a fixed signature. Define the selection order  on ${\textbf{F}}$ by  if and only if $|C'| \le |C|$, where |*C*| denotes the sum of the number of nonvariable positions in *C*. Then  is a selection order because there exists at most a finite number of first-order clauses with at most *n* nonvariable positions for any *n*. This selection order corresponds to a simple weight-based selection scheme.

The intersection of two orders  and  corresponds to the nondeterministic alternation between them. The prover may choose either a -minimal or a -minimal A-formula, at its discretion.

#### Lemma 67

Let  and  be selection orders on *X*. Then  is a selection order as well.

#### Proof

Irreflexivity and transitivity are preserved by intersections, and note that  is finite as a union of two finite sets. $\square$

#### Lemma 68

Let  be a selection order on an infinite set *X*. Then for all elements *x* and *y*,  there exists an element *z* such that  and .

#### Proof

The set  is finite, and therefore  is infinite and in particular nonempty. $\square$

To ensure completeness of the given clause procedure, we must restrict the inferences that the prover may perform; otherwise, it could derive infinitely many A-formulas with different assertions, causing it to switch between two branches of the split tree without making progress as in Example 58. Given $\mathscr {N} \subseteq {\textbf {AF}}$, let 

#### Definition 69

A function $F: {\mathscr {P}}({\textbf {AF}})\rightarrow {\mathscr {P}}({\textbf {AF}})$ is called *strongly finitary* if (1) $\lfloor F(\mathscr {N}) \rfloor$ is finite for every $\mathscr {N} \subseteq {\textbf {AF}}$ such that $\lfloor \mathscr {N} \rfloor$ is finite, and (2) there exists a function $B: {\textbf{F}}\rightarrow {{\mathscr {P}}}_\text {fin}({{\textbf {A}}})$ such that $\lceil F(\mathscr {N}) \rceil \subseteq \lceil \mathscr {N} \rceil \cup B(\lfloor \mathscr {N} \rfloor )$ for every $\mathscr {N} \subseteq {\textbf {AF}}.$

A set of ${\textbf {AF}}$-inferences $\textit{Inf}{}$ is *strongly finitary* if the function $\mathscr {N} \mapsto \textit{concl}(\textit{Inf}{}(\mathscr {N}))$ is strongly finitary. An inference rule is *strongly finitary* if the set of inferences it defines is strongly finitary. We can extend a strongly finitary function *F* to sets of base formulas by taking $F_{\textbf{F}}(N) = \lfloor F(N \times {{\mathscr {P}}}_\text {fin}({{\textbf {A}}})) \rfloor$ for every $N \subseteq {\textbf{F}}$ and to sets of timestamped A-formulas by taking  for every $\mathcal {N} \subseteq \textbf{TAF}$. The functions *F* and $F_{\textbf{F}}$ are finitary, mapping finite sets to finite sets. Moreover, if *F* and *G* are strongly finitary, then so is $\mathscr {N} \mapsto F(\mathscr {N}) \cup G(\mathscr {N})$.

The function *B* in Definition 69 gives a bound on the new assertions. For the inference rules Base, Unsat, Collect, Trim, and StrongUnsat, we can set $B(N) = \emptyset$ since the conclusions do not contain assertions which were not already in the premises. So if $\textit{FInf}{}(N)$ is finite for every finite $N \subseteq {\textbf{F}}$, then $\textit{SInf}{}$ is strongly finitary. The inference rules Split and Approx require a nonempty *B*(*N*),  containing the assertions chosen for the split A-formulas. Deterministic splitting rules (where the chosen assertions are fully determined by the base formula), such as AVATAR’s, are thus also strongly finitary because then *B*(*N*) is finite. The optional inference rule Tauto is not strongly finitary.

For the completeness of the given clause procedure, Lemma 77, we will fix a strongly finitary function *I* restricting the inferences: The prover may perform an inference only if its conclusion is in $I(\mathscr {A}_i)$, where $\mathscr {A}_i$ is the active clause set after the Infer transition. This restriction will allow us to rule out the case where $\lfloor \bigcup _i \mathscr {A}_i \rfloor$ is finite and the prover switches models without making progress. Condition (1) in Definition 69 then says that the prover only infers finitely many ${\textbf{F}}$-formulas—this will in turn ensure that splitting creates only finitely many new assertions. Condition (2) says that the inferred A-formulas contain only finitely many new assertions. Taken together, only finitely many assertions are added in the case, where $\lfloor \bigcup _i \mathscr {A}_i \rfloor$ is finite, which means that the prover can only switch models finitely often, a contradiction.

Simplification rules used by the prover must be restricted even more to ensure completeness, because they can lead to new splits and assertions, and hence switching to new models. For example, simplifying $\textsf{p}(x*0)\vee \textsf{p}(x)$ to $\textsf{p}(0) \vee \textsf{p}(x)$ transforms a nonsplittable clause into a splittable one. Even for the standard orders on first-order clauses, there can be infinitely many clauses that are smaller than a given clause. For example, with the lexicographic path order, the set  is typically infinite for a term *u*. If simplifications were to produce infinitely many new splittable clauses, the prover might split clauses and switch propositional interpretations forever without making progress.

#### Example 70

Even if $\prec$ is a well-founded order on ${\textbf{F}}$, and *I* is a set of binary inferences such that $C_2 \prec C_1$ and $D \prec C_1$ for every inference $(C_2,C_1,D) \in I$, simplification with *I* can still produce infinitely many base formulas. This is because in an ${{\textsf{A}}}{{\textsf{V}}}$ prover, the same base formula may be rederived infinitely often (for example due to switching between two families of interpretations).

As a slightly abstract example, consider ${\textbf{F}}= {\mathbb {N}} \cup \{\infty \}$ with $0 \prec 1 \prec 2 \prec \cdots \prec \infty$, and let  If the prover then rederives $\infty$ infinitely often, it might simplify $\infty$ using *I* in a different way each time, the first time to 1, then to 2, and so on. We hence need to ensure that, in the entire derivation, each formula is simplified in at most a finite number of ways.

#### Definition 71

Let $\prec$ be a transitive well-founded relation on ${\textbf{F}},$ and let $\preceq$ be its reflexive closure. A function $S: {\textbf {AF}}\rightarrow {\mathscr {P}}({\textbf {AF}})$ is a *strongly finitary simplification bound* for $\prec$ if $\mathscr {N} \mapsto \bigcup _{\mathscr {C} \in \mathscr {N}} S(\mathscr {C})$ is strongly finitary and $\lfloor \mathscr {C}' \rfloor \preceq \lfloor \mathscr {C} \rfloor$ for every $\mathscr {C}' \in S(\mathscr {C}).$

The prover may simplify an A-formula $\mathscr {C}$ to $\mathscr {C}'$ only if $\mathscr {C}' \in S(\mathscr {C})$. It may also delete $\mathscr {C}$. Strongly finitary simplification bounds are closed under unions, allowing the combination of simplification techniques based on $\prec$. For superposition, a natural choice for $\prec$ is the clause order. Analogously to strongly finitary functions, we define the extension of strongly finitary simplification bounds to sets of formulas as $S_{\textbf{F}}(N) = \lfloor S(N \times {{\mathscr {P}}}_\text {fin}({{\textbf {A}}})) \rfloor .$ The key property of strongly finitary simplification bounds is that if we saturate a finite set of A-formulas w.r.t. simplifications, the saturation is also finite. This is crucial to bound the number of A-formulas derived by the prover and thus the number of possible model switches: If the prover only selects a finite set of A-formulas for inferences, then simplification will only derive finitely many A-formulas as well, no matter how often an A-formula is derived again:

#### Lemma 72

Let *S* be a strongly finitary simplification bound. For every $\mathscr {C} \in {\textbf {AF}}$, let $S^*(\mathscr {C}) = \bigcup _{i=0}^\infty S^i(\mathscr {C})$, where $S^i$ denotes the *i*th iterate of *S*. Then $S^*$ is also a strongly finitary simplification bound.

#### Proof

Clearly, $\lfloor \mathscr {C}' \rfloor \preceq \lfloor \mathscr {C} \rfloor$ for every $\mathscr {C}' \in S^*(\mathscr {C}).$ Let $N \subseteq {\textbf{F}}$ be finite. Next we show that ${S^*}_{\textbf{F}}(N)$ is finite as well. Define a sequence of finite sets $M_i \subseteq {\textbf{F}}$ by $M_0 = N$ and $M_{i+1} = M_i \cup S_{\textbf{F}}(M_i).$ Clearly, $S_{\textbf{F}}(M_\infty ) \cup N \subseteq M_\infty = {S_{\textbf{F}}}^*(N) \supseteq {S^*}_{\textbf{F}}(N).$

To show that $M_\infty$ is finite, consider the sequence $M_{i+1}\setminus M_i$. By construction $S_{\textbf{F}}(M_i) {\setminus } M_i \subseteq (S_{\textbf{F}}(M_i {\setminus } M_{i-1}) \cup S_{\textbf{F}}(M_{i-1})) {\setminus } M_i \subseteq (S_{\textbf{F}}(M_i {\setminus } M_{i-1}) \cup M_i) {\setminus } M_i = S_{\textbf{F}}(M_i {\setminus } M_{i-1}) {\setminus } M_i$, and thus $M_{i+1}{\setminus } M_i = S_{\textbf{F}}(M_i{\setminus } M_{i-1}){\setminus } M_i$. Because *S* is a strongly finitary simplification bound, $M_{i+1}\setminus M_i$ is always finite, and decreasing in the multiset extension of $\prec$. It is even strictly decreasing as long as $M_{i+1}\setminus M_i\ne \emptyset$ because $M_{i+1}\setminus M_i\cap M_i\setminus M_{i-1}=\emptyset$ and therefore $M_{i+1}\setminus M_i\ne M_i\setminus M_{i+1}$. From the well-foundedness of $\prec$, it follows that $M_{i+1} {\setminus } M_i = \emptyset$ for large enough *i* and that ${S_{\textbf{F}}}^*(N) = M_\infty = \bigcup _i M_i = M_0 \cup \bigcup _i (M_{i+1}{\setminus } M_i)$ is finite.

Thus $\lfloor S^*(\mathscr {N}) \rfloor$ is finite whenever $\lfloor \mathscr {N} \rfloor$ is finite. It remains to exhibit a function $B': {\textbf{F}}\rightarrow {{\mathscr {P}}}_\text {fin}({{\textbf {A}}})$ such that $\lceil S^*(\mathscr {N}) \rceil \subseteq B'(\lfloor \mathscr {N} \rfloor ) \cup \lceil \mathscr {N} \rceil .$ By assumption, we have such a function *B* for *S*. Then set $B'(C) = B({S^*}_{\textbf{F}}(C))$ (which is finite for all *C*), and we have $\lceil S^{i+1}(\mathscr {N}) \rceil = \lceil S(S^i(\mathscr {N})) \rceil \subseteq B(\lfloor S^i(\mathscr {N}) \rfloor ) \cup \lceil S^i(\mathscr {N}) \rceil \subseteq B'(\lfloor \mathscr {N} \rfloor ) \cup \lceil S^i(\mathscr {N}) \rceil$ and therefore $\lceil S^i(\mathscr {N}) \rceil \subseteq \lceil \mathscr {N} \rceil \cup B'(\lfloor \mathscr {N} \rfloor )$ by induction and hence $\lceil S^*(\mathscr {N}) \rceil \subseteq \lceil \mathscr {N} \rceil \cup B'(\lfloor \mathscr {N} \rfloor ).$ $\square$

#### Example 73

Let ${\textbf{F}}$ be the set of first-order clauses and $S(C \mathbin {\leftarrow }A) = \{ C' \mathbin {\leftarrow }A' | C'$ is a subclause of *C* and $A' \subseteq A\}.$ Then *S* is a strongly finitary simplification bound, because (1) $C' \preceq C$ if $C'$ is a subclause of *C* and (2) each clause has only finitely many subclauses. This *S* covers many simplification techniques, including elimination of duplicate literals (simplifying $C \vee L \vee L$ to $C \vee L$), deletion of resolved literals (simplifying $C \vee u \not \approx u$ to *C*), and subsumption resolution (simplifying $C\sigma \vee D\sigma \vee L\sigma$ to $C\sigma \vee D\sigma$ given the side premise $C \vee \lnot L$). Removing redundant clauses is possible with every *S*.

#### Example 74

If the Knuth–Bendix order [18] is used as the term order and all weights are positive, then  is a strongly finitary simplification bound. This can be used to cover demodulation.

#### Example 75

The simplification rules Collect, Trim, and StrongUnsat from Sect. 3.1 are all strongly finitary simplification bounds. In a practical implementation, Split will deterministically split $C \in {\textbf{F}}$ into $C_1, \dots , C_n$ and use the same assertions $a_i \in \textit{asn}(C_i)$ every time. Under these conditions, Split is also a strongly finitary simplification bound.

For other term orders, the *S* in Example 74 is not strongly finitary, and proving that demodulation is a strongly finitary simplification bound is much more involved. In this case, the necessary strongly finitary simplification bound even depends on the derivation.

#### Example 76

If unit equations are only removed by demodulation, reflexivity deletion, or subsumption, the one-step demodulations possible at any point in the derivation are a strongly finitary simplification bound. By Lemma 72, this implies that many-step demodulation is also a strongly finitary simplification bound.

Assume that demodulation is performed in a postorder traversal (i.e., subterms first), always rewriting using the oldest available equation. Also assume that if $l\approx r$ is an existing (ordered) equation and the prover derives $l\approx r'$, that $l\approx r'$ is not used for demodulation (but for example instead simplified to $r\approx r'$).

We will show that for every term *t*, there exist only finitely many terms $t'$ that are simplified from *t* in one step. The term $t'$ will typically be different over the course of the derivation. However, we can assign a decreasing well-founded measure to the rewrite step, ensuring finiteness. Consider a demodulation step transforming $C[l\sigma ]$ to $C[r\sigma ]$ using an equation $l \approx r$, with $r\sigma \prec l\sigma$. Let *i* be the index of $l\sigma$ in $C[l\sigma ]$ in a postorder traversal, let |*l*| be the number of nonvariable positions in *l*, and let $u = 1$ if the equation is unorientable ($l \not \succ r$) and $u = 0$ otherwise. Then the tuple $(i, |l|, u, r\sigma )$ decreases or stays the same in the left-to-right lexicographic order as we move along the derivation.

If the prover derives a new ordered equation $l' \approx r',$ it is possible that it applies at an earlier position in *C*, thus decreasing the *i*. Otherwise, it applies at the same position as $l \approx r$ previously, and the prover rewrites using the older $l \approx r$ first, keeping the tuple unchanged. If the equation $l\approx r$ is subsumed by $l'\approx r'$ and deleted, then $|l'| < |l|.$ (Note that if $l\approx r$ is subsumed by $l\approx r'$, then *r* and $r'$ are identical because all variables that occur in $r,r'$ also occur in *l*.) If $l\approx r$ is simplified to $t\approx r$ by $l'\approx r'$ and $l \not \approx l'$, then $|l'| < |l|$. If $l\approx r$ is simplified to $t\approx r$ by $l\approx r'$ and $l\approx r$ is unorientable, then $|l'| = |l|$ and *u* decreases. If $l\approx r$ is simplified to $l\approx t$ by $l'\approx r'$, then |*l*| stays the same, *u* might decrease, and $r\sigma \succ t\sigma$.

Based on the above definitions, we introduce a fairness criterion that is more concrete and easier to apply than the definition of fairness of $\Longrightarrow _{{{\textsf{A}}}{{\textsf{V}}}}$-derivations.

#### Lemma 77

Let *I* be a strongly finitary function, and let *S* be a strongly finitary simplification bound. Then a well-timestamped $\Longrightarrow _{{{\textsf{A}}}{{\textsf{V}}}}$-derivation $(\mathcal {J}_i, \mathcal {A}_i, \mathcal {P}_i,\mathcal {Q}_i, \mathcal {L}_i)_i$ is fair if all of the following conditions hold:

is a selection order on $\bigcup _i \mathcal {P}_i$, and  is a selection order on ${\textbf{F}}$;
2. $\mathcal {A}_0 = \emptyset ,$ $\mathcal {L}_0 = \emptyset ,$ and $\mathcal {P}_0 \cup \mathcal {Q}_0$ is finite;
3. for every Infer transition, either $\mathcal {C}$ is -minimal in $\mathcal {P}$ or $\lfloor \mathcal {C} \rfloor$ is -minimal in $\lfloor \mathcal {P} \rfloor$;
4. for every Infer transition, $\mathcal {P}' \cup \mathcal {Q}' \subseteq I_\textbf{TAF}(\mathcal {A} \cup \{\mathcal {C}\})$;
5. for every Process transition, ;
6. if $\mathcal J_i \not \models (\mathcal {Q}_i)_\bot$, then eventually Switch or StrongUnsat occurs;
7. if $\mathcal {P}_i \ne \emptyset$, then eventually Infer, Switch, or StrongUnsat occurs;
8. there are infinitely many indices *i* such that either $\mathcal {P}_i = \emptyset$ or Infer chooses a -minimal $\mathcal {C}$ at *i*;
for every subsequence $(\mathcal J'_j)_j$ of $(\mathcal J_i)_i$ converging to a limit point $\mathcal J.$

#### Proof

If  the derivation is trivially fair. Otherwise, the StrongUnsat transition never occurs, and therefore Switch is eventually applied if the propositional clauses are not satisfied by the interpretation. Hence $\mathcal J_i \models \mathcal {Q}_i$ for infinitely many *i*,  thus satisfying condition (C)(2) of Definition 36. Conditions (A) and (B) are satisfied due to condition (2) of this lemma, and (C)(4) due to (9). It remains to construct a subsequence $(\mathcal J'_j, \mathcal {A}'_j, \mathcal {P}'_j, \mathcal {Q}'_j, \mathcal {L}'_j)_j$ such that $(\mathcal J'_j)_j$ converges to a limit point and $\liminf _{j\rightarrow \infty } \textit{TSInf}{}(\mathcal {A}'_j, \mathcal {P}'_j) = \emptyset ,$ as required for (C)(3).

Case 1:The set of -minimal A-formulas selected in an Infer transition for some state *j* is unbounded in . That is, for every $C \in {\textbf{F}}$, there is a Infer transition from state *j* such that the selected A-formula $\mathcal {S}_j$ is -minimal in $\mathcal {P}_j$, and . These Infer transitions clearly form an infinite subsequence. By Lemma 35, we can further refine it into a subsequence $(\mathcal J'_j, \mathcal {A}'_j, \mathcal {P}'_j, \mathcal {Q}'_j, \mathcal {L}'_j)_j$, where $(\mathcal J'_j)_j$ converges to a limit point. Assume towards a contradiction that $\iota \in \liminf _{j\rightarrow \infty } \textit{TSInf}{}(\mathcal {A}'_j, \mathcal {P}'_j)$. By Lemma 68, for every $\mathcal {C} \in \textit{prems}(\iota )$ there exists an index *j* such that . Therefore $\textit{prems}(\iota ) \subseteq \mathcal {A}'_j$ by the -minimality requirement on the Infer transition, a contradiction.

Case 2:The set of -minimal A-formulas selected in an Infer transition for some state *j* is unbounded in . This case is analogous to case 1.

Case 3:Neither case 1 nor case 2 apply. Then the set of -minimal formulas selected in an Infer transitions is bounded and hence finite since  is a selection order. Similarly, the set of -minimal $\textbf{TAF}$-formulas selected in an Infer transitions is finite as well. Let $\mathcal {T}$ be the set of A-formulas selected in an Infer transition. So $\lfloor \mathcal {T} \rfloor$ and therefore $\bigcup _i \lfloor \mathcal {A}_i \rfloor$ are both finite. The set $S^*_{\textbf{F}}(I_{\textbf{F}}(\bigcup _i \lfloor \mathcal {A}_i \rfloor ) \cup \lfloor \mathcal {P}_0 \rfloor \cup \lfloor \mathcal {Q}_0 \rfloor )$ is then finite, and therefore  is finite as well. Since both $S^*$ and *I* are strongly finitary, only a finite number of new assertions are introduced, and  is also finite. Thus $(\bigcup _i \mathcal {Q}_i)_\bot$ is also finite, and only a finite number of Switch transitions can occur. Thus there exists an index *N* such that no Switch transitions occur at states $i > N$.

Now take the whole derivation as subsequence. We have $\mathcal {P}_\infty = \emptyset$ because there are infinitely many states *j* with a Infer transition such that a -minimal A-formula $\mathcal {S}_j$ is selected. After the initial *N* steps, every A-formula is selected only once (that is, $\mathcal {S}_i \ne \mathcal {S}_j$ if $i \ne j$), because once it has been selected, it can only be removed from the active set if it becomes redundant or locked. (There are no Switch transitions.) In either case, the A-formula is removed from the passive set for the rest of the derivation. Newly derived A-formulas have a different timestamp due to the well-timestampedness requirement. Therefore, once an A-formula is in the active set, it will not come back into the passive set, and we have $\liminf _{i\rightarrow \infty } \textit{TSInf}{}(\mathcal {A}_i, \mathcal {P}_i) = \emptyset .$ $\square$

Recall the abstract counterexample from Sect. 6.2 in which the A-formulas $\mathcal {C}$ and $\mathcal {D}$ were selected and disabled in turn. Intuitively, selection orders, together with the restrictions on the inferences, ensure that the prover will follow roughly the same steps whenever it is in a model that enables $\mathcal {C}$ and $\mathcal {D}$. Since there are only finitely many formulas that it can select for inferences before $\mathcal {C}$ or $\mathcal {D}$, the prover will eventually repeat itself and thus make progress.

We could refine ${{\textsf{A}}}{{\textsf{V}}}$ further and use Lemma 77 to show the completeness of an imperative procedure such as Voronkov’s extended Otter loop [28, Fig. 3], thus showing that AVATAR as implemented in Vampire is complete if locking is sufficiently restricted. A slight complication is that in Vampire’s AVATAR, A-clauses $C \mathbin {\leftarrow }\{[C]\}$ are generated on a per-need basis when switching model. This is not a serious issue because we can imagine that the A-clauses were there all along in the $\mathcal {Q}$ set.

Even the concrete criterion offered by Lemma 77 refers, in its condition 9, to limit superiors and limit points. Some architectures will satisfy it by their very design. For AVATAR, an easy way to meet the condition is to bound the number of times each A-formula can be locked. Once that number has been reached, the A-formula can no longer be locked. An alternative, suggested by a reviewer, is to disable all splitting after the prover has run for a specified time.

## Application to Other Architectures

AVATAR may be the most natural application of our framework, but it is not the only one. We will complete the picture below by studying splitting without backtracking, labeled splitting, and SMT with quantifiers.

### Splitting Without Backtracking

Before the invention of AVATAR, Riazanov and Voronkov [25] had already experimented with splitting in Vampire in a lighter variant without backtracking. They based their work on ordered resolution $\textsf {O}$ with selection [2], but the same ideas work with superposition. Weidenbach [31, end of Sect. 4.5] independently outlined the same technique at about the same time.

The basic idea of splitting without backtracking is to extend the signature ${\Sigma }$ with a countable set ${\mathbb {P}}$ of nullary predicate symbols disjoint from ${\Sigma }$ and to augment the base calculus with a binary splitting rule that replaces a clause $C \vee D$ with $C \vee \textsf{p}$ and $D \vee \lnot \textsf{p}$, where *C* and *D* share no variables and $\textsf{p} \in {\mathbb {P}}$. Riazanov and Voronkov require that the precedence $\prec$ makes all ${\mathbb {P}}$-literals smaller than the ${\Sigma }$-literals. Binary splitting then qualifies as a simplification rule. They show that their rule and a few variants are consistency-preserving. They do not show refutational completeness, but this is obvious since the rule is a simplification.

Riazanov and Voronkov also extend the selection function of the base calculus to support ${\mathbb {P}}$-literals. They present two such extensions: The *blocking* function allows for the selection of ${\mathbb {P}}$-literals in clauses that contain ${\Sigma }$-literals, whereas the *parallel* function selects only maximal ${\mathbb {P}}$-literals in pure ${\mathbb {P}}$-clauses and otherwise imitates the original selection function. Parallel selection cleanly separates the ${\mathbb {P}}$- and the ${\Sigma }$-literals. Bachmair and Ganzinger proved O statically complete, and this also obviously extends to ordered resolution with this extension, which we denote by ${\textsf{O}}_{\mathbb {P}}$, since it is an instance of the same calculus.

The calculus ${\textsf{O}}_{\mathbb {P}}{}$ is closely related to an instance of our framework. Let ${\textbf{F}}$ be the set of ${\Sigma }$-clauses, with the empty clause as $\bot$. Let $\textsf {O}=(\textit{FInf}{},\textit{FRed})$, where $\textit{FInf}{}$ is the set of ordered resolution inferences on ${\textbf{F}}$ with some selection function and $\textit{FRed}$ is the standard redundancy criterion [2, Sect. 4.2.2], and similarly ${\textsf{O}}_{\mathbb {P}}=(\textit{FPInf}{},\textit{FPRed})$. We use the notion of entailment from Example 1 for the base relations $\models$ and $|\!\!\!\approx$ for both calculi. We take ${{\textbf {V}}}={\mathbb {P}}$ for defining ${\textbf {AF}}$. The properties (D1)–(D6) and (R1)–(R7) are verified for $\models$ and $\textit{FRed}$, respectively. This gives us a splitting calculus ${{\textsf{L}}}{{\textsf{A}}}= (\textit{SInf}{},\textit{SRed})$, whose name stands for *lightweight AVATAR.* Lightweight AVATAR amounts to the splitting architecture Cruanes implemented in Zipperposition and confusingly called “AVATAR” [9, Sect. 2.5]. Binary splitting can be realized in ${{\textsf{L}}}{{\textsf{A}}}$ as the following simplification rule:
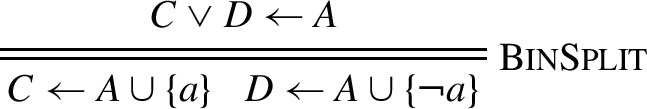


with the side conditions that $a \in \textit{asn}(C)$ and $C \vee D$ is splittable into *C*, *D*. By Theorem 21, ${{\textsf{L}}}{{\textsf{A}}}$ is complete.

Like splitting without backtracking but unlike the real AVATAR, Cruanes’s architecture is not guided by a propositional model. It is essentially an instance of ${{\textsf{L}}}{{\textsf{A}}}$, except that it is based on superposition instead of ordered resolution. It performs branch-specific simplifications (a special case of subsumption demodulation [17]), which is supported by our locking mechanism. A SAT solver is used to detect propositional unsatisfiability (corresponding to our Unsat rule) and to eliminate assertions that are implied at the SAT solver’s top level (corresponding to our Trim rule).

The calculi ${\textsf{O}}_{\mathbb {P}}$ and ${{\textsf{L}}}{{\textsf{A}}}$ are very similar but not identical. ${\textsf{O}}_{\mathbb {P}}$ has a slightly stronger notion of inference redundancy, because its order $\prec$ can access not only the ${\Sigma }$-literals but also the ${\mathbb {P}}$-literals, whereas with ${{\textsf{L}}}{{\textsf{A}}}$ the ${\mathbb {P}}$-literals are invisible to the base calculus. To see this, consider the set consisting of the ${\Sigma }_{\mathbb {P}}$-clauseswhere ${\mathbb {P}} = \{\textsf{a}, \textsf{b}\}$. Given the precedence $\textsf{a} \prec \textsf{b} \prec \textsf{p} \prec \textsf{q}$, an ordered resolution inference is possible between the first two clauses, with $\textsf{b} \vee \textsf{p}$ as its conclusion. This inference is redundant according to $\textit{FPRed}_\text {I}$, because the conclusion is entailed by the first, third, and fourth clauses taken together, all of which are $\prec$-smaller than the main premise $\textsf{b} \vee \textsf{p} \vee \lnot \textsf{q}$. However, the corresponding ${\textbf {AF}}$-inference is not redundant according to $\textit{SRed}_\text {I}$, because the assertions are simply truncated by the projection operator $(\,)_{\mathcal {J}}$ and not compared. Without the assertions, the third and fourth clauses are equal to, but not smaller than, the main premise, and the inference is not redundant. Note that the set is not saturated: Inferences are possible to derive $\lnot \textsf{a} \vee \textsf{p}$ and $\textsf{a} \vee \textsf{p}$, which make $\textsf{b} \vee \textsf{p}$ redundant.

Another dissimilarity is that ${{\textsf{L}}}{{\textsf{A}}}$ can detect unsatisfiability immediately using a SAT solver, whereas splitting without backtracking generally needs many propositional resolution steps to achieve the same. Correspondingly, on satisfiable problems, ${{\textsf{L}}}{{\textsf{A}}}$ allows smaller saturated sets. For example, while the A-clause set $\{\bot \mathbin {\leftarrow }\{\textsf{a}, \lnot \textsf{b}\}{,}\> \bot \mathbin {\leftarrow }\{\textsf{b}\}\}$ is saturated, its ${\textsf{O}}_{\mathbb {P}}$ counterpart is subject to an inference between $\lnot \textsf{a} \vee \textsf{b}$ and $\lnot \textsf{b}$.

As positive results, we will show that ${\textsf{O}}_{\mathbb {P}}$ and ${{\textsf{L}}}{{\textsf{A}}}$ share the same notion of entailment and ${\textsf{O}}_{\mathbb {P}}$’s redundancy criterion is stronger than ${{\textsf{L}}}{{\textsf{A}}}$’s, yet saturation w.r.t. ${{\textsf{L}}}{{\textsf{A}}}$ guarantees saturation w.r.t. ${\textsf{O}}_{\mathbb {P}}$, up to the natural correspondence between A-clauses and ${\Sigma }_{\mathbb {P}}$-clauses. More precisely, a ${\Sigma }_{\mathbb {P}}$-clause can be written as $C \vee L_1 \vee \cdots \vee L_n$, where *C* is a ${\Sigma }$-clause and the $L_i$’s are ${\mathbb {P}}$-literals. Let $\alpha$ be a bijective mapping such that $\alpha (C\vee L_1 \vee \cdots \vee L_n) = C\mathbin {\leftarrow }\{\lnot L_1, \dots , \lnot L_n\}$ is the corresponding A-clause. We overload the operator $\lfloor \, \rfloor$ to erase the ${\mathbb {P}}$-literals: $\lfloor C \vee L_1 \vee \cdots \vee L_n \rfloor = \lfloor C\mathbin {\leftarrow }\{\lnot L_1, \dots , \lnot L_n\} \rfloor = C$. Moreover, let ${\mathscr {G}}$ denote the function that returns all ground instances of a clause, clause set, or inference according to $\Sigma$, which is assumed to contain at least one constant.

#### Lemma 78

Given the ${\Sigma }_{\mathbb {P}}$-clause sets *M*, *N*, we have $M \models N$ if and only if $\alpha (M) \models \alpha (N)$.

#### Proof

Since both entailments are defined via grounding, it suffices to consider the case where *M* and *N* are ground.

For the forward direction, we must show that $(\alpha (M))_{\mathcal {J}} \models \lfloor {N} \rfloor$ for some $\mathcal {J}$ in which $\alpha (N)$ is enabled. Let ${\mathcal {K}}$ be a ${\Sigma }$-model of $\{\alpha (M)\}_{\mathcal {J}}$. We will show that at least one clause in $\lfloor {N} \rfloor$ is true in ${\mathcal {K}}$. We start by showing that ${\mathcal {K}}\cup \mathcal {J}$ is a ${\Sigma }_{\mathbb {P}}$-model of *M*. Let $C \in M$. If $\alpha (C)$ is enabled in $\mathcal {J}$, then $\lfloor {C} \rfloor \in (\alpha (M))_\mathcal {J}$. Thus ${\mathcal {K}}\models \lfloor {C} \rfloor$ and finally ${\mathcal {K}}\cup \mathcal {J} \models \{C\}$. Otherwise, $\alpha (C)$ contains an assertion that is false in $\mathcal {J}$, which means that *C* contains the complementary ${\mathbb {P}}$-literal, which is true in $\mathcal {J}$, and we have ${\mathcal {K}}\cup \mathcal {J} \models \{C\}$. Either way, ${\mathcal {K}}\cup \mathcal {J} \models M$ and hence, since $M\models N$, one of the clauses in *N* is true in ${\mathcal {K}}\cup \mathcal {J}$. Since $\alpha (N)$ is enabled in $\mathcal {J}$, all ${\mathbb {P}}$-literals occurring in *N* are false in ${\mathcal {K}}\cup \mathcal {J}$. Therefore, each clause in *N* must contain a true ${\Sigma }$-literal in ${\mathcal {K}}$, which means that the corresponding clause in $\lfloor {N} \rfloor$ must also be true in ${\mathcal {K}}$.

For the backward direction, we must show that $M \models N$. Let ${\mathcal {K}}\cup \mathcal {J}$ be a ${\Sigma }_{\mathbb {P}}$-model of *M*, where ${\mathcal {K}}$ is a ${\Sigma }$-interpretation and $\mathcal {J}$ is a ${\mathbb {P}}$-interpretation. We will show that a clause in *N* is true in ${\mathcal {K}}\cup \mathcal {J}$. If $\alpha (N)$ is disabled in $\mathcal {J}$, there exists a ${\mathbb {P}}$-literal in some clause from *N* that is true in ${\mathcal {K}}\cup \mathcal {J}$, which suffices to make the entire clause true. Otherwise, *N* is enabled in $\mathcal {J}$ and then $(\alpha (M))_{\mathcal {J}} \models \lfloor {N} \rfloor$. Since ${\mathcal {K}}\cup \mathcal {J} \models M$, we have ${\mathcal {K}}\models (\alpha (M))_{\mathcal {J}}$. Hence, one of the clauses in $\lfloor {N} \rfloor$ is true in ${\mathcal {K}}$, and its counterpart in *N* is also true in ${\mathcal {K}}\cup \mathcal {J}$. $\square$

#### Lemma 79

Given an inference $\iota$ over ${\Sigma }_{\mathbb {P}}$-clauses, if $\alpha (\iota )$ is a Base inference, then $\iota \in \textit{FPInf}{}$.

#### Proof

Let $\iota = (C_n,\dots ,C_1,C_0)$ and assume $\alpha (\iota )$ is a Base inference. By the definition of ordered resolution, none of the ${\Sigma }$-clauses $\lfloor {C_i} \rfloor$, for $i\in \{1,\dots ,n\}$, can be $\bot$. Thus, the selected literals in the premises coincide with those chosen by the parallel selection function on the ${\Sigma }_{\mathbb {P}}$-clauses $C_i$ and so $\iota \in \textit{FPInf}{}$. $\square$

#### Lemma 80

(1) Given a ${\Sigma }_{\mathbb {P}}$-clause *C*, if $\alpha (C) \in \textit{SRed}_\text {F}(\alpha (N))$, then $C \in \textit{FPRed}_\text {F}(N)$. (2) Given an inference $\iota$ over ${\Sigma }_{\mathbb {P}}$-clauses, if $\alpha (\iota ) \in \textit{SRed}_\text {I}(\alpha (N))$, then $\iota \in \textit{FPRed}_\text {I}(N)$.

#### Proof

For (2), let $\iota ' \in {\mathscr {G}}(\iota )$ and let $C_n,\dotsc ,C_1$ be $\iota '$’s premises and $C_0$ be its conclusion. Let  and . By the definition of standard redundancy, assuming that $\{\alpha (C_n),\dots ,\alpha (C_2)\} \cup {\mathscr {E}}\models \{\alpha (C_0)\}$ we need to show that $\{C_n,\dots ,C_2\} \cup {\mathscr {F}}\models \{C_0\}$. By Lemma 78, this amounts to showing that $\{\alpha (C_n),\dots ,\alpha (C_2)\} \cup \alpha ({\mathscr {F}}) \models \{\alpha (C_0)\}$. By (D3), this follows from our assumption if ${\mathscr {E}}\subseteq \alpha ({\mathscr {F}})$. This subset inclusion holds because if $C \prec \lfloor {C_1} \rfloor$, then we have $C \vee D \prec C_1$ for every ${\mathbb {P}}$-clause *D*, since ${\mathbb {P}}$-literals are smaller than ${\Sigma }$-literals.

For (1), essentially the same line of argumentation applies, with $n = 1$ and $C_1 = C_0 = C$. $\square$

#### Lemma 81

(Saturation)  Let *N* be a set of ${\Sigma }_{\mathbb {P}}$-clauses. If *N* is saturated w.r.t. ${\textsf {O}}_\mathrm {\mathbb {P}}$, then $\alpha (N)$ is saturated w.r.t. LA.

#### Proof

Let $\iota = (\alpha (C_n),\dots ,\alpha (C_1),\alpha (C_0))\in \textit{SInf}{}$ be an inference with premises in $\alpha (N)$. We will show that it is redundant w.r.t. $\alpha (N)$.

Case Base:We need to show that $\{\iota \}_{\mathcal {J}}\subseteq \textit{FRed}_\text {I}((\alpha ({\mathscr {G}}(N)))_\mathcal {J})$ for every propositional interpretations $\mathcal {J}$. The case where $\iota$ is disabled in $\mathcal {J}$ is trivial. Otherwise, let $\theta$ be a substitution such that $\iota \theta \in {\mathscr {G}}(\iota )$. We must show that $\{\lfloor {C_n}\theta \rfloor ,\dots ,\lfloor {C_2\theta } \rfloor \} \cup \lfloor {\mathscr {E}} \rfloor \models \{\lfloor {C_0}\theta \rfloor \}$, where . Because the premises’ assertions are contained in the conclusion’s, this is equivalent to showing that $\{{\alpha (C_n\theta )},\dots ,{\alpha (C_2\theta )}\} \cup {\mathscr {E}}\models \{{\alpha (C_0\theta )}\}$.

By Lemma 79, there exists an inference $(C_n,\dots ,C_1,C_0)\in \textit{FPInf}{}$. Since *N* is saturated, the inference is redundant—i.e., $\{C_n\theta ,\dots ,C_2\theta \} \cup {\mathscr {F}}\models \{C_0\theta \}$, where . If $\alpha ({\mathscr {F}}) \subseteq {\mathscr {E}}$, we can invoke Lemma 78 to conclude. However, in the general case, we have only that $\alpha ({\mathscr {F}}\setminus {\mathscr {F}}_\text {eq})\subseteq {\mathscr {E}}$, where , and thus there might be models of ${\mathscr {E}}$ that are models of ${\mathscr {F}}\setminus {\mathscr {F}}_\text {eq}$ but not of ${\mathscr {F}}_\text {eq}$. Fortunately, we can show that $\{C_n\theta ,\dots ,C_2\theta \}\cup ({\mathscr {F}}{\setminus }{\mathscr {F}}_\text {eq}) \models \{C_0\theta \}$. We proceed by removing from ${\mathscr {F}}$ each clause $D \in {\mathscr {F}}_\text {eq}$ in turn and by showing that the entailment is preserved by each step. Finally, we invoke Lemma 78. A slight complication is that ${\mathscr {F}}_\text {eq}$ may be infinite. However, by compactness, only a finite subset ${\mathscr {F}}'_\text {eq} \subseteq {\mathscr {F}}_\text {eq}$ is needed to have the desired entailment.

Let $D \in N$ be a clause that generalizes the ground, $\succ$-largest clause in ${\mathscr {F}}'_\text {eq}$. Then there exists an inference $(C_n,\dots ,C_2, D, D_0) \in \textit{FPInf}{}$ such that $\lfloor {C_0}\theta \rfloor \in \lfloor {\mathscr {G}}(D_0) \rfloor$ and the ${\mathbb {P}}$-literals of $D_0$ are the union of those of $C_n,\dots ,C_2, D$. By renaming the variables in *D* and $D_0$, we can ensure that $\lfloor {D}\theta \rfloor = \lfloor {C_1}\theta \rfloor$ and $\lfloor D_0\theta \rfloor = \lfloor {C_0}\theta \rfloor$. Now, to prove the desired entailment, assume that ${\mathcal {J}}$ is a model of $\{C_n\theta ,\dots ,C_2\theta \}\cup ({\mathscr {F}}\setminus \{D\theta \})$. Since *N* is saturated,

. Since we are proceeding from largest to smallest clause, we have , even if some clauses have been removed from ${\mathscr {F}}$ already. Thus, in both cases, ${\mathcal {J}}\models \{D_0\theta \}$. If ${\mathcal {J}}$ makes a ${\Sigma }$-literal of $D_0\theta$ true, ${\mathcal {J}}$ makes the same literal in $C_0\theta$ true. Otherwise, either ${\mathcal {J}}$ makes one of the ${\mathbb {P}}$-literals of $C_n\theta ,\dots ,C_2\theta$ true, satisfying $C_0\theta$ for the same reason, or it makes one of the ${\mathbb {P}}$-literals of *D* true and then ${\mathcal {J}}\models \{C_n\theta ,\dots ,C_2\theta \}\cup {\mathscr {F}}$, which as noted above implies ${\mathcal {J}}\models \{C_0\theta \}$ by the saturation of *N*. In both cases, ${\mathcal {J}}\models \{C_0\theta \}$.

Case Unsat:The inference derives $\bot$ from a set of ${\mathbb {P}}$-clauses $(\alpha (A_i))_{i=1}^n$ such that $\{\alpha (A_i)\}_i$ is propositionally unsatisfiable—i.e., $\{A_i\}_i\models \{\bot \}$ in ${\textsf{O}}_{\mathbb {P}}$. Since ${\textsf{O}}_{\mathbb {P}}$ is complete and $N\supseteq \{A_i\}_i$ is saturated, we have $\bot \in N$ and hence $\bot \in \alpha (N)$. Therefore, $\iota$ is redundant also in this case. $\square$

### Labeled Splitting

Labeled splitting, as originally described by Fietzke and Weidenbach [15] and implemented in SPASS, is a first-order resolution-based calculus with binary splitting that traverses the split tree in a depth-first way, using an elaborate backtracking mechanism inspired by CDCL [20]. It works on pairs $({\Psi },\mathscr {N})$, where ${\Psi }$ is a stack storing the current state of the split tree and $\mathscr {N}$ is a set of *labeled clauses*—clauses annotated with finite sets of natural numbers.

We model labeled splitting as an instance of the locking prover ${\textsf{L}}$ based on the splitting calculus ${{\textsf{L}}}{{\textsf{S}}}=(\textit{SInf}{},\textit{SRed})$ induced by the resolution calculus $\textsf {R}= (\textit{FInf}{},\textit{FRed})$, where $\models$ and $|\!\!\!\approx$ are as in Example 1 and ${{\textbf {V}}}= \bigcup _{i\in {\mathbb {N}}} \{\textsf{l}_i, \textsf{r}_i, \textsf{s}_i\}$. A-clauses are essentially the same as labeled clauses.

Splits are identified by unique *split levels*. Given a split on $C \vee D$ with level *k*, the propositional variables $\textsf{l}_k \in \textit{asn}(C)$ and $\textsf{r}_k \in \textit{asn}(D)$ represent the left and right branches, respectively. In practice, the prover would dynamically extend $\textit{fml}$ to ensure that $\textit{fml}(\textsf{l}_k) = C$ and $\textit{fml}(\textsf{r}_k) = D.$

When splitting, if we simply added the propositional clause $\bot \mathbin {\leftarrow }\{\lnot \textsf{l}_k, \lnot \textsf{r}_k\},$ we would always need to consider either $C \mathbin {\leftarrow }\{\textsf{l}_k\}$ or $D \mathbin {\leftarrow }\{\textsf{r}_k\},$ depending on the interpretation. However, labeled splitting can undo splits when backtracking. Yet fairness would require us to perform inferences with either *C* or *D*, which Fietzke and Weidenbach avoid. We solve this as follows. Let $\top = {{\sim }\bot }$. We introduce the propositional variable $\textsf{s}_k \in \textit{asn}(\top )$ so that we can enable or disable the split as we wish. The StrongUnsat rule then knows that $\textsf{s}_k$ is true and that the cases are exhaustive, but we can still switch to propositional models that disable both *C* and *D*. A-clauses are then split using the following binary variant of Split:
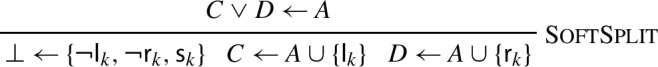


where *C* and *D* share no variables and *k* is the next split level. Unlike AVATAR, labeled splitting keeps the premise and might split it again with another level. We rely on locking to ensure that the premise is not split within either branch.

To emulate the original, the locking prover based on the ${{\textsf{L}}}{{\textsf{S}}}$ calculus must repeatedly apply the following three steps in any order until saturation:

1. Apply Base (via Lift and Derive) to perform an inference from the enabled A-clauses. If an enabled $\bot \mathbin {\leftarrow }A$ is derived with $A\subseteq \bigcup _i \{\textsf{l}_i, \textsf{r}_i\}$, apply Switch or StrongUnsat.
2. Use Derive (via Lift) to delete a redundant enabled A-clause.
3. Use Lock to temporarily remove a locally redundant enabled A-clause.
4. Use Derive (via Lift) to simplify an enabled A-clause. If the original A-clause is made redundant, delete it; otherwise, use Lock to temporarily remove it. If an enabled $\bot \mathbin {\leftarrow }A$ is derived with $A\subseteq \bigcup _i \{\textsf{l}_i, \textsf{r}_i\}$, apply Switch or StrongUnsat.
5. Apply SoftSplit (via Lift and Derive) with split level *k* on an A-clause $\mathscr {C}$. Then use Switch to enable the left branch and apply Lock on $\mathscr {C}$ with $\textsf{s}_k$ as the lock.

Disabled A-clauses are the ones that occur in branches other than the current one and in disabled splits. As such, they should not be used when exploring the current branch.

Switch is powerful enough to support all of Fietzke and Weidenbach’s backtracking rules, but to explore the tree in the same order as they do, we must choose the new model carefully. If a left branch is closed, the model must be updated so as to disable the splits that were not used to close this branch and to enable the right branch. If a right branch is closed, the split must be disabled, and the model must switch to the right branch of the closest enabled split above it with an enabled left branch. If a right branch is closed but there is no split above with an enabled left branch, the entire tree has been visited. Then, a propositional clause $\bot \mathbin {\leftarrow }A$ with $A\subseteq \bigcup _i \{\textsf{s}_i\}$ is $\models$-entailed by the A-clause set, and StrongUnsat can finish the refutation by exploiting $\textit{fml}(\textsf{s}_i) = \top$.

We illustrate the strategy on an example.

#### Example 82

Let ${{\mathscr {N}}}_0$ be the clause set

$$\begin{aligned}\begin{aligned} \{&\lnot \textsf{q}(x){,}{} & {} \textsf{p}(x)\vee \textsf{q}(y){,}{} & {} \textsf{r}(x)\vee \textsf{s}(y){,}{} & {} \lnot \textsf{p}(x)\vee \textsf{q}(y)\} \end{aligned}\end{aligned}$$

It is unsatisfiable due to the first, second, and fourth clauses. Let $\mathcal {J}_0=\lnot {{\textbf {V}}}$ be the initial model. The first disjunction is split into $\textsf{p}(x)\mathbin {\leftarrow }\{\textsf{l}_0\}$, $\textsf{q}(y)\mathbin {\leftarrow }\{\textsf{r}_0\}$ and $\bot \mathbin {\leftarrow }\{\lnot \textsf{l}_0,\lnot \textsf{r}_0,\textsf{s}_0\}$ by SoftSplit. Then a Switch transition replaces $\mathcal {J}_0$ with $(\mathcal {J}_0{\setminus }\{\lnot \textsf{l}_0,\lnot \textsf{s}_0\}) \cup \{\textsf{l}_0,\textsf{s}_0\}$. This enables the A-clause $\textsf{p}(x)\mathbin {\leftarrow }\{\textsf{l}_0\}$. Then Lock removes $\textsf{p}(x)\vee \textsf{q}(y)$ from $\mathscr {N}_0$ for as long as $\textsf{s}_0$ is enabled. Splitting the other two disjunctions leads to the state $(\mathcal J', \mathscr {N}', \mathscr {L}')$, wherewhere the last three clauses listed in $\mathscr {N}'$ are disabled and thus currently unusable for inferences.

The first backtracking step happens after a Base inference produces $\bot \mathbin {\leftarrow }\{\textsf{l}_0,\textsf{l}_2\}$ from $\textsf{p}(x)\mathbin {\leftarrow }\{\textsf{l}_0\}$ and $\lnot \textsf{p}(x)\mathbin {\leftarrow }\{\textsf{l}_2\}$. The Switch disables $\textsf{s}_1$, because this split was not useful in closing the branch, and it moves from branch $\textsf{l}_2$ to $\textsf{r}_2$. The new model disables $\lnot \textsf{p}(x)\mathbin {\leftarrow }\{\textsf{l}_2\}$, enables $\textsf{q}(y)\mathbin {\leftarrow }\{\textsf{r}_2\}$, and unlocks $\textsf{r}(x)\vee \textsf{s}(y)$.

The second backtracking step happens after $\bot \mathbin {\leftarrow }\{\textsf{r}_2\}$ is derived from $\lnot \textsf{q}(x)$ and $\textsf{q}(y)\mathbin {\leftarrow }\{\textsf{r}_2\}$. Since both branches of the split $\textsf{s}_2$ have now been closed, the Switch rule is invoked, producing the model $(\mathcal J_0{\setminus }\{\lnot \textsf{r}_0,\lnot \textsf{s}_0\}) \cup \{\textsf{r}_0,\textsf{s}_0\}$. This unlocks $\lnot \textsf{p}(x)\vee \textsf{q}(y)$, and now only $\textsf{q}(y)\mathbin {\leftarrow }\{\textsf{r}_0\}$ is enabled in addition to the unlocked input clauses.

The $\textsf{r}_0$ branch is immediately closed by the generation of $\bot \mathbin {\leftarrow }\{\textsf{r}_0\}$ from $\textsf{q}(y)\mathbin {\leftarrow }\{\textsf{r}_0\}$ and $\lnot \textsf{q}(x)$.

Now, the resulting A-clause set contains the propositional clauses $\bot \mathbin {\leftarrow }\{\textsf{l}_0,\textsf{l}_2\}$, $\bot \mathbin {\leftarrow }\{\textsf{r}_2\}$, $\bot \mathbin {\leftarrow }\{\textsf{r}_0\}$ derived by inferences as well as $\bot \mathbin {\leftarrow }\{\textsf{s}_0,\lnot \textsf{l}_0,\lnot \textsf{r}_0\}$ and $\bot \mathbin {\leftarrow }\{\textsf{s}_2,\lnot \textsf{l}_2, \lnot \textsf{r}_2\}$ produced by Split. Clearly, it entails $\bot \mathbin {\leftarrow }\{\textsf{s}_0,\textsf{s}_2\}$. Since $\textit{fml}(\textsf{s}_0) = \textit{fml}(\textsf{s}_2) = {{\sim }\bot }$, at this point StrongUnsat derives $\bot$.

By following the strategy presented above, ${{\textsf{L}}}{{\textsf{S}}}$ closely simulates the original calculus, in the sense that it is possible to add and remove (or at least disable) exactly the same elements to the A-clause set as is done in the original, and in the same order. A subtle, inconsequential difference lies in the backtracking: Labeled splitting can move to a branch where $\bot$ is enabled, whereas our Switch rule requires that all propositional clauses are satisfied.

What about fairness? The above strategy helps achieve fairness by ensuring that there exists at most one limit point. It also uses locks in a well-behaved way. This means we can considerably simplify the notion of fairness for $\Longrightarrow _{{\textsf{L}}}$-derivations and obtain a criterion that is almost identical to, but slightly more liberal than, Fietzke and Weidenbach’s, thereby re-proving the completeness of labeled splitting.

For terminating derivations, their fairness criterion coincides with ours: Both require that the final A-clause set is locally saturated and all propositional clauses are satisfied by the interpretation. For diverging derivations, Fietzke and Weidenbach construct a limit subsequence $(\Phi '_i,\mathscr {N}'_i)_i$ of the derivation $(\Phi _i,\mathscr {N}_i)_i$ and demand that every persistent inference in it be made redundant, exactly as we do for $\Longrightarrow _{{\textsf{L}}}$-derivations. The subsequence consists of all states that lie on the split tree’s unique infinite branch. Therefore, this subsequence converges to a limit point of the full derivation. Locks are well behaved, with , because with the strategy above, once an A-clause is enabled on the rightmost branch, it remains enabled forever. Our definition of fairness allows more subsequences, although this is difficult to exploit without bringing in all the theoretical complexity of AVATAR.

#### Example 83

Alternating age-based and unrestricted heuristic selection is incomplete for labeled splitting just as it is for AVATAR (Example 58). To see why, start with the clause setand always select the negative literal if there is one. The prover begins by deriving $\textsf{p}(\textsf{s}(\textsf{a}), y)$ and $\textsf{r}(\textsf{a}) \vee \textsf{q}(y)$ using the age-based heuristic. Then it heuristically selects $\textsf{r}(\textsf{a}) \vee \textsf{q}(y)$ and splits it. In the left branch, where $\textsf{q}(y)$ is enabled, $\textsf{q}(\textsf{a})$ is locally redundant and locked. Before age-based selection allows the prover to derive $\bot$ from the clauses $\textsf{s}(x) \vee \lnot \textsf{q}(x), \textsf{q}(y),$ and $\lnot \textsf{s}(x),$ it will also have derived $\textsf{p}(\textsf{s}(\textsf{s}(\textsf{a})), y)$ and $\textsf{r}(\textsf{s}(\textsf{a})) \vee \textsf{q}(y).$ When the prover switches back to the right branch, it can heuristically select the newly derived disjunction and split it.

This process can be repeated to give rise to infinitely many splittable clauses of the form $\textsf{r}(\textsf{s}^i(\textsf{a})) \vee \textsf{q}(y)$. In this way, no inferences are ever performed in the rightmost branch, only splits. The clause $\textsf{q}(\textsf{a})$, which is necessary for a refutation, is never selected for inferences; most of the time, it is even locally redundant.

### SMT with Quantifiers

SMT solvers based on DPLL(*T*) [20] combine a SAT solver with theory solvers, each responsible for reasoning about a specific quantifier-free theory (e.g., equality, linear integer arithmetic). In the classical setup, the theories are decidable, and the overall solver is a decision procedure for the union of the theories. Some SMT solvers, including cvc5 [3], veriT [8], and Z3 [10], also support quantified formulas via instantiation at the expense of decidability.

Complete instantiation strategies have been developed for various fragments of first-order logic [16, 23, 24]. In particular, enumerative quantifier instantiation [24] is complete under some conditions. An SMT solver following such a strategy ought to be refutationally complete, but this has never been proved. Although SMT is quite different from the architectures we have studied so far, we can instantiate our framework to show the completeness of an abstract SMT solver. The model-guided prover ${{\textsf{M}}}{{\textsf{G}}}$ will provide a suitable starting point, since we will need neither ${\textsf{L}}$’s locking mechanism nor ${{\textsf{A}}}{{\textsf{V}}}$’s given clause procedure.

Let ${\textbf{F}}$ be the set of first-order ${\Sigma }$-formulas with a distinguished falsehood $\bot$. We represent the SMT solver’s underlying SAT solver by the $\textsc {Unsat}$ rule and complement it with an inference system $\textit{FInf}{}$ that clausifies formulas, detects inconsistencies up to theories excluding quantifiers, and instantiates quantifiers. For $\textit{FRed}$, we take an arbitrary instance of the standard redundancy criterion [2, Sect. 4.2.2]. It can be used to split disjunctions destructively and to simplify formulas. We define the “theories with quantifiers” calculus ${{\textsf{T}}}{{\textsf{Q}}}= (\textit{FInf}{}, \textit{FRed})$. For the consequence relations $\models$ and $|\!\!\!\approx$, we use entailment in the supported theories including quantifiers.

Some theories such as linear integer arithmetic are not compact and thus cannot directly be used for the consequence relation. Instead, we define $M \models _{LIA} N$ to be true if and only if there exist finite sets $M' \subseteq M$ and $N' \subseteq N$ such that  is valid modulo linear integer arithmetic. For finite sets, this relation coincides with noncompact entailment: If *M* is finite, then $M \models _{LIA} \bot$ if and only if *M* is inconsistent modulo linear integer arithmetic. Both completeness and soundness of a concrete prover are statements about the finite set of input formulas, so using a compactified version of the consequence relation is purely an implementation detail and poses no restriction.

The clausification rules work on logical symbols outside quantifiers; they derive *C* and *D* from a premise $C \wedge D$, among others. The theory rules can derive $\bot$ from some finite formula set *N* if , ignoring quantifiers; this triggers a model switch. Finally, the instantiation rules derive formulas $\textsf{p}(t)$ from premises $\forall x.\>\textsf{p}(x)$, where *t* is some ground term; the instantiation strategy determines which ground terms must be tried and in which order. A lot of complexity hidden in $\textit{FInf}{}$—such as purification and theory-specific data structures and algorithms—is taken as a black box.

As with AVATAR, the initial problem is expressed using ${\Sigma }$-formulas. We use the same approximation function as in AVATAR to represent formulas as assertions (Example 8). Abusing terminology slightly, let us call an A-formula $C \mathbin {\leftarrow }A$ a *subunit* if *C* is not a disjunction. Whenever a (ground) disjunction $C \vee D \mathbin {\leftarrow }A$ emerges, we immediately apply Split. This delegates clausal reasoning to the SAT solver. It then suffices to assume that ${{\textsf{T}}}{{\textsf{Q}}}$ is complete for subunits.

#### Theorem 84

**(Dynamic completeness)**  Assume ${{\textsf{T}}}{{\textsf{Q}}}$ is statically complete for subunit sets. Let $(\mathcal {J}_i, {{\mathscr {N}}}_i)_i$ be a fair $\Longrightarrow _{{{\textsf{M}}}{{\textsf{G}}}}$-derivation based on ${{\textsf{T}}}{{\textsf{Q}}}$. If  and ${{\mathscr {N}}}_\infty$ contains only subunits, then $\bot \in {{\mathscr {N}}}_j$ for some *j*.

#### Proof

The proof is analogous to that of Theorem 28. Because we only have conditional static completeness of $(\textit{FInf}{}, \textit{FRed})$, we need the assumption that ${{\mathscr {N}}}_\infty$ contains only subunits. $\square$

Care must be taken to design a practical fair strategy. Like AVATAR-based provers, SMT solvers will typically not perform all $\textit{SInf}{}$-inferences, not even up to $\textit{SRed}_\text {I}$. Given $\textsf{a}\approx \textsf{b} \mathbin {\leftarrow }\{\textsf{v}_0\}$, $\textsf{b}\approx c \mathbin {\leftarrow }\{\textsf{v}_1\}$, $\textsf{a} \approx \textsf{d} \mathbin {\leftarrow }\{\textsf{v}_2\}$, $\textsf{c} \approx \textsf{d} \mathbin {\leftarrow }\{\textsf{v}_3\}$, and $\textsf{a} \not \approx \textsf{c} \mathbin {\leftarrow }\{\textsf{v}_4\},$ an SMT solver will find only one of the conflicts $\bot \mathbin {\leftarrow }\{\textsf{v}_0, \textsf{v}_1, \textsf{v}_4\}$ or $\bot \mathbin {\leftarrow }\{\textsf{v}_2, \textsf{v}_3, \textsf{v}_4\}$ but not both. This leaves us in a similar predicament as with locking: A theory conflict might be nonredundant at the limit point, even though it is redundant at every point of the derivation. The SMT solver just happened to choose the wrong conflict every time.

#### Example 85

Consider the initial clause setEagerly applying quantifier instantiation, we get the instancesfor every $i \in {\mathbb {N}}$. The solver then starts in a model where each $[\textsf{a} > i]$ is true. Here it can derive the conflict $\bot \mathbin {\leftarrow }\{[\textsf{a} > 0]\}$. Then it switches to the next model where $[\textsf{a} > 0]$ is false, but $[\textsf{a}> 1], [\textsf{a} > 2]$, etc. are true, and derive the conflict $\bot \mathbin {\leftarrow }\{[\textsf{a} > 1]\}$.

Iterating this process, we see that all conflicts are of the form $[\textsf{a} > i]$ for some *i*. However, at the limit point— where $[\textsf{a} > i]$ is false for every *i*— none of these conflicts is enabled. The only conflict which exists at the limit point is between $\textsf{a} \approx 0$ and $\textsf{a} + 3 < 2$, and the solver never finds it because it detects a different conflict first.

For decidable theories, a practical fair strategy is to first clausify and detect theory conflicts and to instantiate quantifiers only if no other rules are applicable. A similar case analysis as in the proof of Lemma 77 works to establish fairness for this strategy.

First consider the case where quantifier instantiation is invoked infinitely often. Then there exists an infinite subsequence $(\mathcal J'_j, \mathscr {N}'_j)_j$ of states such that (1) $(\mathcal J'_j)_j$ converges to a limit point, and (2) no $\mathscr {N}'_j$ has a theory conflict. To prove the $\Longrightarrow _{{{\textsf{M}}}{{\textsf{G}}}}$-derivation fair, we need to show that $\iota \in \textit{FInf}{}((\mathscr {N}_\infty ) _{\mathcal J})$ implies $\iota \in \textit{FRed}_\text {I}((\mathscr {N}_i) _{\mathcal J})$ for every $\iota .$ If $\iota$ is a theory conflict or clausification inference, then its finitely many premises are in $\mathscr {N}'_j$ for some *j*, contradicting the strategy. Otherwise, $\iota$ is a quantifier instantiation. Here, it suffices to ensure that A-formulas that are enabled infinitely often at a quantifier instantiation step are also fully instantiated. (Just as with ${{\textsf{A}}}{{\textsf{V}}}$ provers, it is possible that not all limit points are saturated.)

Otherwise, quantifier instantiation is only invoked finitely often—either because every encountered model had a theory conflict, or because there was nothing to instantiate. Here, it suffices to assume that clausification is a strongly finitary simplification bound (which means that a formula can only be clausified in a finite number of ways). Under this assumption only finitely many base formulas will be derived; this implies that only a finite number of models will be considered. The last model will then be saturated due to the strategy.

There is also the question of model soundness. If the SMT solver starts with the ${\Sigma }$-formula set ${{\mathscr {N}}}_0$ and ends in a state $(\mathcal {J}_i, {{\mathscr {N}}}_i)$ with $\mathcal {J}_i \models ({{\mathscr {N}}}_i)_\bot$, we would like the solver to generate a model of $({{\mathscr {N}}}_i)_{\mathcal {J}_i}$, from which a model of ${{\mathscr {N}}}_0$ can be derived. This is possible if the solver performs only sound inferences and applies Approx systematically. Then $({{\mathscr {N}}}_i)_{\mathcal {J}_i}$ is fully exposed to the propositional level, and $\textit{fml}(\mathcal {J}_i)$ is a theory model of ${{\mathscr {N}}}_{\mathcal {J}_i}$ and therefore of ${{\mathscr {N}}}_0$.

#### Example 86

Consider an SMT solver equipped with the theory of uninterpreted functions and linear arithmetic. Let

$$\begin{aligned}\{ \forall x\, (\textsf{f}(x) \approx 0 \vee \textsf{g}(x) \approx 0){,}\qquad \textsf{f}(1) \approx 1{,}\qquad \textsf{g}(1) \approx 1 \}\end{aligned}$$

be the initial clause set. The SMT solver first considers the propositional model $\mathcal J_0 = \lnot {{\textbf {V}}}.$ There is no theory conflict, so the solver uses quantifier instantiation and clausification to derive $\textsf{f}(0) \approx 0 \mathbin {\leftarrow }\{\textsf{v}_0\}$, $\textsf{g}(0) \approx 0 \mathbin {\leftarrow }\{\textsf{v}_1\}$, and $\bot \mathbin {\leftarrow }\{\lnot \textsf{v}_0, \lnot \textsf{v}_1\}.$ We have $\mathcal J_0 \not \models \bot \mathbin {\leftarrow }\{\lnot \textsf{v}_0, \lnot \textsf{v}_1\}$, so the solver switches to the model $\mathcal J_1 = (\mathcal J_0 {\setminus } \{\lnot \textsf{v}_0\}) \cup \{\textsf{v}_0\}.$ There is still no theory conflict, so it instantiates a quantifier again, producing the A-clauses $\textsf{f}(1) \approx 0 \mathbin {\leftarrow }\{\textsf{v}_2\}$, $\textsf{g}(1) \approx 0 \mathbin {\leftarrow }\{\textsf{v}_3\}$, and $\bot \mathbin {\leftarrow }\{\lnot \textsf{v}_2, \lnot \textsf{v}_3\}.$ The solver now switches to $\mathcal J_2 = (\mathcal J_1 {\setminus } \{\lnot \textsf{v}_2\}) \cup \{\textsf{v}_2\}.$ It derives a theory conflict $\bot \mathbin {\leftarrow }\{\textsf{v}_2\}$ and switches to $\mathcal J_3 = (\mathcal J_2 {\setminus } \{\lnot \textsf{v}_3\}) \cup \{\textsf{v}_3\}.$ For this model, there is also a conflict, $\bot \mathbin {\leftarrow }\{\textsf{v}_3\}$, and the solver terminates by applying Unsat.

Our mathematization of AVATAR and SMT with quantifiers exposes their dissimilarities. With SMT, splitting is mandatory, and there is no subsumption or simplification, locking, or active and passive sets. And of course, theory inferences are *n*-ary and quantifier instantiation is unary, whereas superposition is binary. Nevertheless, their completeness follows from the same principles.

## Conclusion

Our framework captures splitting calculi and provers in a general way, independently of the base calculus. Users can conveniently derive a dynamic refutational completeness result for a splitting prover based on a given statically refutationally complete calculus. As we developed the framework, we faced some tension between constraining the SAT solver’s behavior and the saturation prover’s. It seemed preferable to constrain the prover, because the prover is typically easier to modify than an off-the-shelf SAT solver. To our surprise, we discovered counterexamples related to locking, formula selection, and simplification, which may affect Vampire’s AVATAR implementation, depending on the SAT solver and prover heuristics used. We proposed some restrictions, but alternatives could be investigated.

We found that labeled splitting can be seen as a variant of AVATAR where the SAT solver follows a strict strategy and propositional variables are not reused across branches. A benefit of the strict strategy is that locking preserves completeness. As for the relationship between AVATAR and SMT, there are some glaring differences, including that splitting is necessary to support disjunctions in SMT but fully optional in AVATAR. For future work, we could try to complete the picture by considering other related architectures [5–7, 11, 12].
